# Effects of Medium Cut-Off Versus High-Flux Hemodialysis Membranes on Biomarkers: A Systematic Review and Meta-Analysis

**DOI:** 10.1177/20543581211067090

**Published:** 2022-01-18

**Authors:** Maryam Kandi, Romina Brignardello-Petersen, Rachel Couban, Celina Wu, Gihad Nesrallah

**Affiliations:** 1 Department of Health Research Methods, Evidence & Impact, McMaster University, Hamilton, ON, Canada; 2 University of Toronto, ON, Canada; 3 Nephrology Program, Humber River Hospital, Toronto, ON, Canada

**Keywords:** Theranova, medium cut-off, expanded hemodialysis, large middle molecules, meta-analysis, dialysis outcomes

## Abstract

**Background:**

Medium cut-off (MCO) membranes enhance large middle-molecule clearance while selectively retaining molecules >45 000 Da.

**Objectives:**

We undertook a systematic review and meta-analysis comparing the effects of MCO versus high-flux membranes on biomarkers.

**Methods:**

We searched MEDLINE, Embase, CINAHL, Cochrane Library, and Web of Science from January 2015 to July 2020, and gray literature sources from 2017. We included randomized (RS) and nonrandomized studies (NRS) comparing MCO and high-flux membranes in adults (>18 years) receiving maintenance hemodialysis. We performed study selection, data extraction, and quality appraisals in duplicate and used the Grading of Recommendations Assessment, Development, and Evaluation framework. Outcomes included solute removal (plasma clearance or dialysate quantitation), reduction ratios, and predialysis serum concentrations for a range of prespecified large middle molecules.

**Results:**

We identified 26 eligible studies (10 RS and 16 NRS; N = 1883 patients; patient-years = 1366.3). The mean difference (MD) for albumin removal was 2.31 g per session (95% confidence interval [CI], 2.79 to 1.83; high certainty), with a reduction in predialysis albumin of −0.12 g/dl (95% CI, −0.16 to −0.07; *I*^2^ = 0%; high certainty) in the first 24 weeks, returning to normal (MD = −0.02 g/dl, 95% CI, −0.07 to −0.03; *I*^2^ = 56%; high certainty) after 24 weeks. We also found with high certainty that MCO dialysis resulted in a large increase (standardized mean difference [SMD]> 2.0 for all) in β2-microglobulin, κ- and λ-free light chains, and myoglobin removal, resulting in moderate (SMD > 0.5) to large (SMD > 0.8) reductions in predialysis concentrations for all of these solutes. Medium cut-off dialysis increased the reduction ratio for tumor necrosis factor-alpha (TNF-α) by 7.7% (95% CI, 4.7 to 10.6; moderate certainty), and reduced predialysis TNF-α by SMD −0.48 (95% CI, −0.91 to −0.04; moderate certainty). We found with moderate certainty that MCO dialysis had little to no effect on predialysis interleukin-6 (IL-6) plasma concentrations. Medium cut-off dialysis reduced mRNA expression of TNF-α and IL-6 in peripheral leukocytes by MD −15% (95% CI, −19.6 to −10.4; moderate certainty) and −8.8% (95% CI, −10.2 to −7.4; moderate certainty), respectively.

**Conclusion:**

Medium cut-off dialysis increases the clearance of a wide range of large middle molecules and likely reduces inflammatory mediators with a concomitant transient reduction in serum albumin concentration. The net effect of MCO dialysis on large middle molecules could translate into important clinical effects.

## Abrégé

**Contexte:**

Les membranes MCO (*Medium cut-off*) améliorent la clairance des moyennes molécules de masse moléculaire élevée tout en retenant sélectivement les molécules de plus de 45 000 Da.

**Objectifs:**

Nous avons entrepris une revue systématique et une méta-analyse comparant les effets des membranes MCO et des membranes à perméabilité élevée sur certains biomarqueurs.

**Méthodologie:**

Nous avons effectué des recherches dans MEDLINE, EMBASE, CINAHL, Cochrane Library et Web of Science entre janvier 2015 et juillet 2020, et dans des sources de littérature grise de 2017. Nous avons inclus les études randomisées (ÉR) et non randomisées (ÉNR) comparant les membranes MCO et les membranes à perméabilité élevée chez les adultes recevant une hémodialyse d’entretien. Nous avons procédé à la sélection des études, à l’extraction des données et à l’évaluation de la qualité en duplicata, puis nous avons utilisé la méthodologie GRADE (*Grading of Recommendations Assessment, Development and Evaluation*). Les résultats comprenaient l’élimination du soluté (clairance plasmatique ou quantification du dialysat), les rapports de réduction et les concentrations sériques prédialyse pour une gamme de moyennes molécules à masse moléculaire élevée prédéterminées.

**Résultats:**

Nous avons répertorié 26 études admissibles (10 ÉR, 16 ÉNR; *n* = 1 883 patients; 1 366,3 années-patients). La différence moyenne (DM) pour l’élimination de l’albumine était de 2,31 g par séance (IC 95 % : 2,79 à 1,83; haute certitude), avec une réduction de l’albumine prédialyse de -0,12 g/dl (IC 95 % : -0,16 à -0,07; *I*^2^ = 0 %; haute certitude) au cours des 24 premières semaines, et un retour à la normale (DM = -0,02 g/dl; IC 95 % : -0,07 à -0,03; *I*^2^ = 56 %; haute certitude) après 24 semaines. Nous avons constaté, avec une grande certitude, que la dialyse MCO entraînait une élimination importante de β2-microglobuline, chaînes légères κ- et λ- et de myoglobine (différence moyenne standardisée [DMS] > 2,0 pour toutes), ce qui s’est traduit par des réductions modérées (DMS > 0,5) à importantes (DMS > 0,8) des concentrations prédialyse pour tous ces solutés. La dialyse MCO a haussé le taux de réduction du TNF-α de 7,7 % (IC 95 % : 4,7 à 10,6; certitude modérée) et réduit le TNF-α prédialyse (DMS = -0,48; IC 95 % : -0,91 à -0,04; certitude modérée). Nous avons constaté, avec une certitude modérée, que la dialyse MCO n’a que peu ou pas d’effet sur les concentrations plasmatiques d’IL-6 prédialyse. La dialyse MCO a réduit l’expression de l’ARNm du TNF-α et d’IL-6 dans les leucocytes périphériques avec une DM de -15 % (IC 95 % : -19,6 à -10,4; certitude modérée) et de -8,8 % (IC 95 % : -10,2 à -7,4; certitude modérée) respectivement.

**Conclusion:**

La dialyse MCO augmente la clairance d’une vaste gamme de moyennes molécules de haute masse moléculaire et semble réduire les médiateurs inflammatoires avec une réduction transitoire concomitante de la concentration en albumine sérique. L’effet net de la dialyse MCO sur les moyennes molécules de haute masse moléculaire pourrait se traduire par des effets cliniques importants.

## Introduction

Uremic toxins exhibit a wide range of physiochemical properties leading to diverse molecular and cellular level effects that contribute to morbidity and mortality among patients with end-stage renal disease. Earlier membrane technologies that provided small solute (<500 Da) clearance were supplanted by high-flux membranes that were specifically engineered to enhance β2-microglobulin removal but provide minimal diffusive clearance above 15 kDa. Solutes above the molecular weight cut-off of high-flux membranes—so-called large middle molecules (15-60 kDa)—comprised a diverse group of biomarkers including cytokines, adipokines, hormones, and other proteins that are implicated in chronic inflammation, cardiovascular disease, and secondary immunodeficiency.^1^ Technologies that expand the range of dialyzable solutes within this range therefore may represent an opportunity to narrow the “clearance gap” between dialysis membranes and healthy kidneys.

A novel medium cut-off (MCO) membrane (Theranova 400/500, Baxter Healthcare, Deerfield, Illinois) removes large middle molecules while selectively excluding albumin and other large molecules above 45 kDa.^2^ This is achieved by new membrane engineering processes that produce larger pores falling within a narrow diameter distribution, leading to a “steep” sieving curve, with high selectivity at the target “cut-off” molecular weight, thereby optimizing the membrane’s depuration profile, and potentially leading to better outcomes.

We conducted a systematic review and meta-analysis on the comparative effects of MCO versus high-flux membranes in hemodialysis and have reported clinical outcomes in a separate manuscript.^3^ This report describes the effects of MCO dialysis on selected biomarkers of known prognostic importance, falling within the expanded range of molecular weights to which MCO membranes are permeable.

## Methods

### Protocol and Registration

We registered our protocol with PROSPERO (CRD42020204636; Appendix A, with amendments), and prepared this article in accordance with Preferred Reporting Items for Systematic Reviews and Meta-Analyses. Detailed methods are in Appendix B.

### Eligibility Criteria

We included randomized and nonrandomized studies, from 2015, published in any language, enrolling adult outpatients receiving maintenance hemodialysis with MCO membranes and related prototypes, excluding studies of high cut-off and “super high-flux” membranes. Eligible comparators were high-flux membranes used for hemodialysis, excluding studies of convective modalities. Prespecified outcomes are in Appendix B. Selected biomarkers included albumin-related measures, representative middle molecules, and inflammatory markers. Given the breadth of analytes included in the published literature, we selected a priori a range of large middle molecules that were most frequently reported in the subset of studies identified during our pilot search that have established prognostic significance, and that spanned the entire range of applicable molecular weights.

### Information Sources

We searched MEDLINE, Embase, CINAHL, the Cochrane Library, and Web of Science from January 2015 to July 2020. We included abstracts from prespecified major conferences to 2017. We cross-referenced our search results with a database provided by the manufacturer.

### Search

Hemodialysis and MCO membranes were the main search concepts. We combined synonyms for each using the OR operator, then combined these concepts using AND operator. Our final search strategy is in Appendix C.

### Study Selection

We used EndNote X9.3 for deduplication and DistillerSR for screening in duplicate.

### Data Collection Process

Working independently, reviewers extracted data into standard forms with verification by a second reviewer.

### Data Items

Details are in Appendix A. Extracted measures included solute removal, removal ratios (adjusted for the hemoconcentrating effects of ultrafiltration), and (equilibrated) predialysis serum solute concentrations.

### Risk of Bias in Individual Studies

Two reviewers independently assessed risk of bias using the Cochrane “RoB” tool version 2 for randomized studies (https://www.riskofbias.info/welcome/rob-2-0-tool)^4^ and the ROBINS-I tool for nonrandomized studies (https://www.riskofbias.info/welcome/home).^5^ We did not consider an open-label design serious risk of bias for outcome measurement for the laboratory-based measures included in this report.

### Summary Measures

We extracted change scores and corresponding standard errors and used *P* values to impute the standard error for change where required, then calculated the mean difference (MD) between groups. Where change scores were not available, we used final values instead. Where units of measure differed for a given outcome, we calculated standardized mean differences (SMD) as Cohen’s *d* where values above 0.2, 0.5, and 0.8 correspond to small, medium, and large effects, respectively.^6^

### Synthesis of Results

We pooled randomized and nonrandomized studies separately using random effects models with the generic inverse variance method for weighting the studies. We used fixed-effects models when pooling 2 studies to avoid overweighting where necessary. We assessed heterogeneity with the *I*^2^ statistic.

### Risk of Bias Across Studies

Where possible, we used funnel plots to assess for publication bias.

### Additional Analyses

We used subgroup analyses to explore heterogeneity. Study duration was a prespecified subgroup as we anticipated that the effects of enhanced large middle-molecule clearance could be cumulative over time. The method of measurement of solute removal (blood-side vs dialysate quantitation) and baseline removal ratio were subgroups that we identified post hoc. We used the median value for follow-up duration and removal ratio as the cut-point for each subgroup analysis. We also used subgroup analysis for selected outcomes where we considered differences between short- versus long-term effects as potentially clinically important, for example, for effects on serum albumin.

### Certainty of Evidence

We assessed the certainty of evidence for each outcome using the GRADE approach and summarized these assessments in a Summary of Findings Table using GRADEpro: https://gdt.gradepro.org.^7^ Evidence certainty was rated as very low, low, moderate, or high. Because we used ROBINS-I to assess risk of bias for nonrandomized studies, both randomized studies and nonrandomized studies started with a high certainty rating and were downgraded 1, 2, or 3 levels for risk of bias,^8,9^ inconsistency,^10^ indirectness,^11^ imprecision,^12^ or publication bias.^13^ We did not downgrade for inconsistency when the effect estimates from all included studies were large, that is, SMDs ≥ 0.8, because heterogeneity of effects ranging between large and very large would not reduce our certainty in the pooled estimate. We used validated algorithms embedded in the GRADEpro platform to generate informative narrative statements describing treatment effects and their associated levels of certainty and used the generated phrasing throughout this article to guard against subjective judgments (Tables 2-4, column labeled “What Happens”).^14^ For estimates based on the SMD, we used descriptors for effect sizes based on standard thresholds for interpreting Cohen’s *d*, as described above.

## Results

### Study Selection

**Figure 1.**
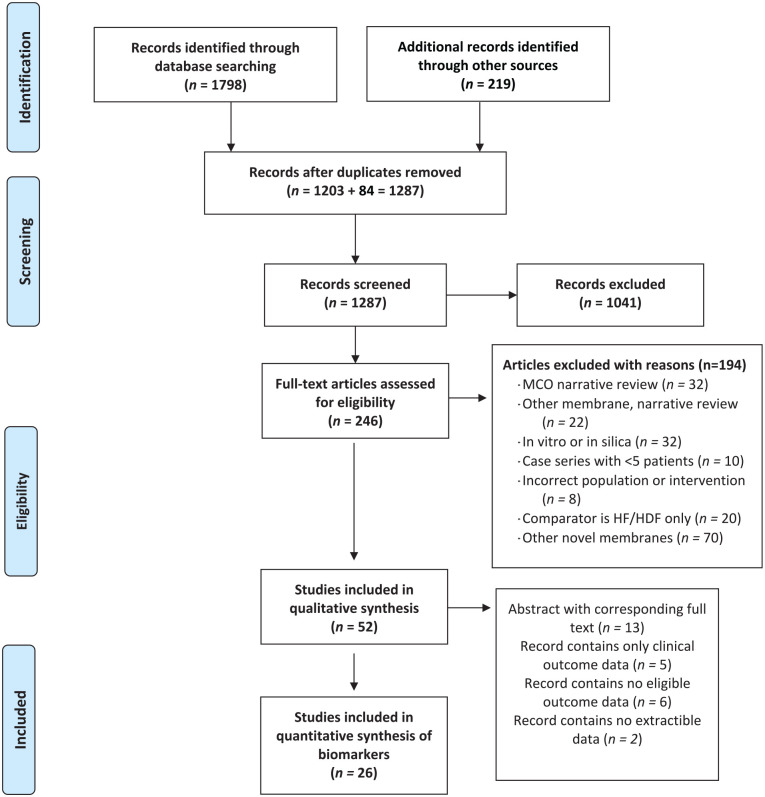
Study inclusion flow diagram. *Note*. MCO = medium cut-off; HF = high-flux; HF/HDF = hemofiltration/hemodiafiltration.

Figure 1 shows study selection details. We included 26 studies in this review of biomarkers,^15-40^ excluding 5 reports of exclusively clinical outcomes.^41-45^ Groupings of related citations are in Appendix D.

### Study Characteristics

Among 26 unique studies included in this review, 10 were randomized trials,^16-24,44^ including 2 parallel arm randomized studies^15,16^ and 8 crossover studies that included 1883 participants followed for a total of 1366.3 person-years. Among the nonrandomized studies, 3 were cohort studies,^25-27^ and the remainder used before-after designs. All randomized studies were published as peer-reviewed full texts; 9 nonrandomized studies were available as abstracts,^26,28-30,32-35,37^ Details of patient, study design, and intervention characteristics are in Table 1. Theranova was the only MCO membrane described in eligible studies. All the included studies enrolled patients who were on maintenance hemodialysis and were without acute cardiovascular or infectious complications for at least 3 months. Study participants from diverse geographies underwent thrice-weekly hemodialysis with conventional prescriptions and anticoagulation (held constant throughout) using Theranova 400/500 or high-flux membranes.

**Table 1.** 

1st author	Publication type	Country (number of centers)	Number of participants enrolled (number analyzed)	Weeks follow-up (baseline [HF] + intervention [MCO-HD])	Mean age ± SD (years)	% Male	Interventions	Reported outcomes
Randomized studies								
Parallel arm studies								
Lim et al.^15,44^	Full text	Korea (1)	50 (49)	12	I: 62.2 ± 13.7 C: 63.8 ± 15.2	66%	I: Theranova 400 C: FxCordiax 80 or 60	Survival, QoL (KDQOL), pruritus, adverse events, ERI, iron use, albumin (predialysis), MM
Weiner et al.^16^	Full text	USA (21)	172 (130)	24	59 ± 13	39%	I: Theranova 400 C: Elisio-17H	Survival, hospitalization, QoL (KDQOL, EQ-5D-5L), albumin (predialysis), MM
Randomized crossover trials								
Belmouaz et al.^17^	Full text	France (1)	40	26 (13/arm)	75.5 ± 9.9	70%	I: Theranova 500 C: Elisio 21H	Survival, ERI, iron utilization, albumin RR, albumin (predialysis), MM
Cordeiro et al.^18^	Full text	Brazil (1)	16	8 (4/arm)	40.7 ± 13.5	69%	I: Theranova 400 C: PS (Diacap)	Albumin (predialysis), MM
Kirsch et al. (study 1)^19^	Full text	Austria (1)	19	2 (1/arm)	55.4 ± 13.4	63%	I: Prototype “AA” C: FxCordiax 80VR	Albumin loss, MM
Kirsch et al. (study 2)^19^	Full text	Germany (1)	20	2 (1/arm)	65.4 ± 12.2	80%	I: Prototype “AA” C: FxCordiax 80VR	Albumin loss, MM
Maduell et al.^21^	Full text	Spain (1)	21	2 (1/arm)	63.2 ± 16	82%	I: Theranova 400 C: FxCordiax 80	Albumin loss, albumin RR, MM
Maduell et al.^20^	Full text	Spain (1)	21	2 (1/arm)	65.4 ± 13	76%	I: Theranova 400 C: Helixone	Albumin loss, albumin RR, MM
Santos et al.^22^	Full text	Spain (1)	13	2 (1/arm)	60.1 ± 4.6	92%	I: Theranova 500 C: FxCordiax 80VR	Bleeding, extracorporeal circuit clotting, aPTT, anti-Xa
Sevinc et al.^23^	Full text	Turkey (2)	52 (42)	26 (13/arm)	56.4 (median)	58%	I: Theranova 500 C: FxCordiax 80	Adverse events, albumin (predialysis), MM, inflammatory markers
Zickler et al.^24^	Full text	Germany (2)	50 (47)	4 (+8-week extension study)	I: 58.1 ± 16.6 C: 59.8 ± 16.5	38%	I: MCO-Ci400 C: Revaclear 400	Survival, adverse events, CRP, albumin (predialysis), MM, inflammatory markers
Nonrandomized studies								
Cohort studies								
Cho et al.^25^	Full text	Korea (1)	57 (57)	52	I: 53.7 ± 10.9 C: 56.4 ± 10.4	58%	I: Theranova 400 C: FxCordiax 80	Survival, ERI, MM, cell-free hemoglobin
Ostojic and Markovic^26^	Abstract	Serbia (1)	10	52	I: 63.2 ± 7 C: 63 ± 6	60%	I: Theranova 500 C: Polysulfone	MM, hemoglobin
Yeter et al.^27^	Full text	Turkey (1)	47 (42)	26	52.9 ± 16	63%	I: Theranova 400 C: Cordiax 800	Survival, ERI, iron utilization, CRP, albumin (predialysis)
Before-after studies								
Albrizio et al.^28^	Abstract	Italy (1)	8	2 (1/arm)	78 ± 14	25%	I: Theranova 400 C: HF	Adverse events, myoglobin
Baharani et al.^29^	Abstract	England (1)	8	9 (1 + 8)	71 ± 11.8	75%	I: Theranova 400 C: FxCordiax 60, 80	Adverse events, MM
Baharani et al.^29^	Abstract	England (1)	18	9 (1 + 8)	73 ± 16.7	78%	I: Theranova 400 C: Revaclear 300	Adverse events, MM
Bove et al.^30^	Abstract	Italy (1)	8	2 (1/arm)	NR	NR	I: Theranova 400 C: Cordiax FX80	Albumin loss, MM
Bunch et al.^a 31^	Full text	Colombia (1)	992 (638)	52	60 ± 15	62%	I: Theranova C: HF	Survival, hospitalization, safety, albumin (predialysis)
Cantaluppi et al.^32^	Abstract	Italy (multiple)	41	26	67.6 ± 13.4	NR	I: Theranova 400 C: HF	CRP, albumin (predialysis), MM
Celik et al.^33^	Abstract	Italy (1)	8	2 (1/arm)	66 ± 13	NR	I: Theranova C: HF	Albumin RR, MM inflammatory markers, CRP (RR only)
D’Achiardi et al.^34^	Abstract	Colombia (multiple)	52 (41)	24	61 ± 13	65%	I: MCO C: HF	Adverse events, albumin (predialysis), MM inflammatory markers, CRP
Gallo^35^	Abstract	Italy (1)	15	39	60.5 ± 12	80%	I: Theranova 400 C: HF	ERI, CRP, albumin (predialysis), MM
Garcia-Prieto et al.^36^	Full text	Spain (1)	18	3 (1/arm)	65 ± 13	50%	I: Theranova 500 C: FxCordiax 80VR	Adverse events, albumin loss and RR, MM
Gernone et al.^37^	Abstract	Italy (1)	11 (11)	52	70.8 ± 9	73%	I: Theranova C: HF	PCS, MCS, ERI, albumin (predialysis), MM
Kim et al.^38^	Full text	Korea (1)	6 (6)	3 (1/arm)	66.1 ± 9.1	100%	I: Theranova 400 C: Rexeed-21A	Adverse events, albumin loss, albumin RR, MM
Krishnasamy et al.^39^	Full text	Australia and New Zealand (9)	89 (79)	32 (4 + 28)	66 ± 14	62%	I: Theranova 400 C: Revaclear 400	Adverse events, QoL, RLS, ERI, CRP, albumin (predialysis)
Crossover studies								
Cozzolino et al.^40^	Full text	Italy (1)	21 (20)	26 (13 + 13)	71 ± 13	76%	I: Theranova 400 C: FX8, FX10, FX80, FX100, BK1.6, BG2.1	Survival, hospitalization, infection, albumin (predialysis), inflammatory markers

*Note.* MCO-HD = medium cut-off hemodialysis; KDQOL = Kidney Disease Quality of Life instrument; ERI = erythropoiesis resistance index; MM = middle-molecules (removal, reduction ratios, or predialysis serum levels); EQ-5D-5L = EuroQol 5-Dimension Questionnaire; RR = reduction ratio; PS = polysulfone; aPTT = activated partial thromboplastin time; MCO = medium cut-off; CRP = C-reactive protein; HF, high-flux (not otherwise specified); NR = not reported; PCS = physical component summary; MCS = mental component summary; PMMA = polymethylmethacrylate; RLS = restless legs syndrome.

a Single-arm MCO-HD data without comparator group for survival and hospitalization outcomes; not amenable to meta-analysis.

Studies reported 3 main treatment effects for biomarkers: (1) removal (or reduction) ratio, analogous to the familiar urea reduction ratio, that is, the predialysis to postdialysis difference in a solute’s concentration divided by the predialysis value and corrected for the hemoconcentrating effects of ultrafiltration; (2) mass removal determined either through blood-side measurements (reported as plasma clearance in ml/min) or through direct dialysate quantitation (g or mg removed per hemodialysis session); and (3) predialysis (equilibrated) solute concentrations.

### Risk of Bias Within Studies

Attrition was the primary form of bias within studies, as is typical with dialysis study populations.

### Synthesis of Results

Effect estimates and their certainty ratings with explanatory footnotes are in Summary of Findings Tables (Tables 2-4). Where the certainty of estimates arising from randomized and nonrandomized studies differed, we present only the estimate of higher certainty. A comprehensive Summary of Findings Table is in Appendix G. Unless stated otherwise, we describe overall effects below, with details of subgroup effects in Tables 2 to 4 and in forest plots in Appendix E.

**Table 2.** Summary of Findings—Albumin-Related Measures.

Outcome No. of participants (studies)	Anticipated absolute effects (95% CI)		Certainty	What happens
	Without MCO-HD	Difference		
Albumin loss (g) Follow-up: 2 weeks No. of participants: 230 (5 RS)	The mean albumin loss ranged from 0.2 to 0.56 g	MD 2.31 g higher (2.79 higher to 1.83 higher)	⨁⨁⨁⨁ High	MCO-HD increases albumin loss slightly.
Albumin reduction ratio (%) Follow-up: range, 2-26 weeks No. of participants: 162 (3 RS)	The mean albumin reduction ratio ranged from 7% to 11%	MD 2.39% higher (3.68 higher to 1.11 higher)	⨁⨁⨁⨁ High	MCO-HD increases albumin reduction ratio slightly.
Predialysis serum albumin (g/dl)Subgroup with <24-week follow-up Follow-up: range, 8-13 weeks No. of participants: 305 (5 RS)	The mean predialysis serum albumin ranged from 3.79 to 3.94 g/dl	MD 0.12 g/dl lower (0.17 lower to 0.07 lower)	⨁⨁⨁⨁ High	MCO-HD reduces predialysis serum albumin slightly over the short term (<24 weeks).
Predialysis serum albumin (g/dl)Subgroup with ≥24-week follow-up Follow-up: 24 weeks No. of participants: 129 (1 RS)	The mean predialysis serum albumin was 4.1 g/dl	MD 0 g/dl (0.1 lower to 0.1 higher)	⨁⨁⨁◯ Moderate^a^	MCO-HD likely results in little to no difference in predialysis serum albumin after 24 weeks of treatment.
Predialysis serum albumin (g/dl)Subgroup with ≥24-week follow-up Follow-up: range, 24-52 weeks No. of participants: 2010 (7 NRS)	The mean predialysis serum albumin (g/dl)—subgroup with ≥4-month follow-up ranged from 3.1 to 4.05 g/dl	MD 0.02 g/dl lower (0.08 lower to 0.04 higher)	⨁⨁⨁◯ Moderate^a^	MCO-HD likely results in little to no difference in predialysis serum albumin after 24 weeks of treatment.
Predialysis serum albumin (g/dl) Subgroup analysis—RS and NRS with <24-week follow-up Follow-up: range, 8-13 weeks No. of participants: 325 (5 RS, 1 NRS)	The mean predialysis serum albumin ranged from 3.76 to 3.94 g/dl	MD 0.12 g/dl lower (0.16 lower to 0.07 lower)	⨁⨁⨁⨁ High	MCO-HD reduces predialysis serum albumin slightly within the first 24 weeks of follow-up.
Predialysis serum albumin (g/dl) Subgroup analysis—RS and NRS with ≥24-week follow-up Follow-up: range, 24-52 weeks No. of participants: 2139 (1 RS, 7 NRS)	The mean predialysis serum albumin ranged from 3.10 to 4.05 g/dl	MD 0.02 g/dl lower (0.07 lower to 0.03 higher)	⨁⨁⨁⨁ High	MCO-HD results in little to no difference in predialysis serum albumin after 24 weeks of follow-up.

*Note.* CI = confidence interval; MCO-HD = medium cut-off hemodialysis; MD = mean difference; NRS = nonrandomized study; RS = randomized study.

a Estimate prone to risk of bias due to patient attrition.

**Table 3.** Summary of Findings—Middle Molecules.

Outcome No. of participants (studies)	Anticipated absolute effects (95% CI)		Certainty	What happens
	Without MCO-HD	Difference		
β2M removal (mg) Follow-up: range, 2-8 weeks No. of participants: 152 (4 RS)	-	SMD 1.83 SD higher (0.02 higher to 3.64 higher)	⨁⨁⨁⨁ High^a^	MCO-HD results in a large increase in β2M removal.
β2M removal (mg) No. of participants: 16 (1 NRS)	-	SMD 1.4 SD higher (0.42 higher to 2.38 higher)	⨁⨁⨁⨁ High^b,c^	MCO-HD results in a large increase in β2M removal.
β2M reduction ratio (%) Follow-up: range, 2-26 weeks No. of participants: 323 (7 RS)	The mean β2M reduction ratio (%) ranged from 46% to 77%	MD 8.0% higher (2.8 higher to 13.2 higher)	⨁⨁⨁⨁ High^d^	MCO-HD increases β2M reduction ratio.
Predialysis β2M Subgroup with <12-week follow-up Follow-up: 8 weeks No. of participants: 32 (1 RS)	-	SMD 0.36 SD higher (0.33 lower to 1.06 higher)	⨁⨁◯◯ Low^e^	MCO-HD may result in little to no difference in predialysis β2M over the short term (<3 months).
Predialysis β2M Subgroup with ≥12-week follow-up Follow-up: range, 12-26 weeks No. of participants: 403 (5 RS)	-	SMD 0.54 SD lower (1 lower to 0.08 lower)	⨁⨁⨁◯ Moderate^f^	MCO-HD likely reduces predialysis β2M after 3 months of treatment.
Predialysis β2M Follow-up: range, 24-52 weeks No. of participants: 438 (6 NRS)	-	SMD 0.43 SD lower (0.84 lower to 0.002 lower)	⨁⨁⨁⨁ high	MCO-HD reduces predialysis β2M slightly.
Myoglobin removal Follow-up: 2 weeks No. of participants: 120 (3 RS)	-	SMD 2.9 SD higher (1.31 higher to 4.49 higher)	⨁⨁⨁⨁ high	MCO-HD likely results in a large increase in myoglobin removal.
Myoglobin reduction ratio (%) Follow-up: range, 2-26 weeks No. of participants: 242 (5 RS)	The mean myoglobin reduction ratio (%) ranged from 8% to 45%	MD 30.26% higher (15.5 higher to 45.03 higher)	⨁⨁⨁⨁ High^g^	MCO-HD results in large increase in myoglobin reduction ratio.
Myoglobin reduction ratio (%) Follow-up: range, 2-52 weeks No. of participants: 118 (6 NRS)	The mean myoglobin reduction ratio (%) ranged from 12% to 44%	MD 27.62% higher (24.29 higher to 30.95 higher)	⨁⨁⨁⨁ High	MCO-HD results in large increase in myoglobin reduction ratio.
Predialysis myoglobin Follow-up: 26 weeks No. of participants: 130 (2 RS)	-	SMD 0.51 SD lower (0.85 lower to 0.16 lower)	⨁⨁⨁◯ Moderate^h^	MCO-HD likely reduces predialysis myoglobin.
Predialysis myoglobin Follow-up: 26 weeks No. of participants: 82 (1 NRS)	-	SMD 0.12 SD lower (0.55 lower to 0.31 lower)	⨁⨁⨁◯ Moderate^b^	MCO-HD likely reduces myoglobin prehemodialysis slightly.
Kappa FLC removal Follow-up: 2 weeks No. of participants: 78 (2 RS)	-	SMD 3.89 SD higher (3.45 higher to 4.33 higher)	⨁⨁⨁⨁ High	MCO-HD results in large increase in kappa FLC removal.
Kappa FLC reduction ratio (%) Follow-up: range, 2-26 weeks No. of participants: 249 (5 RS)	The mean kappa FLC reduction ratio (%) ranged from 53% to 72%	MD 14.85% higher (8.27 higher to 21.43 higher)	⨁⨁⨁⨁ High^g^	MCO-HD results in large increase in kappa FLC reduction ratio.
Predialysis kappa-FLC Follow-up: range, 12-26 weeks No. of participants: 403 (5 RS)	-	SMD 0.39 SD lower (0.61 lower to 0.16 lower)	⨁⨁⨁⨁ High	MCO-HD reduces predialysis kappa FLC slightly.
Lambda FLC removal Follow-up: 2 weeks No. of participants: 118 (3 RS)	-	SMD 2.16 SD higher (1.8 higher to 2.52 higher)	⨁⨁⨁⨁ High	MCO-HD results in large increase in lambda FLC removal.
Lambda FLC removal Follow-up: range, 2-3 weeks No. of participants: 130 (2 NRS)	-	SMD 3.71 SD higher (2.97 higher to 4.45 higher)	⨁⨁⨁⨁ High	MCO-HD results in large increase in lambda free light chain removal.
Lambda FLC reduction ratio (%) Follow-up: range, 2-26 weeks No. of participants: 450 (7 RS)	The mean lambda FLC reduction ratio (%) ranged from 13% to 41%	MD 20.85% higher (15.53 higher to 26.16 higher)	⨁⨁⨁⨁ High	MCO-HD increases lambda-FLC reduction ratio.
Predialysis lambda-FLC Follow-up: range, 12-26 weeks No. of participants: 402 (5 RS)	-	SMD 0.53 SD lower (0.9 lower to 0.17 lower)	⨁⨁⨁⨁ High^i^	MCO-HD reduces predialysis lambda-FLC.
Predialysis lambda-FLC Follow-up: range, 24-52 weeks No. of participants: 398 (4 NRS)	-	SMD 0.34 SD lower (0.54 lower to 0.14 lower)	⨁⨁⨁⨁ High	MCO-HD reduces lambda free light chain prehemodialysis slightly.

*Note.* CI = confidence interval; MCO-HD = medium cut-off hemodialysis; β2M = β2-microglobulin; RS = randomized study; SMD = standardized mean difference; SD = standard deviation; NRS = nonrandomized study; MD = mean difference; FLC = free light chains.

a *I*^2^ = 97%, but fully explained by measurement method—removal was higher when measured by plasma clearance versus dialysate quantitation.

b Small overall sample size; optimal information size criterion not met.

c SMD >0.8 considered a large treatment effect. Rated up 1 level.

d Although *I*^2^ was 99%, heterogeneity was explained by baseline removal ratio (larger effect if removal ratio was <70%), and was further explained by study duration (effect was attenuated with long-term treatment).

e Downgraded 2 levels for imprecision with only very small sample size and confidence interval crossing no effect.

f *I*^2^ > 50% and confidence intervals do not overlap.

g Inconsistency explained by baseline removal ratio such that studies with lower baseline RR had larger effects with MCO-HD.

h *I*^2^ = 82% with opposite directions of effect.

i Inconsistency explained by duration of follow-up with a larger treatment effect with long-term treatment.

**Table 4.** Summary of Findings—Inflammatory Markers and Cytokines.

Outcome No. of participants (studies)	Anticipated absolute effects (95% CI)		Certainty	What happens
	Without MCO-HD	Difference		
IL-6 reduction ratio (%) Follow-up: 26 weeks No. of participants: 80 (1 RS)	The mean IL-6 reduction ratio (%) was 9.5%	MD 0.2% lower (3.44 lower to 3.04 higher)	⨁⨁⨁◯ Moderate^a^	MCO-HD likely results in little to no difference in IL-6 reduction ratio (%).
Predialysis IL-6 Follow-up: range, 12-26 weeks No. of participants: 354 (4 RS)	-	SMD 0.04 SD higher (0.17 lower to 0.25 higher)	⨁⨁⨁◯ Moderate^b^	MCO-HD likely results in little to no difference in predialysis IL-6.
IL-6 mRNA expression Follow-up: 12 weeks No. of participants: 46 (1 RS)	The mean IL-6 expression was 100%	MD 8.8 % lower (10.2 lower to 7.4 lower)	⨁⨁⨁◯ Moderate^a^	MCO-HD likely reduces IL-6 expression.
TNF-α reduction ratio (%) Follow-up: 26 weeks No. of participants: 80 (1 RS)	The mean TNF-α reduction ratio (%) was 26%	MD 7.67% higher (4.7 higher to 10.64 higher)	⨁⨁⨁◯ Moderate^a^	MCO-HD likely increases TNF-α reduction ratio.
TNF-α predialysis Follow-up: range, 12-26 weeks No. of participants: 304 (3 RS)	-	SMD 0.48 SD lower (0.91 lower to 0.04 lower)	⨁⨁⨁◯ Moderate^a^	MCO-HD likely reduces predialysis TNF-α.
TNF-α mRNA expression Follow-up: 12 weeks No. of participants: 46 (1 RS)	The mean TNF-α expression was 100%	MD 15 % lower (19.6 lower to 10.4 lower)	⨁⨁⨁◯ Moderate^a^	MCO-HD likely reduces TNF-α expression.
C-reactive protein Follow-up: 12 weeks No. of participants: 145 (2 RS)	-	SMD 0.04 SD higher (0.37 lower to 0.29 higher)	⨁⨁⨁◯ Moderate^a^	MCO-HD likely results in little to no difference in C-reactive protein.
C-reactive protein Follow-up: range, 26-52 weeks No. of participants: 1940 (5 NRS)	-	SMD 0 SD (0.23 lower to 0.22 higher)	⨁⨁⨁⨁ High	MCO-HD results in little to no difference in C-reactive protein.

*Note.* CI = confidence interval; MCO-HD = medium cut-off hemodialysis; IL-6 = interleukin-6; RS = randomized study; MD = mean difference; SMD = standardized mean difference; SD = standard deviation; TNF-α = tumor necrosis factor-alpha; NRS = nonrandomized study.

a Small overall sample size; optimal information size criterion not met.

b Small overall sample size and the confidence interval includes no effect.

#### Albumin

Albumin-related measures are in Table 2. The pooled estimate from 5 randomized studies showed greater albumin removal with MD 2.31 g per session (95% confidence interval [CI], 2.79 to 1.83; high certainty) and a higher reduction ratio (3 randomized studies) 2.39% (95% CI, 3.68 to 1.11; high certainty) with MCO dialysis, with similar results in nonrandomized studies. Subgroup analyses examining short-term (<24 weeks) versus long-term follow-up (≥24 weeks) were of lower certainty; hence, we combined all available data from randomized studies and nonrandomized studies and found with high certainty that MCO dialysis reduced predialysis albumin by −0.12 g/dl (95% CI, −0.16 to −0.07; *I*^2^ = 0%) over the short term. Over long-term follow-up (24-52 weeks), MCO dialysis had a trivial to no effect on serum albumin levels with MD −0.02 g/dl (95% CI, −0.07 to −0.03; *I*^2^ = 56%; high certainty). No studies reported hypoalbuminemia that required albumin infusion or discontinuation of treatment with MCO membranes.

#### Middle molecules

Effects on middle molecules are in Table 3.

##### β2-microglobulin (11.8 kDa)

Pooling 4 randomized studies, we found that MCO dialysis results in a large increase in β2-microglobulin removal with SMD 1.83 (95% CI, 0.02 to 3.64; high certainty) with similar results from nonrandomized studies. The pooled reduction ratio from 7 randomized studies was higher with MCO dialysis with MD 8.0% (95% CI, 2.8 to 13.2; high certainty). Medium cut-off dialysis lowered predialysis β2-microglobulin to a moderate extent with SMD −0.41 (95% CI, −0.86 to −0.03; moderate certainty) with results from 6 randomized studies downgraded for inconsistency with high certainty of trivial to no effect over the short-term (12 weeks), and moderate certainty of a moderate effect (SMD −0.54, 95% CI, −1.0 to −0.08) over long-term (≥ 12 weeks) treatment. The estimate based on 6 nonrandomized studies was similar with high certainty.

##### Myoglobin (17 kDa)

Three randomized studies found that MCO dialysis results in a large increase in myoglobin removal with SMD 2.9 (95% CI, 1.31 to 4.49; high certainty). The reduction ratio for myoglobin was 30.3% higher with MCO dialysis (95% CI, 15.5 to 45.0; high certainty) with an estimate based on 5 randomized studies. We found similar results (high certainty) with 6 nonrandomized studies. Medium cut-off dialysis reduced predialysis myoglobin moderately with SMD −0.51 SD (95% CI −0.85 to −0.16; moderate certainty) based on 2 randomized studies.

##### Kappa free light chains (22.5 kDa)

Medium cut-off dialysis had a large effect on kappa free light chains (κ-FLC) removal with SMD 3.89 (95% CI, 3.45 to 4.33; high certainty), based on 2 randomized studies. The reduction ratio for κ-FLC was similarly increased with MD 14.9% (95% CI, 8.3 to 21.4; high certainty); results were similar in nonrandomized studies. Predialysis κ-FLC decreased by SMD −0.39 (95% CI, −0.61 to −0.16; high certainty) with MCO dialysis based on 5 randomized studies, with similar results from nonrandomized studies. In the subgroup with long-term treatment (≥24 weeks), MCO dialysis had a larger effect with SMD −0.49 (95% CI, −0.79 to −0.20; *I*^2^ = 24%).

##### Lambda free light chains (45 kDa)

Removal of lambda free light chain (λ-FLC) was increased by MCO dialysis with SMD 2.16 (95% CI, 1.8 to 2.52; high certainty), based on 3 randomized studies. The pooled estimate for the λ-FLC reduction ratio was 20.9% higher with MCO dialysis (95% CI, 15.5 to 26.2; high certainty), based on 7 randomized studies with similar results in nonrandomized studies. We found that MCO dialysis reduces predialysis λ-FLC moderately with SMD −0.53 (95% CI, −0.9 to −0.17; high certainty) with similar results from nonrandomized studies.

#### Cytokines and inflammatory markers

##### Tumor necrosis factor-alpha (17 kDa)

A single randomized study reported an MD in the reduction ratio for tumor necrosis factor-alpha (TNF-α) of 7.7% (95% CI, 4.7 to 10.6; moderate certainty), and 3 randomized studies measured a predialysis TNF-α decrease of SMD −0.48 (95% CI, −0.91 to −0.04; moderate certainty) with MCO dialysis. Expression of TNF-α mRNA in peripheral white blood cells decreased by MD −15% (relative quantitation) with MCO dialysis (95% CI, −19.6 to −10.4; moderate certainty) in a single randomized study that reported this outcome.

##### Interleukin-6 (24.5 kDa)

One randomized study measured the reduction ratio for interleukin-6 (IL-6) and found little to no difference with MCO dialysis with MD −0.2% (95% CI, −3.4 to 3.0; moderate certainty). The pooled estimate for predialysis IL-6 based on 4 randomized studies showed little to no difference with MCO dialysis with SMD 0.04 (95% CI, −0.17 to 0.25; moderate certainty). A single randomized study that measured peripheral leukocyte IL-6 mRNA expression found an MD of −8.8% (95% CI, −10.2 to −7.4; moderate certainty).

##### C-reactive protein

Two randomized and 5 nonrandomized studies provided moderate and high certainty, respectively, of little to no effect on C-reactive protein.

#### Additional analyses

Correlations (Pearson’s *r*) between treatment effects and molecular weights were (95% CI) −0.10 (−0.78 to 0.37) for removal, 0.24 (0.06 to 0.62) for reduction ratio, and −0.55 (−0.61 to −0.27) for predialysis serum concentrations for the 4 included middle molecules.

### Risk of Bias Across Studies

For the outcomes with 7 or more studies (predialysis serum albumin, albumin loss), we found no evidence of publication bias based on funnel plots.

## Discussion

### Principal Findings

We found with high certainty that MCO dialysis removes approximately 2 g of albumin per 4-hour conventional hemodialysis session, resulting in a decreased serum albumin level of 0.12 g/dl over the short term (<24 weeks), and returning to baseline thereafter. We found with moderate to high certainty, that compared with high-flux membranes, MCO membranes increase middle-molecule clearance as measured by direct dialysate quantitation and reduction ratios, leading to reduced predialysis serum concentrations of β2-microglobulin, κ-FLC, λ-FLC, and myoglobin—solutes representing the full spectrum of large middle molecular weights. We found little to no effect on IL-6 removal or predialysis levels, while IL-6 mRNA expression was reduced by 8.8% in peripheral leukocytes. Medium cut-off dialysis increased the reduction ratio of TNF-α with a moderate reduction in predialysis levels and reduced peripheral leukocyte mRNA expression by 15%. Collectively, these findings are consistent with the anticipated effects of the MCO membrane, and may account for improved clinical outcomes including reduced symptom burden, recovery time, infection, hospital length of stay, and quality of life described in our separate report of clinical outcomes.^3^

### Strengths and Limitations of this Review

To our knowledge, this is the first systematic review and meta-analysis comparing MCO with high-flux membranes. Strengths of this review include adherence to a rigorous registered protocol, a sensitive search strategy, performing study procedures in duplicate, and the use of GRADE methods.

The lack of validation of the included biomarkers as surrogate outcomes is a major limitation in this review. Although associated with important physiological processes and clinical outcomes, none of the included biomarkers meet regulatory or statistical criteria for surrogacy.^46,47^ A valid surrogate must not only exhibit a high degree of correlation with a “true” or clinical outcome but also reliably predict the true treatment effect in trials that included both outcomes. Therefore, despite their familiarity and frequent use in dialysis trials and guidelines, we caution against the sole use of biomarkers in clinical or other decision-making. Further limitations of this review include the exclusion of small solutes and lack of direct comparisons with convective therapies.

### Comparisons With Previous Research

Albumin is a biomarker with a strong association with dialysis outcomes. Although albumin is affected by the same upstream inflammatory processes that contribute to malnutrition and accelerated cardiovascular disease, it is not in the causal pathway for death or cardiovascular events.^48^ Nevertheless, albumin removal has been considered a safety outcome in evaluating the effects of MCO dialysis, and the albumin loss associated with high cut-off membranes (up to 11 g/hemodialysis) has been cause for concern, prompting fresh frozen plasma or albumin infusion.^49^ Similar complications have arisen from high-volume postdilution hemodiafiltration (HDF).^50^ Our most certain estimate found that MCO dialysis removed 2.31 g of albumin per hemodialysis, with a transient decrease in predialysis albumin of 0.12 g/dl (95% CI, −0.16 to −0.07) within 24 weeks of treatment, returning to baseline thereafter. In all the included studies, albumin levels remained within the reference range, and there were no reports of albumin depletion leading to discontinuation of MCO dialysis or the need for albumin infusion. Albumin removal of 5 to 15 g/day with peritoneal dialysis^51,52^ and 3 to 5 g/day with HDF has not to date been identified as harmful, possibly because the theoretical harms of albumin removal by dialysis are offset by the amelioration of the uremic milieu. The inverse relationship between albumin synthesis and cytokine expression would support this logic.^53^ The reduced IL-6 and TNF-α expression afforded by MCO dialysis could account for the apparent compensatory response that restores serum albumin to baseline values over the longer term. A similar phenomenon was observed in a post hoc analysis of the CONTRAST study, which found similar long-term albumin levels in patients treated with HDF versus low-flux hemodialysis, despite markedly higher albumin removal with HDF.^54^ The effect of HDF on albumin levels was associated with relatively lower IL-6 concentrations in the CONTRAST study as well.

Serum concentrations of middle molecules and inflammatory mediators are typically several-fold higher in end-stage renal disease compared with healthy individuals. While TNF-α is 4- to 5-fold higher, κ-FLC can be anywhere from 2- to 16-fold higher in uremia.^55^ We found that MCO dialysis provided greater removal of β2-microglobulin, myoglobin, λ-, and κ-FLC, that translated into lower predialysis concentrations in all these solutes, especially over the longer term. Although these treatment effects were moderate to large (based on SMD values of >0.5 and >0.8, respectively), they did not result in normalization of predialysis solute concentrations.^55^ Nevertheless, these selected middle molecules are associated with important pathophysiological processes, including left ventricular hypertrophy (TNF-α), coronary artery disease (IL-6), impaired immunity and increased infection risk (FLCs), malnutrition and cachexia (IL-6), and several others, and reducing the overall burden of these and other uremic wastes could potentially translate into clinically meaningful effects.

Although we did not include other biomarkers in this review, we would expect similar effects on other large middle molecules. The included studies indeed reported enhanced removal of fibroblast growth factor-23,^17^ prolactin,^17,21^ alpha1-microglobulin,^19-21^ complement factor D,^19^ and YKL-40^19^ with MCO dialysis. We also identified studies reporting other effects with MCO dialysis, including reduced advanced glycosylation end-product accumulation in human skin,^56^ increased vancomycin clearance,^57^ reduced in vitro vascular smooth muscle cell necrosis,^58^ as well as improved endothelial function and increased expression of antioxidants.^59^ Hence, whether mediated through direct effects on toxic solute levels, or through indirect effects such as downregulating cytokine expression, the net effects of the MCO dialysis dialyzer could translate into meaningful clinical effects. In our corresponding report of clinical outcomes, we found with moderate to high certainty that patients treated with MCO dialysis had lower rates of infection, higher quality of life scores, less fatigue, lower symptom burden, and reduced erythropoietin resistance and iron use, all of which are consistent with the enhanced large middle-molecule clearance described here.^3^

We found no correlation between molecular weight and removal or reduction ratios, suggesting that MCO dialysis had comparable effects on all selected middle molecules ranging from 11.8 to 45 kDa. The finding of comparable effects on molecules of widely variable size is not fully explained by the MCO membrane’s steep sieving curve which has a molecule weight retention onset value (the point at which the sieving coefficient drops below 0.9) at around 12 kDa, that is, that of β2-microglobulin. Above that threshold, we would expect relatively lower diffusive clearances with increasing molecular weight. In vitro and modeling studies have estimated that owing to its large pore sizes and narrow fiber diameter, the MCO membrane provides approximately 7-8000 ml of convective clearance through internal filtration, which likely accounts for most of the augmented large middle-molecule clearance, compared with high-flux membranes,^60^ and explains the uniformity of effect across the large middle-molecule spectrum. Interestingly, we found a moderately large inverse correlation between molecular weight and the effect on predialysis solute concentrations. This apparent dissociation between removal and serum solute concentrations suggests that MCO dialysis could exert indirect effects as suggested by the downregulation of IL-6 and TNF-α expression in peripheral leukocytes, and by the compensatory increase in albumin synthesis that occurs over the long term.

### Certainty of the Evidence

Certainty was generally moderate to high across measures. Given the relatively objective measurement of biomarkers, the only major source of risk of bias was patient attrition. Patients with attrition events may have had poorer health and might have systematically different results compared with those who did not. We also found statistical heterogeneity in several estimates, primarily because they varied from large to very large treatment effects, and this did not warrant downgrading. Heterogeneity was explained by subgroup analyses. Studies with low baseline (control group) reduction ratios had larger effect sizes. Predialysis middle-molecule concentrations were generally unaffected over the short term, but significantly reduced over the longer term. Removal was higher when measured as blood-side clearance versus dialysate quantitation. Interestingly, the dissociation between these measures suggests that some plasma clearance could be due to membrane adsorption to the MCO membrane. Other sources of heterogeneity might have included differences in dialysis treatment parameters, membrane surface area, and patients’ residual renal function. Although many of the studies were relatively small, most used crossover or before-after designs, which reduced variance due to case-mix (patients served as their own controls), enabling paired analysis design and improving power and precision. In crossover studies, reduced solute concentrations following treatment with MCO dialysis could have carried over into the high-flux period; however, this carryover effect would have biased all results toward the null, and therefore did not warrant downgrading. Finally, we did not downgrade any outcomes for indirectness as would be required if they were to be used as surrogates for clinical outcomes in a guideline.

### Implications for Decision Makers

This report is intended to provide complementary and contextual information for interpreting clinical effects of MCO dialysis and is not intended to directly inform decision-making. However, albumin has been considered a safety outcome by some authorities (eg, US Food and Drug Administration). While we found a statistically significant short-term effect on albumin removal with MCO dialysis, albumin loss with MCO dialysis appears to be transient and comparable in magnitude with other dialysis modalities.

## Conclusions

Compared with high-flux membranes, MCO dialyzers increase the elimination of large middle molecules, resulting in decreased predialysis solute concentrations of solutes ranging between 11.8 and 45 kDa. Although MCO dialysis did not normalize serum concentrations of these solutes, the net effect of enhanced clearance within the large middle-molecule spectrum could explain the range of beneficial clinical effects reported to date. Further study will help to establish causal relationships between key biomarkers and clinical outcomes. Future studies evaluating the comparative effects of MCO hemodialysis and convective therapies are sure to generate interest as well.

## Supplemental Material

sj-docx-1-cjk-10.1177_20543581211067090 – Supplemental material for Effects of Medium Cut-Off Versus High-Flux Hemodialysis Membranes on Biomarkers: A Systematic Review and Meta-Analysis Click here for additional data file. Supplemental material, sj-docx-1-cjk-10.1177_20543581211067090 for Effects of Medium Cut-Off Versus High-Flux Hemodialysis Membranes on Biomarkers: A Systematic Review and Meta-Analysis by Maryam Kandi, Romina Brignardello-Petersen, Rachel Couban, Celina Wu and Gihad Nesrallah in Canadian Journal of Kidney Health and Disease

sj-docx-2-cjk-10.1177_20543581211067090 – Supplemental material for Effects of Medium Cut-Off Versus High-Flux Hemodialysis Membranes on Biomarkers: A Systematic Review and Meta-Analysis Click here for additional data file. Supplemental material, sj-docx-2-cjk-10.1177_20543581211067090 for Effects of Medium Cut-Off Versus High-Flux Hemodialysis Membranes on Biomarkers: A Systematic Review and Meta-Analysis by Maryam Kandi, Romina Brignardello-Petersen, Rachel Couban, Celina Wu and Gihad Nesrallah in Canadian Journal of Kidney Health and Disease

sj-docx-3-cjk-10.1177_20543581211067090 – Supplemental material for Effects of Medium Cut-Off Versus High-Flux Hemodialysis Membranes on Biomarkers: A Systematic Review and Meta-Analysis Click here for additional data file. Supplemental material, sj-docx-3-cjk-10.1177_20543581211067090 for Effects of Medium Cut-Off Versus High-Flux Hemodialysis Membranes on Biomarkers: A Systematic Review and Meta-Analysis by Maryam Kandi, Romina Brignardello-Petersen, Rachel Couban, Celina Wu and Gihad Nesrallah in Canadian Journal of Kidney Health and Disease

sj-docx-4-cjk-10.1177_20543581211067090 – Supplemental material for Effects of Medium Cut-Off Versus High-Flux Hemodialysis Membranes on Biomarkers: A Systematic Review and Meta-Analysis Click here for additional data file. Supplemental material, sj-docx-4-cjk-10.1177_20543581211067090 for Effects of Medium Cut-Off Versus High-Flux Hemodialysis Membranes on Biomarkers: A Systematic Review and Meta-Analysis by Maryam Kandi, Romina Brignardello-Petersen, Rachel Couban, Celina Wu and Gihad Nesrallah in Canadian Journal of Kidney Health and Disease

sj-pptx-5-cjk-10.1177_20543581211067090 – Supplemental material for Effects of Medium Cut-Off Versus High-Flux Hemodialysis Membranes on Biomarkers: A Systematic Review and Meta-Analysis Click here for additional data file. Supplemental material, sj-pptx-5-cjk-10.1177_20543581211067090 for Effects of Medium Cut-Off Versus High-Flux Hemodialysis Membranes on Biomarkers: A Systematic Review and Meta-Analysis by Maryam Kandi, Romina Brignardello-Petersen, Rachel Couban, Celina Wu and Gihad Nesrallah in Canadian Journal of Kidney Health and Disease

sj-xlsx-6-cjk-10.1177_20543581211067090 – Supplemental material for Effects of Medium Cut-Off Versus High-Flux Hemodialysis Membranes on Biomarkers: A Systematic Review and Meta-Analysis Click here for additional data file. Supplemental material, sj-xlsx-6-cjk-10.1177_20543581211067090 for Effects of Medium Cut-Off Versus High-Flux Hemodialysis Membranes on Biomarkers: A Systematic Review and Meta-Analysis by Maryam Kandi, Romina Brignardello-Petersen, Rachel Couban, Celina Wu and Gihad Nesrallah in Canadian Journal of Kidney Health and Disease

sj-docx-7-cjk-10.1177_20543581211067090 – Supplemental material for Effects of Medium Cut-Off Versus High-Flux Hemodialysis Membranes on Biomarkers: A Systematic Review and Meta-Analysis Click here for additional data file. Supplemental material, sj-docx-7-cjk-10.1177_20543581211067090 for Effects of Medium Cut-Off Versus High-Flux Hemodialysis Membranes on Biomarkers: A Systematic Review and Meta-Analysis by Maryam Kandi, Romina Brignardello-Petersen, Rachel Couban, Celina Wu and Gihad Nesrallah in Canadian Journal of Kidney Health and Disease
