# Supplementary material for: Effects of Medium Cut-Off Versus High-Flux Hemodialysis Membranes on Biomarkers: A Systematic Review and Meta-Analysis
Source: Can J Kidney Health Dis. 2022 Jan 18;9:20543581211067090. doi: 10.1177/20543581211067090 (PMC8777328; doi:10.1177/20543581211067090)
Supplement: sj-docx-1-cjk-10.1177_20543581211067090 – Supplemental material for Effects of Medium Cut-Off Versus High-Flux Hemodialysis Membranes on Biomarkers: A Systematic Review and Meta-Analysis [file sj-docx-1-cjk-10.1177_20543581211067090.docx]

APPENDIX A - EFFECTIVENESS OF MEDIUM CUT-OFF VERSUS HIGH-FLUX HEMODIALYSIS MEMBRANES: A SYSTEMATIC REVIEW AND META-ANALYSIS

ADMINISTRATIVE INFORMATION

Title

Effectiveness of medium cut-off (MCO) versus high-flux hemodialysis membranes: a systematic review and meta-analysis

Version and Update Status

Version 1.0; *de novo* systematic review and meta-analysis

Authors

Study Sponsor: Dr. Angelito Bernardo, Baxter Healthcare

Principal Investigator: Dr. Gihad Nesrallah, Associate Scientist, Li Ka Shing Knowledge Institute

Co-investigators: Ms. Maryam Kandi, PhD Candidate, McMaster University

Dr. Romina Brignardello Petersen, PhD, McMaster University

Ms. Rachel Couban, MA, MISt, McMaster University

Ms. Celina Wu, MSc, BScPA, CCPA, University of Toronto

Primary Contact: Dr. Gihad Nesrallah

[gnesrallah@hrh.ca](mailto:gnesrallah@hrh.ca)

Mobile: +1-416-918-2730

SUPPORT

Funding Sources

Unrestricted research grant from Baxter Healthcare International

Role of Sponsor or Funder

Provided access to a library of published studies and conference abstracts (cross-referenced for validation of our primary search).

## AMMENDMENTS

1. Given the large number of outcomes identified in the included studies, we determined that reporting should be split into two separate manuscripts – one focusing on the pre-specificized ‘primary’ (clinical or patient-important) outcomes, and another addressing laboratory-based measures.
2. We encountered one RS with large baseline differences in outcomes that did not provide test statistics for change^1^; we therefore imputed the standard error for change using standard techniques^2^.
3. In assessing risk of bias across studies, we did not rate down for open-label design and provide a rationale for this approach in the Discussion.
4. Where primary intention-to-treat analyses were impacted by patient attrition, we verified their results with a per-protocol analysis. We did not downgrade for risk of bias due to attrition if the results were similar.
5. During the conduct of this systematic review, six studies that were initially identified as abstracts or preprints were subsequently published as full texts in peer reviewed journals. We therefore extracted these reports and updated our analyses, risk of bias assessments, certainty ratings, and the manuscript accordingly^3-8^.

INTRODUCTION

Rationale

Outcomes for patients on hemodialysis remain suboptimal, in part due to the limited clearance and persistence of various uremic toxins including larger solutes and middle molecules with high-flux dialyzers that remain the standard of care. Hemofiltration and hemodiafiltration can ameliorate middle molecule clearance but remain cumbersome and costly therapies that have not been scalable in high-income countries.

A new class of dialyzer — medium cut-off (MCO) or high-retention-onset (HRO) membranes — are characterized by larger pores with a narrow size distribution, that allow for enhanced clearance of middle molecules while retaining selectivity to exclude albumin and other larger proteins. Preliminary studies suggest improved clearance of several uremic substances including inflammatory mediators with other studies suggesting improved patient symptom burden and well-being.

Objectives

This systematic review and meta-analysis will evaluate the comparative effects of hemodialysis with MCO versus high-flux membranes, examining both patient-important efficacy and safety outcomes as well as physiological measures.

METHODS

Eligibility criteria

Study inclusion criteria

**P**opulation - adults (>18 years) with end-stage renal disease (ESRD)

**I**ntervention - hemodialysis with an MCO membrane

**C**omparator - hemodialysis with a standard high-flux dialysis membrane

**O**utcomes – see below

Eligible Study Designs

Randomized trials including crossover designs

Nonrandomized studies of interventions (NRSI) including crossover designs and cohort studies

Systematic reviews

Other Study Characteristics

No restriction on language or date of publication

Published in peer-reviewed journals

Conference abstracts for major nephrology conferences from 2017 or later

Study Exclusion Criteria

Studies of high cut-off, ‘super high-flux’, and other novel non-MCO membranes

Studies of MCO membranes in which the only comparator is hemofiltration / hemodiafiltration

Studies containing no original data such as editorials, narrative reviews, opinion pieces etc.

Information sources

We will search EMBASE, MEDLINE, CINAHL, CDSR and ISI Web of Science from Jan 1, 2015 to present. We will search reference lists of included studies and systematic reviews. We will hand search conference abstracts from major nephrology conferences including the Canadian Society of Nephrology, the National Kidney Foundation, the American Society of Nephrology, the European Renal Association/EDTA, and the World Congress of Nephrology to 2016.

Search Strategy

Terms

MCO-HD

MCO

Medium cut-off

Mid cut-off, mid cut off, mid cutoff

expanded hemodialysis

HDx

[medium cut-off OR MCO] AND [dialy* (dialyzer, dialysis) OR membrane]

High-Retention-Onset Membranes

Study records

Data management

Citations will be exported to EndNote 9.3, combined, and de-duplicated, then exported to DistillerSR systematic review software for screening and data abstraction.

Selection Process

We will develop title and abstract and full-text screening forms and pilot test them until kappa > 85% is achieved, with 2 reviewers. Screening will proceed in duplicate with disagreements resolved through discussion.

We will leverage artificial intelligence-based tools (the DistillerSR Artificial Intelligence System (DAISY) v. 2.0; <https://blog.evidencepartners.com/whats-new-in-distillersr-next-level-automation>) for screening titles and abstracts and will supplement these with verification by a single reviewer.

Data Collection Process

Data will be extracted into pilot-tested forms by a single reviewer with verification/quality control by a second reviewer.

Data Items

Data extraction forms will capture relevant variables across the following categories: methods (design, setting) , participant characteristics (demographics, eligibility criteria), characteristics of interventions, risk of bias evaluation criteria, patient disposition, and outcomes (including counts, rates, measures of central tendency and dispersion, and statistical significance).

Outcomes and Prioritization

Primary Outcomes

Efficacy

Death

Hospitalization

QoL

Any symptom-related PRO

Recovery time

Safety

Access thrombosis

Extracorporeal circuit thrombosis

Serum albumin

Infection

Deficiency states (as defined in the studies)

Secondary Outcomes

Erythropoietin requirements

Laboratory-based measures (absolute and relative reductions or similar measures):

- other middle molecules (β_2_-microglobulin, myoglobin, lambda and kappa free light chains)
- inflammatory mediators (IL-6, TNF-alpha)
- C-reactive protein
- protein-bound solutes.

Risk of bias in individual studies

We will use the Cochrane RoB tool version 2 for randomized studies (<https://www.riskofbias.info/welcome/rob-2-0-tool>) and the ROBINS-I tool for nonrandomized studies (<https://www.riskofbias.info/welcome/home>)

Data synthesis

We will perform meta-analysis using the Cochrane RevMan platform v 5.3. We will combine RCT and nonrandomized study data separately using standard meta-analysis techniques including generic inverse variance for rate data (expressed as rate ratios), and weighted or SMDs as appropriate for continuous data. We will use random-effects models. For continuous data, we will use change scores where sufficient information is available and will otherwise use final values.

We will aim to use study participants as the unit of analysis. However, given the relatively high preponderance of crossover studies with unpaired analyses in the dialysis literature, we anticipate that we will need to either need to use imputation techniques to calculate SEs that reflect paired analysis design or else revert to naïve analysis (and accept some loss of precision) where required.

We will quantify heterogeneity using the I^2^ statistic and will explore heterogeneity through subgroup analysis with prespecified subgroups defined by study duration (short versus long), publication type (peer reviewed versus conference abstracts), funding source, and based on other study design characteristics such as availability of change scores versus final values and presence versus absence of blinding of participants providing patient-reported outcome data such as symptom scores and QoL.

We will use qualitative synthesis methods to summarize relevant results that are not amenable to meta-analysis and will present these in tables where appropriate.

Meta-bias(es)

We will use funnel plots to assess the risk of publication bias.

Confidence in Cumulative Evidence

We will apply the GRADE appraisal criteria to assess the quality of evidence within and across studies and summarize these findings in standard GRADE Summary of Findings Tables, using the GRADEpro software platform in the GRADE Guideline Development Tool (GDT; <https://gradepro.org>).

## REFERENCES

1. Lim JH, Park Y, Yook JM, et al. Randomized controlled trial of medium cut-off versus high-flux dialyzers on quality of life outcomes in maintenance hemodialysis patients. *Sci* 2020; **10**(1): 7780.

2. 6.5.2.8 Imputing standard deviations for changes from baseline. In: Higgins J, Thomas J, Chandler J, et al., eds. Cochrane Handbook for Systematic Reviews of Interventions version 62: Cochrane; 2021.

3. Alarcon JC, Bunch A, Ardila F, et al. Impact of Medium Cut-Off Dialyzers on Patient-Reported Outcomes: COREXH Registry. *Blood Purification* 2021; **50**(1): 110-8.

4. Ariza JG, Walton SM, Suarez AM, Sanabria M, Vesga JI. An initial evaluation of expanded hemodialysis on hospitalizations, drug utilization, costs, and patient utility in Colombia. *Therapeutic Apheresis and Dialysis* 2021.

5. Bunch A, Sanchez R, Nilsson LG, et al. Medium cut‐off dialyzers in a large population of hemodialysis patients in Colombia: COREXH registry. *Therapeutic Apheresis and Dialysis* 2021; **25**(1): 33-43.

6. Cozzolino M, Magagnoli L, Ciceri P, Conte F, Galassi A. Effects of a medium cut-off (Theranova®) dialyser on haemodialysis patients: a prospective, cross-over study. *Clin Kidney J* 2021; **14**(1): 382-9.

7. Lim J-H, Jeon Y, Yook J-M, et al. Medium cut-off dialyzer improves erythropoiesis stimulating agent resistance in a hepcidin-independent manner in maintenance hemodialysis patients: results from a randomized controlled trial. *Sci* 2020; **10**(1).

8. Weiner DE, Falzon L, Skoufos L, et al. Efficacy and Safety of Expanded Hemodialysis with the Theranova 400 Dialyzer. *Clinical Journal of the American Society of Nephrology* 2020; **15**(9): 1310-9.
