# Supplementary material for: Effects of Medium Cut-Off Versus High-Flux Hemodialysis Membranes on Biomarkers: A Systematic Review and Meta-Analysis
Source: Can J Kidney Health Dis. 2022 Jan 18;9:20543581211067090. doi: 10.1177/20543581211067090 (PMC8777328; doi:10.1177/20543581211067090)
Supplement: sj-docx-2-cjk-10.1177_20543581211067090 – Supplemental material for Effects of Medium Cut-Off Versus High-Flux Hemodialysis Membranes on Biomarkers: A Systematic Review and Meta-Analysis [file sj-docx-2-cjk-10.1177_20543581211067090.docx]

# Appendix B – Detailed Methods

Protocol and registration

We registered our protocol (Appendix A) with the Prospero Register of Systematic Reviews (registration number CRD42020204636). We prepared this manuscript in accordance with the PRISMA guideline.(1) We present abbreviated methods in the main manuscript and provide further details in this Appendix.

Eligibility criteria

Types of studies

We sought to include randomized studies (parallel arm and crossover), nonrandomized studies of interventions including cohort studies and before-after designs, as well as systematic reviews. We placed no restrictions on language. We limited the search timeframe to begin in 2015 to coincide with the first-ever published reports describing the intervention.

Types of participants

We included studies that enrolled adult patients (>18 years) with end-stage renal disease (ESRD) receiving maintenance hemodialysis. We excluded patients with acute kidney injury and those admitted to hospital.

Types of interventions

The only eligible intervention was a novel medium cutoff polyarylethersulfone /polyvinylpyrrolidone membrane with narrow pore size distribution (Theranova 400/500, Baxter Healthcare, Deerfield, IL, USA) and related prototypes. We excluded studies of high cutoff and “super high-flux” membranes. Eligible comparators were limited to high-flux membranes used in hemodialysis applications; we excluded studies in which the only comparators were hemofiltration/hemodiafiltration.

Types of outcomes

Primary efficacy outcomes included mortality, hospitalization, quality of life, symptoms, and other patient-reported outcome measures. Primary safety outcomes focused on hypothetical deficiency states resulting from nonselective protein and other large molecule removal potentially leading to impaired immunity or hypercoagulability. These included extracorporeal circuit and access thrombosis, infection, and albumin depletion.

Secondary/physiological outcomes included laboratory-based measures of β-2-microglobulin, myoglobin, λ- and κ-free light chains, IL-6, TNF-α, C-reactive protein, and protein-bound solutes. Where available, we included predialysis levels (reflecting the post-rebound steady state), reduction ratios, and clearance, or removal as measured through direct dialysate quantitation.

Information sources

We ran our primary search strategy in five databases (MEDLINE, EMBASE, CINAHL, Cochrane Library, and Web of Science), without restricting by language or study design, limiting by date to capture results since 2015. Grey literature sources included a database of relevant articles compiled by the manufacturer, which included a comprehensive listing of conference abstract, as well as full-text manuscripts reviewed and accepted for publication in peer-reviewed journals. We cross-referenced our primary search against this database for added sensitivity.

Search

Our search strategy included two concepts – hemodialysis and medium cut-off membranes. Synonymous terms for each concept were combined using the OR operator; two concepts were combined using the AND operator. The search strategy is in Appendix C

Study selection

We imported citations into EndNote 9.3 for de-duplication then uploaded them to the DistillerSR online systematic review software platform for screening. For primary search results, we used pilot-tested title and abstract and full-text screening forms and screened all reports in duplicate resolving conflicts through discussion. Grey literature sources were screened by one reviewer. Where we identified multiple reports of the same study population, we use the most recent, comprehensive, or peer-reviewed report for data extraction.

Data collection process

Each reviewer extracted data independently into standard forms with independent verification by second reviewer.

Data items

We extracted key variables across the following categories: methods (design, setting), participant characteristics (demographics, eligibility criteria), characteristics of interventions, risk of bias evaluation criteria, patient disposition, and outcomes (including counts, rates, measures of central tendency and dispersion, and statistical significance).

When studies reported measures at multiple time points, we used the last available value (representing the longest possible follow-up) for meta-analysis. We extracted counts of patients with one or more hospitalization events rather than total number of hospitalizations per group to avoid double counting. For studies reporting death or hospitalization, we only considered those with a minimum of 10 weeks of follow-up for meta-analysis, excluding those with shorter follow-up from pooled estimates for these outcomes.

Studies that reported the reduction ratio (RR) for any solute with concentration C used the following formula:

The final post-dialysis solute concentration (C*_post_*) was corrected for the degree of hemoconcentration and the volume of distribution (approximate extracellular volume) according to Bergström and Wehle:(2)

To give C*_post-corr_*, where BW represents body weight pre- and post-dialysis.

Risk of bias in individual studies

We used the Cochrane RoB tool version 2 for randomized studies (<https://www.riskofbias.info/welcome/rob-2-0-tool>) and the ROBINS-I tool for non-randomized studies (<https://www.riskofbias.info/welcome/home>). Two reviewers assessed risk of bias independently and discussed any conflicts until consensus was reached.

Summary measures

For continuous variables, we extracted change scores and their corresponding standard errors (SEs) or *P* values, where available, and used *P* values to impute the SE for change where required, using the following formula:

$$SE=\sqrt{\frac{\left( N_{1}-1 \right)S_{1}^{2}+(N_{2}-1)S_{2}^{2}}{N_{1}+N_{2}-2})(\frac{1}{N_{1}}+\frac{1}{N_{2}})}$$

Where this was infeasible, we collected final values and their corresponding measures of dispersion and planned to meta-analyze change scores and final values as subgroups and pool these estimates if appropriate.

We planned to use the patient as the unit of analysis. For randomized crossover trials, we extracted treatment effect estimates reported as paired analyses where possible.

When studies did not report a standard deviation for given value, we imputed it using the mean coefficient of variation from the other studies reporting a mean and SD for that measure.

For outcomes that were reported using different units of measurement (*e.g.,* erythropoiesis resistance index and C-reactive protein), we calculated the standardized mean difference (SMD) and related SE estimate, using the following formulae (3):

$$SMD=\frac{X_{1}-X_{2}}{S_{pooled}}$$

$$S_{pooled}=\sqrt{\frac{\left( N_{1}-1 \right)S_{1}^{2}+(N_{2}-1)S_{2}^{2}}{N_{1}+N_{2}-2})}$$

$$\mathrm{SE}_{SMD}=\sqrt{\frac{N_{1}+N_{2}}{N_{1}N_{2}}+\frac{{SMD}^{2}}{N_{1}+N_{2}-2}}$$

For all equations:

$X_{1}$: Mean in high-flux HD group

$X_{2}$: Mean in MCO group

$N_{1}$: sample size in high-flux HD group

$N_{2}$: sample size in MCO group

$S_{1}^{2}$: variance in high-flux HD group

$S_{2}^{2}$: variance in MCO group

Synthesis of results

We used the generic inverse variance method to pool continuous data using mean differences or SMDs and corresponding SE estimates. For count data including mortality, hospitalization, and infection, we calculated total follow-up in each study arm (in patient-days) and calculated risk ratios and corresponding standard error estimates. We calculated odds ratios and corresponding SEs for dichotomous outcomes. In all instances, we planned to use random-effect models, reverting to fixed-effects models where only 2 studies were available for pooling and where a random-effects model resulted in inappropriately large weighting of a small study. We pooled randomized trials and observational studies separately. Where the quality of evidence/certainty was identical for randomized trials and observational studies for a given outcome, and where heterogeneity was undetected or low, we pooled effect estimates from both bodies of evidence. In most instances, this was done to improve precision where it would have otherwise been low with each estimate reported separately.

While extracting data, we realized that many patient-reported outcome measures were available for meta-analysis. In order to avoid any potential bias in grouping these outcomes (given that all members of the review team had seen some or all of the extracted data), we used the approach proposed by Johnston et al. and provided a list of available measures (instruments and subscales) to an independent collaborator to generate appropriate groupings of measures in a blinded manner (4).

We planned to use the *I*^2^ statistic to explore heterogeneity through subgroup analysis with prespecified subgroups defined by study duration (short versus long), publication type (peer-reviewed versus conference abstracts), funding source, and based on other study design characteristics, such as availability of change scores versus final values and presence versus absence of blinding of participants providing patient-reported outcome measures.

When studies met eligibility criteria for population, intervention, and comparator but did not report any prespecified outcomes, or were not amenable to meta-analysis, we extracted and summarized their key findings using standard narrative synthesis techniques.

Risk of bias across studies

Where possible, we used funnel plots to assess for publication bias.

Certainty Assessment

We assessed the quality of evidence on an outcome by outcome basis using the GRADE approach and summarized these assessments in GRADE Summary of Findings Tables using the GRADEpro/Guideline Development Tool (GDT: <https://gdt.gradepro.org/app/#projects>) (5-7). Evidence quality (and the certainty arising from it) was classified as very low, low, moderate, or high. Each effect estimate (from randomized controlled trials and observational studies) started with a “high” certainty rating and was rated down one or two levels depending on the degree of risk of bias (8, 9), inconsistency (10), indirectness (11), imprecision (12), or publication bias (13). Observational studies could be rated up for a large treatment effect, dose response gradient, or when all plausible residual confounding would further increase our confidence in the effect estimate (14).

For each outcome, we selected and summarized the effect estimates that were based on the body of evidence with the highest overall quality rating. When randomized trials and observational studies had the same quality ratings, we retained both effect estimates in the Summary of Findings Table for consideration by decision makers. The Summary of Findings Tables include both absolute and relative effect estimates. Given a relative treatment effect (*i.e.,* risk ratio) and event rate in the control group (*i.e.,* the baseline event rate) the GDT software platform will automatically calculate a corresponding anticipated absolute effect estimate for illustrative purposes for consideration by decision makers.

## REFERENCES

1. Liberati A, Altman DG, Tetzlaff J, Mulrow C, Gøtzsche PC, Ioannidis JPA, Clarke M, Devereaux PJ, Kleijnen J, Moher D: The PRISMA Statement for Reporting Systematic Reviews and Meta-Analyses of Studies That Evaluate Health Care Interventions: Explanation and Elaboration. *PLoS Medicine,* 6**:** e1000100, 2009 10.1371/journal.pmed.1000100

2. Bergström J, Wehle B: NO CHANGE IN CORRECTED β2-MICROGLOBULIN CONCENTRATION AFTER CUPROPHANE HAEMODIALYSIS. 329**:** 628-629, 1987 10.1016/s0140-6736(87)90266-2

3. Higgins JPT, Thomas J, Chandler J, Cumpston M, Li T, Page MJ, (editors) WV: *Cochrane Handbook for Systematic Reviews of Interventions version 6.0 (updated July 2019)*, Cochrane, 2019

4. Johnston BC, Patrick DL, Busse JW, Schunemann HJ, Agarwal A, Guyatt GH: Patient-reported outcomes in meta-analyses--Part 1: assessing risk of bias and combining outcomes. *Health Qual Life Outcomes,* 11**:** 109, 2013 10.1186/1477-7525-11-109

5. Guyatt G, Oxman AD, Sultan S, Brozek J, Glasziou P, Alonso-Coello P, Atkins D, Kunz R, Montori V, Jaeschke R, Rind D, Dahm P, Akl EA, Meerpohl J, Vist G, Berliner E, Norris S, Falck-Ytter Y, Schunemann HJ: GRADE guidelines: 11. Making an overall rating of confidence in effect estimates for a single outcome and for all outcomes. *J Clin Epidemiol,* 66**:** 151-157, 2013 10.1016/j.jclinepi.2012.01.006

6. Guyatt GH, Oxman AD, Santesso N, Helfand M, Vist G, Kunz R, Brozek J, Norris S, Meerpohl J, Djulbegovic B, Alonso-Coello P, Post PN, Busse JW, Glasziou P, Christensen R, Schunemann HJ: GRADE guidelines: 12. Preparing summary of findings tables-binary outcomes. *J Clin Epidemiol,* 66**:** 158-172, 2013 10.1016/j.jclinepi.2012.01.012

7. Guyatt GH, Thorlund K, Oxman AD, Walter SD, Patrick D, Furukawa TA, Johnston BC, Karanicolas P, Akl EA, Vist G, Kunz R, Brozek J, Kupper LL, Martin SL, Meerpohl JJ, Alonso-Coello P, Christensen R, Schunemann HJ: GRADE guidelines: 13. Preparing summary of findings tables and evidence profiles-continuous outcomes. *J Clin Epidemiol,* 66**:** 173-183, 2013 10.1016/j.jclinepi.2012.08.001

8. Balshem H, Helfand M, Schunemann HJ, Oxman AD, Kunz R, Brozek J, Vist GE, Falck-Ytter Y, Meerpohl J, Norris S, Guyatt GH: GRADE guidelines: 3. Rating the quality of evidence. *J Clin Epidemiol,* 64**:** 401-406, 2011 10.1016/j.jclinepi.2010.07.015

9. Guyatt GH, Oxman AD, Vist G, Kunz R, Brozek J, Alonso-Coello P, Montori V, Akl EA, Djulbegovic B, Falck-Ytter Y, Norris SL, Williams JW, Jr., Atkins D, Meerpohl J, Schunemann HJ: GRADE guidelines: 4. Rating the quality of evidence--study limitations (risk of bias). *J Clin Epidemiol,* 64**:** 407-415, 2011 10.1016/j.jclinepi.2010.07.017

10. Guyatt GH, Oxman AD, Kunz R, Woodcock J, Brozek J, Helfand M, Alonso-Coello P, Glasziou P, Jaeschke R, Akl EA, Norris S, Vist G, Dahm P, Shukla VK, Higgins J, Falck-Ytter Y, Schunemann HJ, Group GW: GRADE guidelines: 7. Rating the quality of evidence--inconsistency. *J Clin Epidemiol,* 64**:** 1294-1302, 2011 10.1016/j.jclinepi.2011.03.017

11. Guyatt GH, Oxman AD, Kunz R, Woodcock J, Brozek J, Helfand M, Alonso-Coello P, Falck-Ytter Y, Jaeschke R, Vist G, Akl EA, Post PN, Norris S, Meerpohl J, Shukla VK, Nasser M, Schunemann HJ, Group GW: GRADE guidelines: 8. Rating the quality of evidence--indirectness. *J Clin Epidemiol,* 64**:** 1303-1310, 2011 10.1016/j.jclinepi.2011.04.014

12. Guyatt GH, Oxman AD, Kunz R, Brozek J, Alonso-Coello P, Rind D, Devereaux PJ, Montori VM, Freyschuss B, Vist G, Jaeschke R, Williams JW, Jr., Murad MH, Sinclair D, Falck-Ytter Y, Meerpohl J, Whittington C, Thorlund K, Andrews J, Schunemann HJ: GRADE guidelines 6. Rating the quality of evidence--imprecision. *J Clin Epidemiol,* 64**:** 1283-1293, 2011 10.1016/j.jclinepi.2011.01.012

13. Guyatt GH, Oxman AD, Montori V, Vist G, Kunz R, Brozek J, Alonso-Coello P, Djulbegovic B, Atkins D, Falck-Ytter Y, Williams JW, Jr., Meerpohl J, Norris SL, Akl EA, Schunemann HJ: GRADE guidelines: 5. Rating the quality of evidence--publication bias. *J Clin Epidemiol,* 64**:** 1277-1282, 2011 10.1016/j.jclinepi.2011.01.011

14. Guyatt GH, Oxman AD, Sultan S, Glasziou P, Akl EA, Alonso-Coello P, Atkins D, Kunz R, Brozek J, Montori V, Jaeschke R, Rind D, Dahm P, Meerpohl J, Vist G, Berliner E, Norris S, Falck-Ytter Y, Murad MH, Schunemann HJ, Group GW: GRADE guidelines: 9. Rating up the quality of evidence. *J Clin Epidemiol,* 64**:** 1311-1316, 2011 10.1016/j.jclinepi.2011.06.004
