# Supplementary material for: Effects of Medium Cut-Off Versus High-Flux Hemodialysis Membranes on Biomarkers: A Systematic Review and Meta-Analysis
Source: Can J Kidney Health Dis. 2022 Jan 18;9:20543581211067090. doi: 10.1177/20543581211067090 (PMC8777328; doi:10.1177/20543581211067090)
Supplement: sj-docx-4-cjk-10.1177_20543581211067090 – Supplemental material for Effects of Medium Cut-Off Versus High-Flux Hemodialysis Membranes on Biomarkers: A Systematic Review and Meta-Analysis [file sj-docx-4-cjk-10.1177_20543581211067090.docx]

# Appendix D – Full Listing of Included Reports Grouped by Study

Primary references (full-text publication) are labelled with *

## Alarcon 2021

* Alarcon JC, Bunch A, Ardila F, Zuñiga E, Vesga JI, Rivera A, Sánchez R, Sanabria RM: Impact of Medium Cut-Off Dialyzers on Patient-Reported Outcomes: COREXH Registry. *Blood Purification,* 50**:** 110-118, 2021 10.1159/000508803

Sanabria M, Rivera AS, Bernardo AA, Nilsson LG, Vesga J, Bunch A, Sanchez R: Patient-Reported Outcome Measures (PROMs) and Expanded Hemodialysis (HDx) with Medium Cut-Off Dialyzers in a Large Cohort of Patients in Colombia: The COREXH Study. FRPO493. *J Am Soc Nephrol,* 30**:** 568, 2019

## Albrizio 2018

Albrizio P, Costa S, Foschi A, Milani IAA, Rindi S, Zucchi M, Milanesi F: New medium cut-off membrane vs online hemodiafiltration in clearance of middle molecules. preliminary results from our centre. *Nephrology Dialysis Transplantation,* 33 (Supplement 1)**:** i193, 2018 http://dx.doi.org/10.1093/ndt/gfy104.FP466

## Allawati 2020

Allawati H, Dallas L, Nair S, Palmer J, Thaikandy S, Hutchison C: A Pharmacokinetic Study Comparing the Clearance of Vancomycin during Haemodialysis Using Medium Cut-Off Membrane (Theranova) and High-Flux Membranes (Revaclear). *Toxins (Basel),* 12**:** 12, 2020 https://dx.doi.org/10.3390/toxins12050317

## Arizia 2020

*Ariza JG, Walton SM, Suarez AM, Sanabria M, Vesga JI: An initial evaluation of expanded hemodialysis on hospitalizations, drug utilization, costs, and patient utility in Colombia. *Therapeutic Apheresis and Dialysis*, 2021 10.1111/1744-9987.13620

Ariza JG, Walton SM, Sanabria M, Vesga J, Suarez A, Rivera A: Puk15 Measuring the Association of Switching Patients from Hemodialysis to Expanded Hemodialysis, with Hospitalizations, Medication Use, Costs, and Patient Utility. *Value in Health,* 23 (Supplement 1)**:** S379-S380, 2020 http://dx.doi.org/10.1016/j.jval.2020.04.1473

## Baharani 2017

Baharani J, Barrios B, Hopkins D, Passmore W: UK clinical experiences of a new expanded haemodialysis therapy with a novel medium cut-off dialyser [poster SA-PO760] Heartlands Hospital n=8, Morristone Hospital n=18. *J Amer Soc Nephrol* 28**:** 875, 2017

## Belmouaz 2020

*Belmouaz M, Bauwens M, Hauet T, Bossard V, Jamet P, Joly F, Chikhi E, Joffrion S, Gand E, Bridoux F: Comparison of the removal of uraemic toxins with medium cut-off and high-flux dialysers: a randomized clinical trial. *Nephrology Dialysis Transplantation,* 35**:** 328-335, 2020 <https://dx.doi.org/10.1093/ndt/gfz189>

Belmouaz M, Bauwens M, Hauet T, Bridoux F: Comparison of the removal of uremic toxins with medium cut-off and high-flux dialyzers: A randomized clinical trial. *Nephrology Dialysis Transplantation,* 34 (Supplement 1)**:** a222-a223, 2019 http://dx.doi.org/10.1093/ndt/gfz106.FP528

## Bolton 2020

Bolton S, Gair R, Nilsson L, Matthews M, Stewart L, McCullagh N: Dialysis recovery time and symptom burden in expanded hemodialysis (HDx). *Blood Purification (Under Review)*, 2020

## Bove 2018

Bove S, D'Avanzo L, Terlizzi V, Spitti C, Scolari F: Comparison of high flux hemodialyzers with medium cut-off dialyzer on the removal of middle high size uremic toxins. *Nephrology Dialysis Transplantation,* 33 (Supplement 1)**:** i190, 2018 <http://dx.doi.org/10.1093/ndt/gfy104.FP458>

## Bunch 2021

Bunch A, Nilsson LG, Vesga J, Ardila F, Zuniga E, Alarcon J, Suarez AM, Sanchez R, Sanabria M: Lomg term effects of expanded hemodialysis (HDx) on clinical and laboratory paramenters in a large cohort of dialysis patients [poster] FR-PO766. *J Am Soc Nephrol,* 29**:** 620, 2018

*Bunch A, Sanchez R, Nilsson LG, Bernardo AA, Vesga JI, Ardila F, Guerrero IM, Sanabria RM, Rivera AS: Medium cut‐off dialyzers in a large population of hemodialysis patients in Colombia: COREXH registry. *Therapeutic Apheresis and Dialysis,* 25**:** 33-43, 2021 10.1111/1744-9987.13506

## Cantaluppi 2018

Cantaluppi V, D’onati G, Lacquaniti A, Cosa F, Gernone G, Marengo M, Teatini U: Removal of large-middle molecules on expanded hemodialysis (HDx): a multicentric observational study of 6 month follow-up [Poster TH-PO357]. *J Am Soc Nephrol,* 29**:** 206, 2018

*Cantaluppi V, Marengo M, Alessandro Q, Berto M, Donati G, Antonio L, Cosa F, Gemone G, Teatini U, Migliori M, Panichi V: Removal of large-middle molecules, inhibition of neutrophil activation and modulation of inflammation-related endothelial dysfunction during expanded hemodialysis (HDX). *Nephrology Dialysis Transplantation,* 34 (Supplement 1)**:** a22, 2019 <http://dx.doi.org/10.1093/ndt/gfz096.FO048>

## Celik 2018

Celik L, Irlando A, Mortellaro RF, Castestetto E, Fornasiero G, Amici G: Plasma molecular changes in hemodialysis patients with polyarylethersulfone-polyvinylpyrrolidone membrane dialyzer (theranova 400 baxter). *Nephrology Dialysis Transplantation,* 33 (Supplement 1)**:** i184-i185, 2018 <http://dx.doi.org/10.1093/ndt/gfy104.FP444>

## Cho 2019

Cho NJ, Park S, Islam I, Lee EY, Gil HW: Long-term effect of medium cut-off dialyzer on middle uremic toxins and cell-free hemoglobin. *Nephrology Dialysis Transplantation,* 34 (Supplement 1)**:** a230, 2019 <http://dx.doi.org/10.1093/ndt/gfz106.FP552>

*Cho NJ, Park S, Islam MI, Song HY, Lee EY, Gil HW: Long-term effect of medium cut-off dialyzer on middle uremic toxins and cell-free hemoglobin. *PLoS ONE,* 14**:** e0220448, 2019 <https://dx.doi.org/10.1371/journal.pone.0220448>

## Ciceeri 2020

Ciceri P, Tettamanti G, Galassi A, Fabresse N, Alvarez JC, Massy ZA, Messa P, Cozzolino M: Pro-calcifying analysis of uremic serum from patients treated with medium cutoff membrane, in a prospective, cross-over study. *Nephrology Dialysis Transplantation,* [submitted], [2020]

## Cordeiro 2020

Cordeiro ISF, Cordeiro L, Wagner CS, Araujo L, Pereira BJ, Abensur H, Elias RM, Silva BC: High-Flux versus High-Retention-Onset Membranes: In vivo Small and Middle Molecules Kinetics in Convective Dialysis Modalities. *Blood Purification,* 49**:** 8-15, 2020 <https://dx.doi.org/10.1159/000502082>

## Cozzolino 2019

Cozzolino M, Magagnoli L, Ciceri P, Conte F, Galassi A: Medium cut-off (Theranova) dialyzer reduces the number of infections in hemodialysis patients: A prospective, cross-over study. *Nephrology Dialysis Transplantation,* 34 (Supplement 1)**:** a515, 2019 <http://dx.doi.org/10.1093/ndt/gfz103.SP464>

## D'Achiardi 2020

D'Achiardi R, Zuñiga E, Molano A, Tran H, Parreno R, Vesga J, Rivera A: P1063PERFORMANCE OF MEDIUM CUT-OFF DIALYZERS IN EXPANDED HEMODIALYSIS PATIENTS IN COLOMBIA. *Nephrology Dialysis Transplantation,* 35, 2020 10.1093/ndt/gfaa142.P1063

## Donadei 2019

Donadei C, Angeletti A, Gasperoni L, Guglielmo C, Donati G, La Manna G: Single hemodialysis (HD) treatment promotes T cells activation. *Nephrology Dialysis Transplantation,* 34 (Supplement 1)**:** a294, 2019 <http://dx.doi.org/10.1093/ndt/gfz106.FP722>

## Gallo 2019

Gallo M: The real-life study on expanded hemodialysis (HDX): 9 months experience of a single hemodialysis unit. *Nephrology Dialysis Transplantation,* 34 (Supplement 1)**:** a226, 2019 <http://dx.doi.org/10.1093/ndt/gfz106.FP539>

## Garcia-Prieto

*Garcia-Prieto A, Vega A, Linares T, Abad S, Macias N, Aragoncillo I, Torres E, Hernandez A, Barbieri D, Luno J: Evaluation of the efficacy of a medium cut-off dialyser and comparison with other high-flux dialysers in conventional haemodialysis and online haemodiafiltration. *Clin Kidney J,* 11**:** 742-746, 2018 <https://dx.doi.org/10.1093/ckj/sfy004>

Garcia-Prieto A, Vega A, Linares T, Abad S, MacIas N, Torres E, Hurtado E, Barbieri D, Luno J: Evaluation of the efficacy of a medium cut off dialyzer and comparison to other high flux dialyzers in conventional hemodialysis and online hemodiafiltration. *Nephrology Dialysis Transplantation,* 33 (Supplement 1)**:** i332-i333, 2018 <http://dx.doi.org/10.1093/ndt/gfy104.SaO041>

## Gernone 2020

Gernone G, Partipilo F, Detomaso F, Pepe V, Pietanza S, Mitrotti A: P1084LONG TERM EVALUATION OF THE EXPANDED HEMODIALYSIS (HDX) ON DIALYSIS ADEQUACY, ANEMIA AND QUALITY OF LIFE. *Nephrology Dialysis Transplantation,* 35, 2020 10.1093/ndt/gfaa142.P1084

## Jung 2019

*Jung JH, Ahn S-H, Song JH: p1098 The change of plasma sclerostin and other biomarkers in hemodialytic patients using dialyzer with medium cut-off membrane. *Nephrology Dialysis Transplantation,* 35, 2020 10.1093/ndt/gfaa142.P1098

Jung JH, Song JH, Ahn SH: A 6-month study on the efficacy of hemodialysis therapy using dialyzers with medium cut-off membranes in asian patients with end-stage renal disease. *Nephrology Dialysis Transplantation,* 34 (Supplement 1)**:** a522, 2019 <http://dx.doi.org/10.1093/ndt/gfz103.SP487>

## Kim 2019

*Kim TH, Kim SH, Kim TY, Park HY, Jung KS, Lee MH, Jhee JH, Lee JE, Choi HY, Park HC: Removal of large middle molecules via haemodialysis with medium cut-off membranes at lower blood flow rates: an observational prospective study. *BMC Nephrol,* 21**:** 2, 2019 <https://dx.doi.org/10.1186/s12882-019-1669-3>

Kim TH, Lee MH, Lee JE, Kim H, Choi HY, Park HC: Efficacy of medium cut-off dialyzer and comparison with standard high-flux hemodialysis and predilution online hemodiafiltration. *Nephrology Dialysis Transplantation,* 34 (Supplement 1)**:** a513, 2019 <http://dx.doi.org/10.1093/ndt/gfz103.SP458>

## Kirsch 2017

Kirsch AH, Lechner P, Nilsson LG, Beck W, Amdahl M, Krieter DH, Rosenkranz AR: Large middle-molecule removal during hemodialysis using a novel medium cutoff dialyzer. *Nephrology Dialysis Transplantation,* 31**:** i230, 2016 <http://dx.doi.org/10.1093/ndt/gfw170.23>

*Kirsch AH, Lyko R, Nilsson LG, Beck W, Amdahl M, Lechner P, Schneider A, Wanner C, Rosenkranz AR, Krieter DH: Performance of hemodialysis with novel medium cut-off dialyzers. *Nephrology Dialysis Transplantation,* 32**:** 165-172, 2017 <https://dx.doi.org/10.1093/ndt/gfw310>

Krieter DH, Lyko R, Schneider A, Beck W, Nilsson LG, Amdahl M, Rosenkranz AR, Kirsch A, Wanner C: Clinical performance of medium cutoff hemodialysis versus high-flux hemodialysis and high-volume hemodiafiltration. *Nephrology Dialysis Transplantation,* 31**:** i496, 2016 <http://dx.doi.org/10.1093/ndt/gfw194.19>

## Krishnasamy 2020

Krishnasamy R, Hawley CM, Jardine MJ, Roberts MA, Cho Y, Wong M, Heath A, Nelson CL, Sen S, Mount PF, Pascoe EM, Vergara LA, Paul-Brent PA, Toussaint ND, Johnson DW, Hutchison CA: A tRial Evaluating Mid Cut-Off Value Membrane Clearance of Albumin and Light Chains in HemoDialysis Patients: A Safety Device Study. *Blood Purification***:** 1-11, 2020 <https://dx.doi.org/10.1159/000505567>

## Lim 2020

*Lim J-H, Jeon Y, Yook J-M, Choi S-Y, Jung H-Y, Choi J-Y, Park S-H, Kim C-D, Kim Y-L, Cho J-H: Medium cut-off dialyzer improves erythropoiesis stimulating agent resistance in a hepcidin-independent manner in maintenance hemodialysis patients: results from a randomized controlled trial. *Sci,* 10, 2020 10.1038/s41598-020-73124-x

Lim JH, Park Y, Jeong KH, Jung HY, Choi JY, Park SH, Kim CD, Kim YL, Cho JH: Randomized controlled trial of medium cut-off or high-flux dialyzer on quality-of-life outcomes in maintenance hemodialysis patients. *Nephrology Dialysis Transplantation,* 34 (Supplement 1)**:** a643, 2019 <http://dx.doi.org/10.1093/ndt/gfz102.SuO011>

*Lim JH, Park Y, Yook JM, Choi SY, Jung HY, Choi JY, Park SH, Kim CD, Kim YL, Cho JH: Randomized controlled trial of medium cut-off versus high-flux dialyzers on quality of life outcomes in maintenance hemodialysis patients. *Sci,* 10**:** 7780, 2020 <https://dx.doi.org/10.1038/s41598-020-64622-z>

## Maduell 2020

Maduell F, Broseta JJ, Rodas L, Montagud-Marrahi E, Rodriguez-Espinosa D, Hermida E, Arias-Guillen M, Fontsere N, Vera M, Gomez M, Gonzalez B, Rico N: Comparison of Solute Removal Properties Between High-Efficient Dialysis Modalities in Low Blood Flow Rate. *Therap Apher Dial,* 24**:** 387-392, 2020 <https://dx.doi.org/10.1111/1744-9987.13440>

## Maduell 2019

Maduell F, Rodas L, Broseta JJ, Gomez M, Xipell Font M, Molina A, Montagud-Marrahi E, Guillen E, Arias-Guillen M, Fontsere N, Vera Rivera M, Rico N: High-permeability alternatives to current dialyzers performing both high-flux hemodialysis and postdilution online hemodiafiltration. *Artif Organs,* 43**:** 1014-1021, 2019 <https://dx.doi.org/10.1111/aor.13480>

## Ostogic 2020

Ostojic A, Markovic R: p1105. Can better efficacy of middle weight molecules removal in dialysis patients treated with medium cut-off membranes predict superiority over conventional high flux dialysers? *Nephrology Dialysis Transplantation,* 35, 2020 10.1093/ndt/gfaa142.P1105

## Penny 2020

Penny J, Salerno FR, Hur L, McIntyre C: p1062 Expanded dialysis (HDx): is there an impact on patient reported symptoms? *Nephrology Dialysis Transplantation,* 35, 2020 10.1093/ndt/gfaa142.P1062

## Santos 2021

Alba S, Macias N, Vega A, Abad S, Aragoncillo I, Cruzado L, Linares T, Gomez JML: Different dialyisis techniques, same anticoagulant dose? *Nephrology Dialysis Transplantation,* 34 (Supplement 1)**:** a515, 2019 http://dx.doi.org/10.1093/ndt/gfz103.SP463

*Santos A, Macías N, Vega A, Abad S, Linares T, Aragoncillo I, Cruzado L, Pascual C, Goicoechea M, López-Gómez JM: Efficacy of enoxaparin in preventing coagulation during high-flux haemodialysis, expanded haemodialysis and haemodiafiltration. Clin Kidney J, 14: 1120-1125, 2021 10.1093/ckj/sfaa057

## Sevinc 2020

Sevinc M, Hasbal NB, Yilmaz V, Basturk T, Ahbap E, Sakaci T, Ozcafer PN, Unsal A: Comparison of Circulating Levels of Uremic Toxins in Hemodialysis Patients Treated with Medium Cut-Off Membranes and High-Flux Membranes: Theranova in Sisli Hamidiye Etfal (THE SHE) Randomized Control Study. *Blood Purification***:** 1-10, 2020 <https://dx.doi.org/10.1159/000508061>

## Viramontes Horner 2020

Viramontes Horner D, Kolhe N, Leung J, Fluck R, Selby N, Taal M: P1078. Impact of a medium cut-off dialyzer on skin autofluorescence in haemodialysis patients. *Nephrology Dialysis Transplantation,* 35, 2020 10.1093/ndt/gfaa142.P1078

## Weiner 2020

Weiner DE, Falzon L, Skoufos L, Bernardo A, Beck W, Xiao M, Tran H: Efficacy and Safety of Expanded Hemodialysis with the Theranova 400 Dialyzer. *Clinical Journal of the American Society of Nephrology,* 15**:** 1310-1319, 2020 10.2215/cjn.01210120

## Yeter 2020

Yeter HH, Korucu B, Akcay OF, Derici K, Derici U, Arinsoy T: Effects of medium cut-off dialysis membranes on inflammation and oxidative stress in patients on maintenance hemodialysis. *Int Urol Nephrol,* 13**:** 13, 2020 <https://dx.doi.org/10.1007/s11255-020-02562-3>

## Yick 2019

Yick L, Choi B: Improvements in quality of life and outcome in patients using HDx dialyzers compared to high-flux dialyzers...2019 Canadian Association of Nephrology Nurses and Technicians Annual Conference, 24-26 October 2019, Edmonton, Alberta. *CANNT Journal,* 29**:** 30-30, 2019

## Zickler 2017

Zickler D, Schindler R, Storr M, Willy K, Trojanowicz B, Martus P, Ulrich C, Liehr K, Henning C, Pawlak M, Templin M, Hulko M, Bohler T, Werner K, Glomb MA, Fiedler R, Girndt M: The use of medium cut-off (MCO) membranes in chronic dialysis patients modulates inflammation: Lessons from a randomized clinical trial. *Nephrology Dialysis Transplantation,* 31**:** i230, 2016 <http://dx.doi.org/10.1093/ndt/gfw170.25>

*Zickler D, Schindler R, Willy K, Martus P, Pawlak M, Storr M, Hulko M, Boehler T, Glomb MA, Liehr K, Henning C, Templin M, Trojanowicz B, Ulrich C, Werner K, Fiedler R, Girndt M: Medium Cut-Off (MCO) Membranes Reduce Inflammation in Chronic Dialysis Patients-A Randomized Controlled Clinical Trial. *PLoS ONE,* 12**:** e0169024, 2017 https://dx.doi.org/10.1371/journal.pone.0169024
