# Supplementary material for: Effects of Medium Cut-Off Versus High-Flux Hemodialysis Membranes on Biomarkers: A Systematic Review and Meta-Analysis
Source: Can J Kidney Health Dis. 2022 Jan 18;9:20543581211067090. doi: 10.1177/20543581211067090 (PMC8777328; doi:10.1177/20543581211067090)
Supplement: sj-pptx-5-cjk-10.1177_20543581211067090 – Supplemental material for Effects of Medium Cut-Off Versus High-Flux Hemodialysis Membranes on Biomarkers: A Systematic Review and Meta-Analysis [file sj-pptx-5-cjk-10.1177_20543581211067090.pptx]

## Slide 1
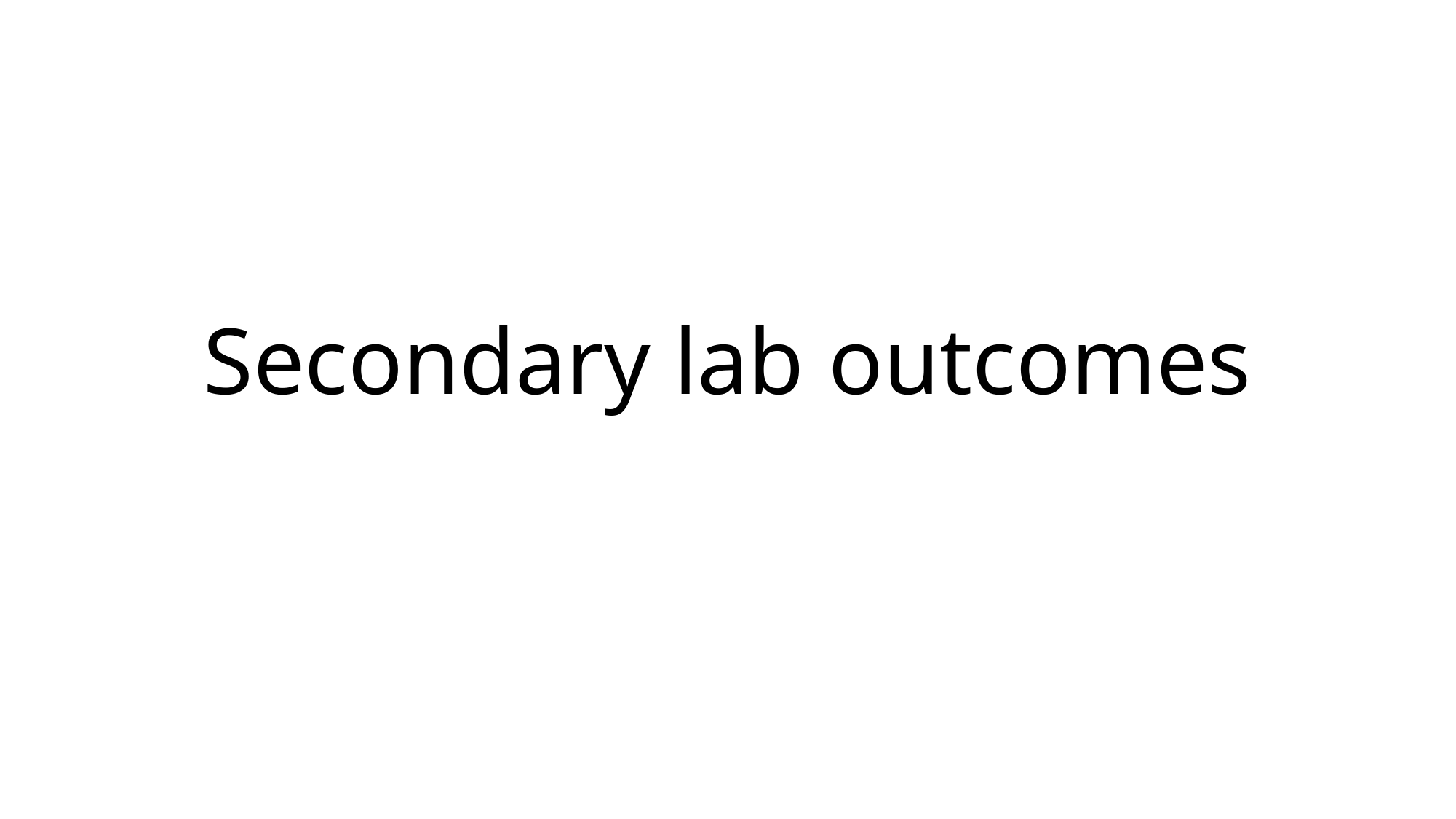

# Secondary lab outcomes

## Slide 2
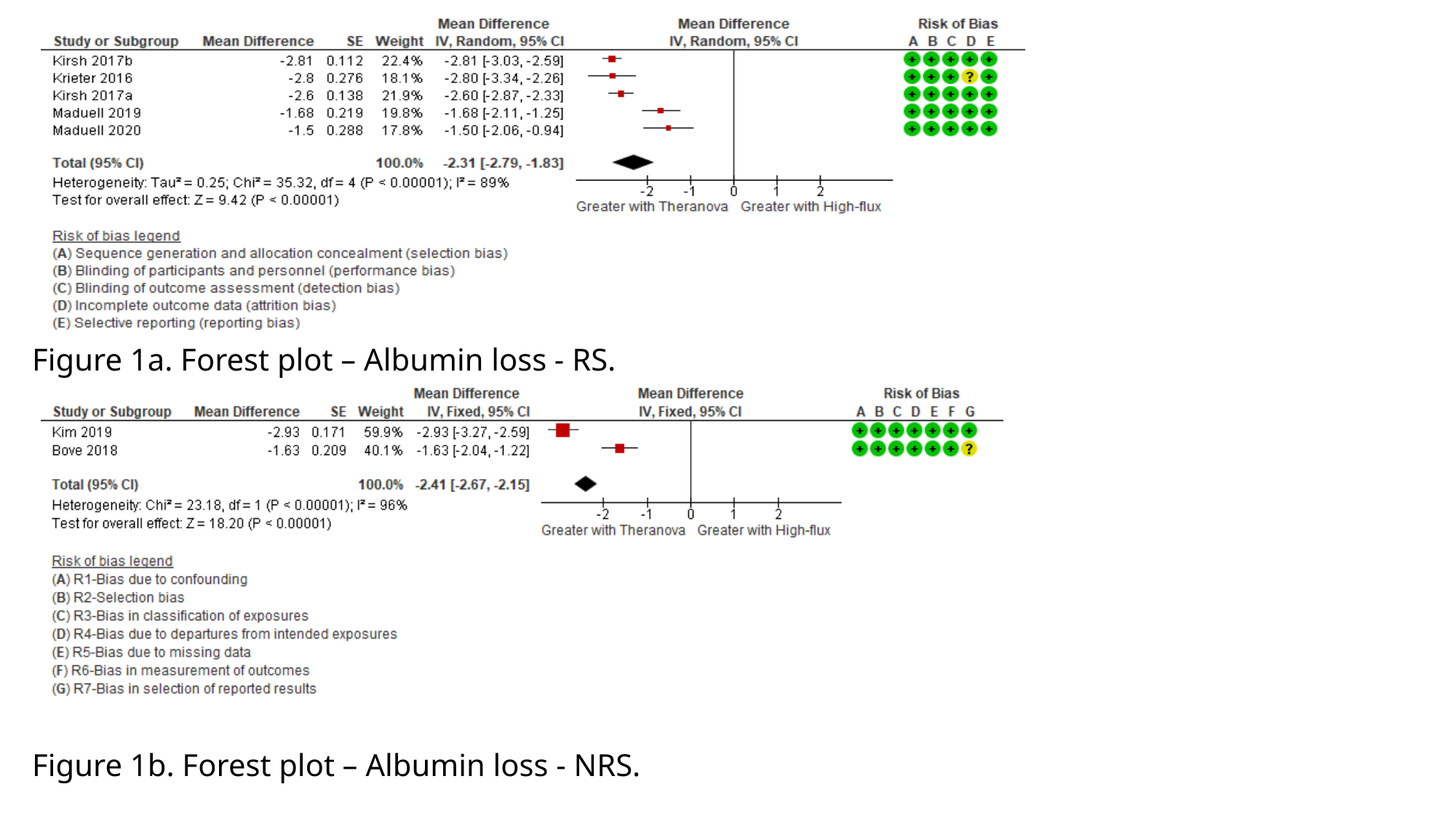

# Figure 1a. Forest plot – Albumin loss - RS.
Figure 1b. Forest plot – Albumin loss - NRS.

## Slide 3
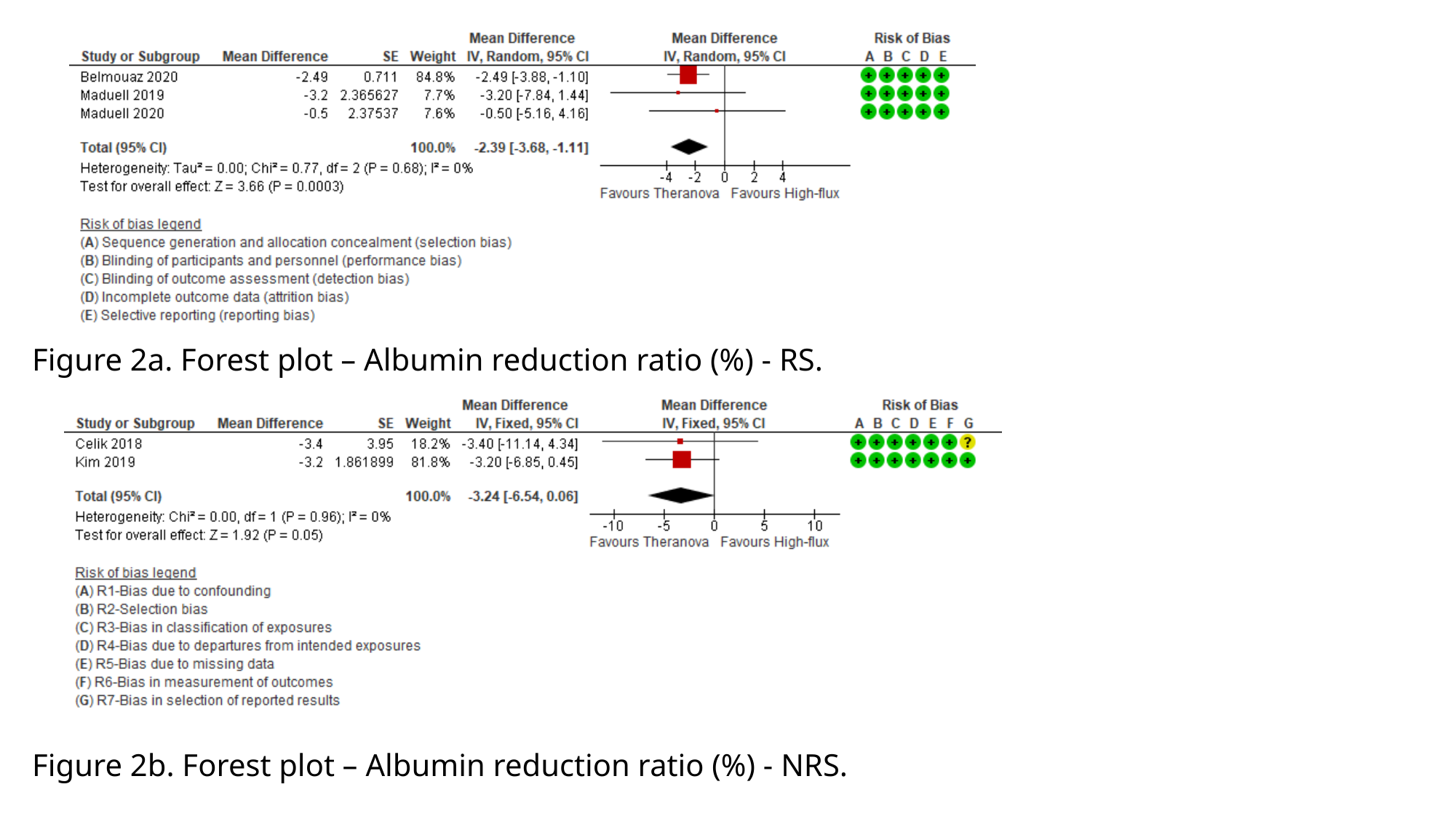

# Figure 2a. Forest plot – Albumin reduction ratio (%) - RS.
Figure 2b. Forest plot – Albumin reduction ratio (%) - NRS.

## Slide 4
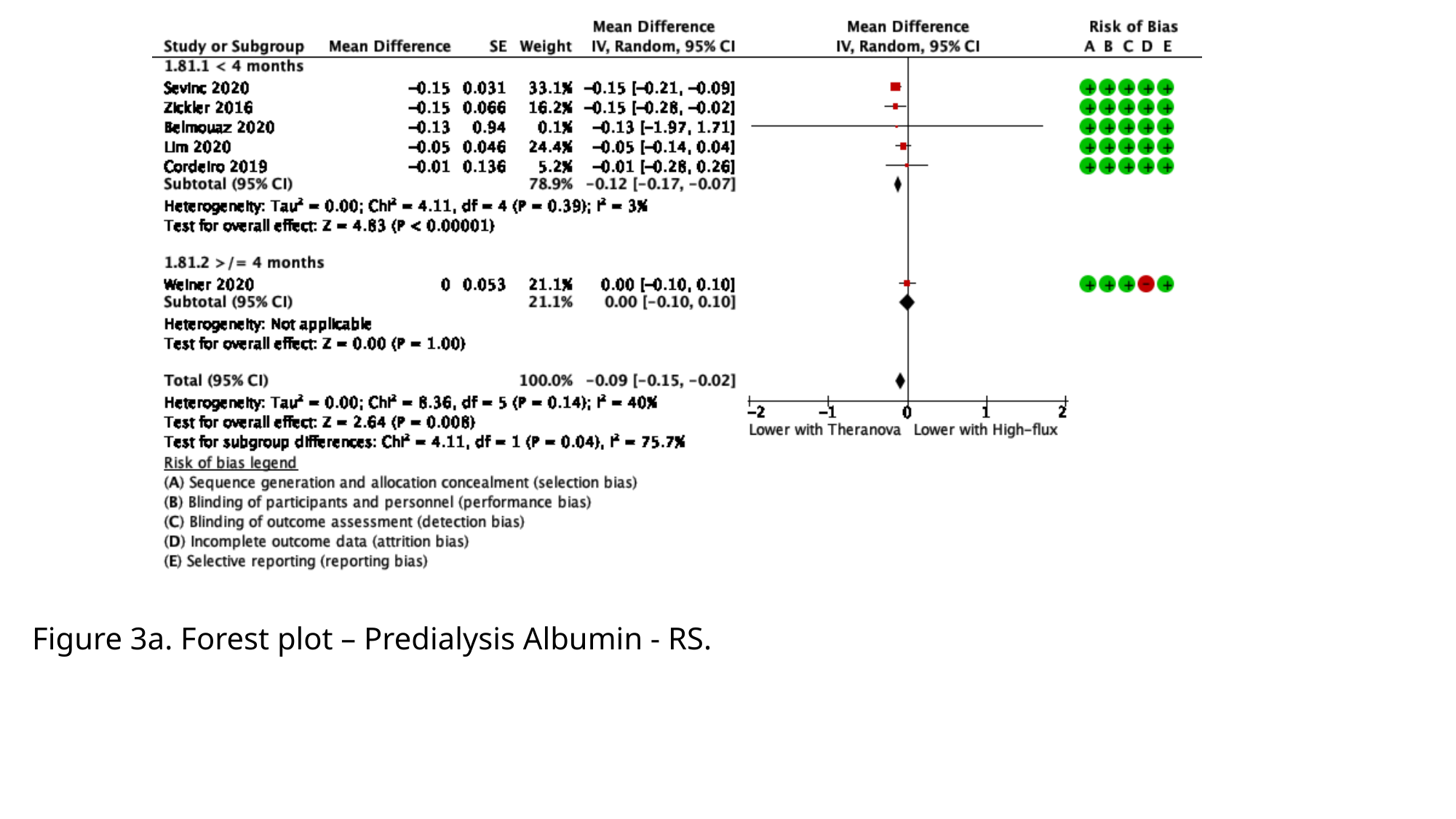

# Figure 3a. Forest plot – Predialysis Albumin - RS.

## Slide 5
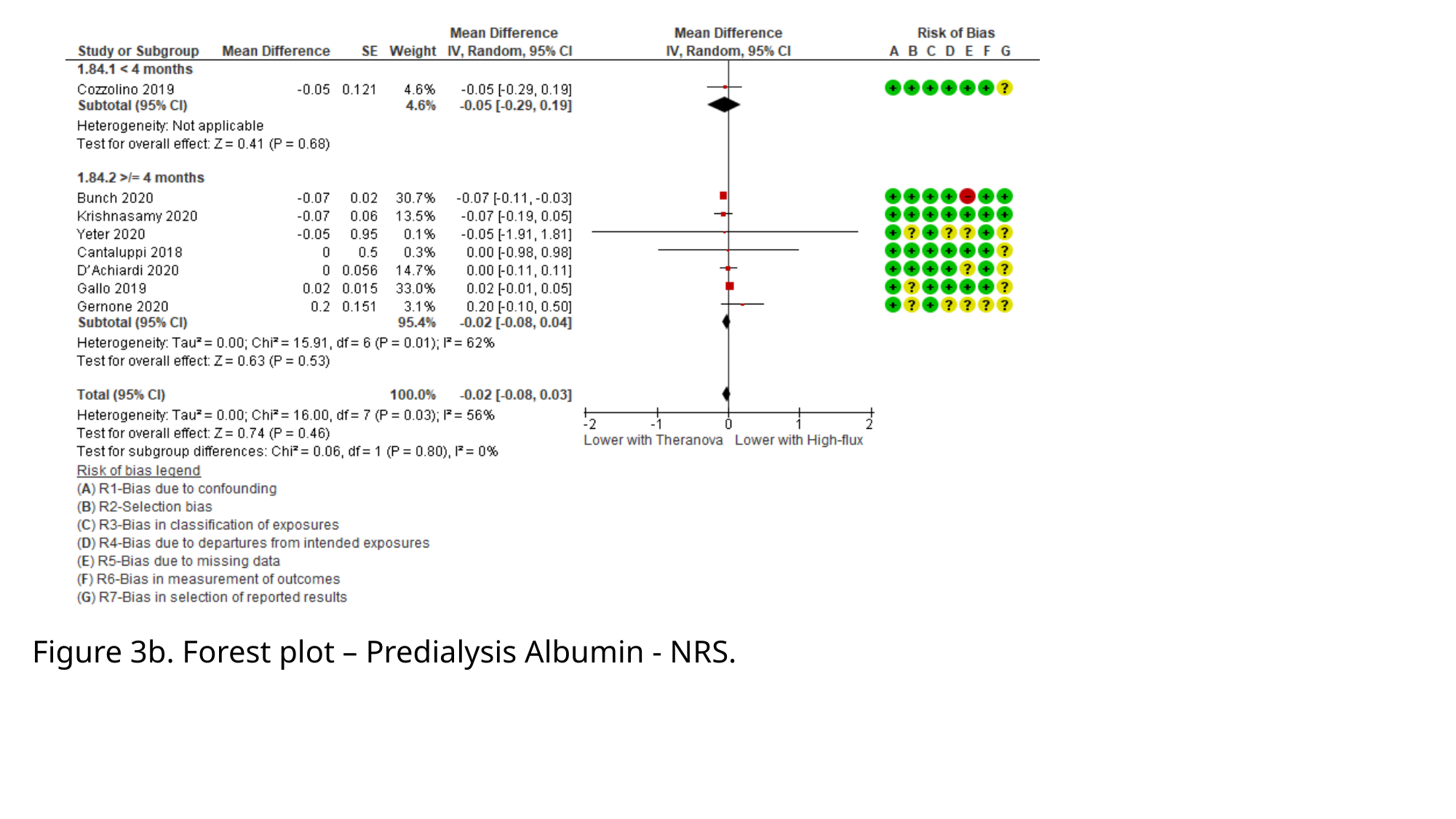

Figure 3b. Forest plot – Predialysis Albumin - NRS.

## Slide 6
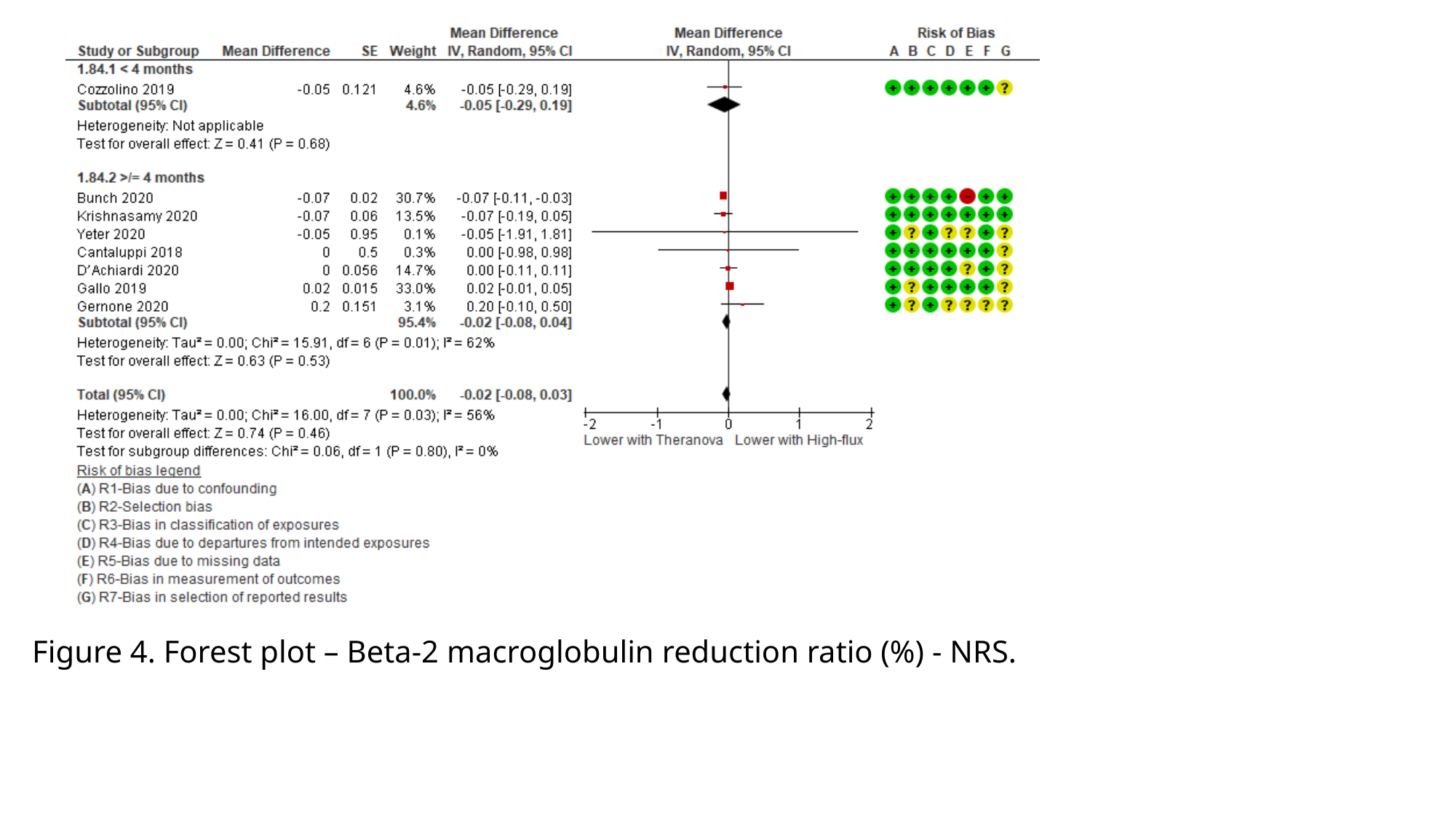

Figure 4. Forest plot – Beta-2 macroglobulin reduction ratio (%) - NRS.

## Slide 7
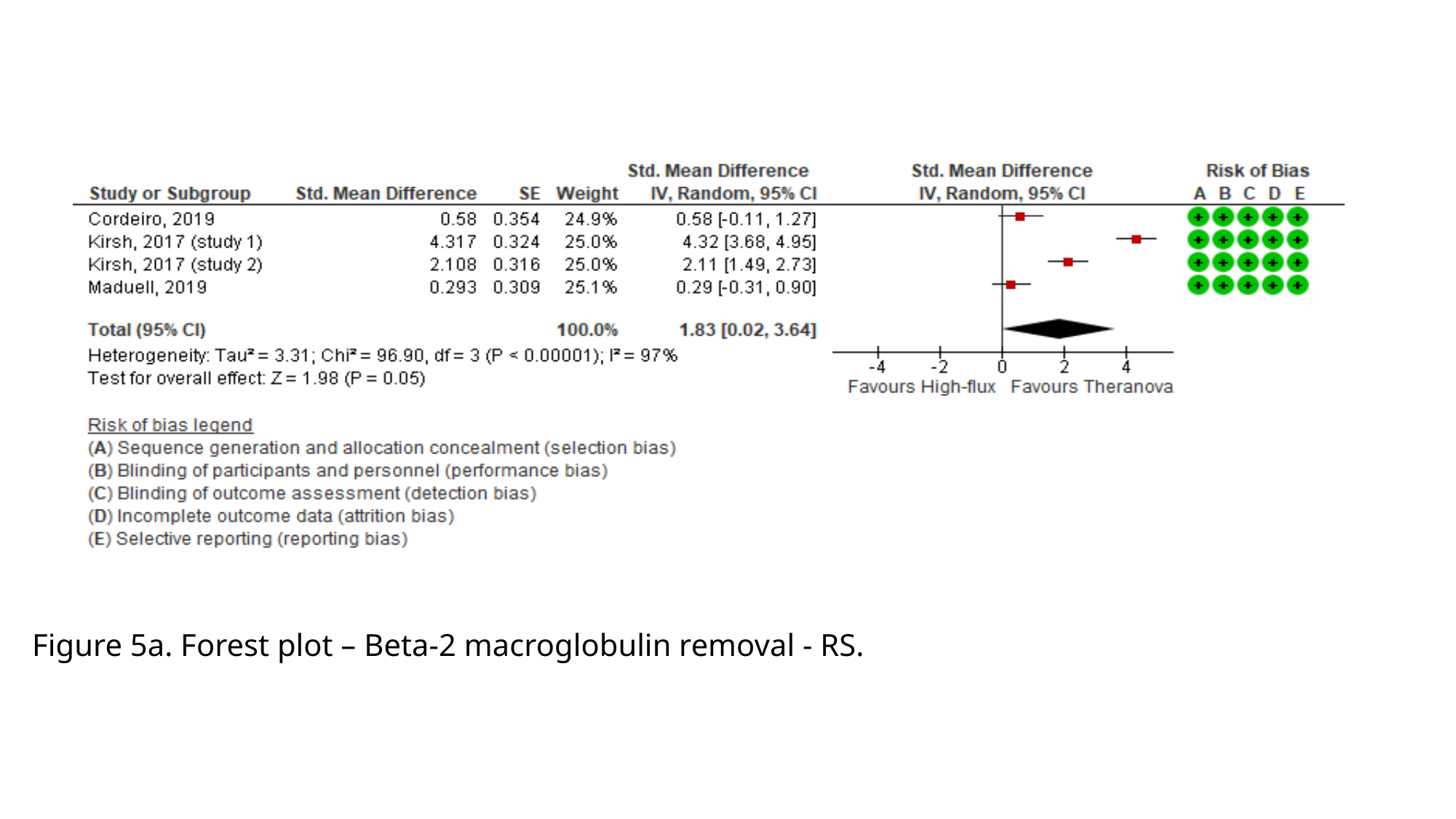

# Figure 5a. Forest plot – Beta-2 macroglobulin removal - RS.

## Slide 8
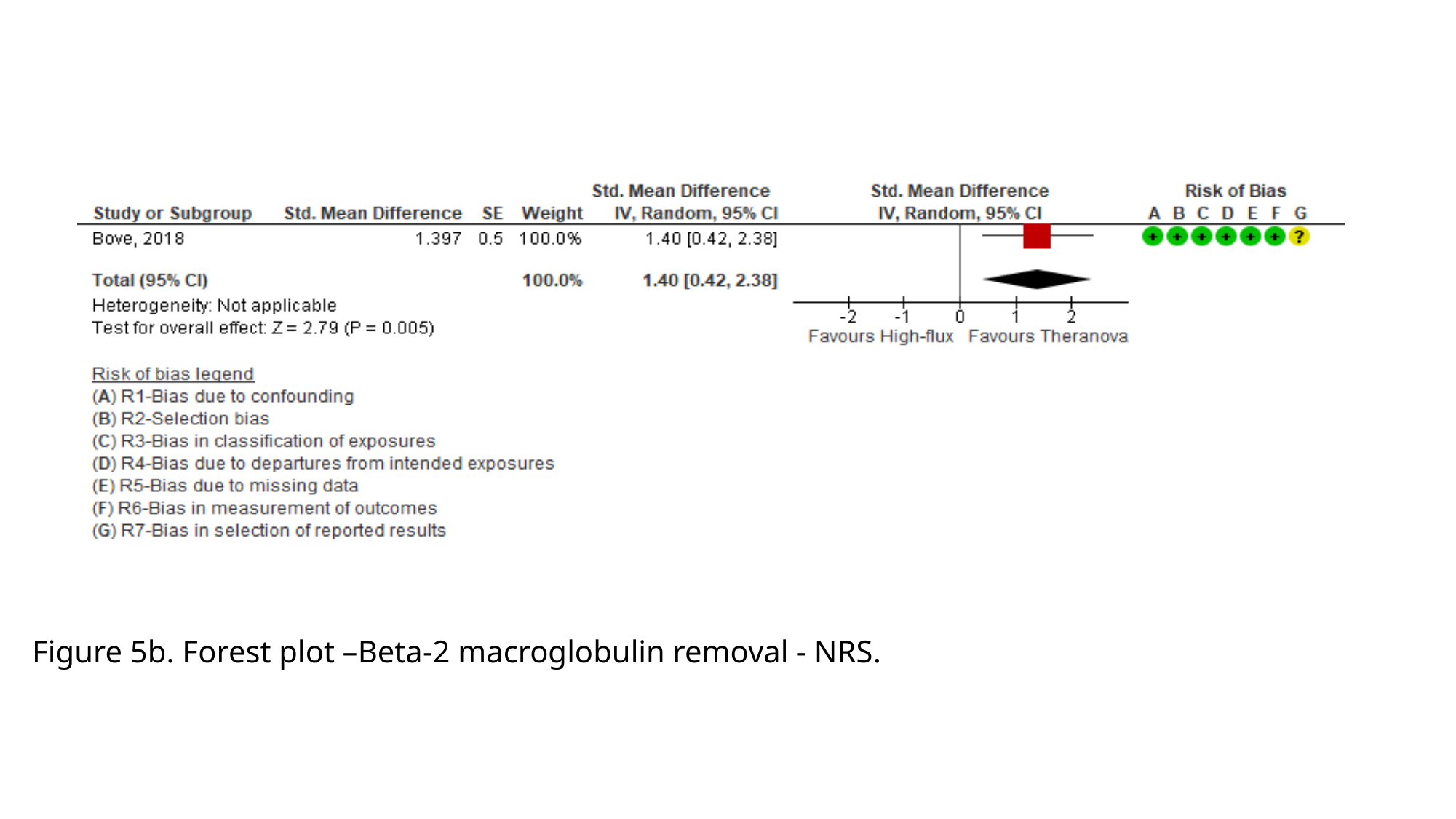

Figure 5b. Forest plot –Beta-2 macroglobulin removal - NRS.

## Slide 9
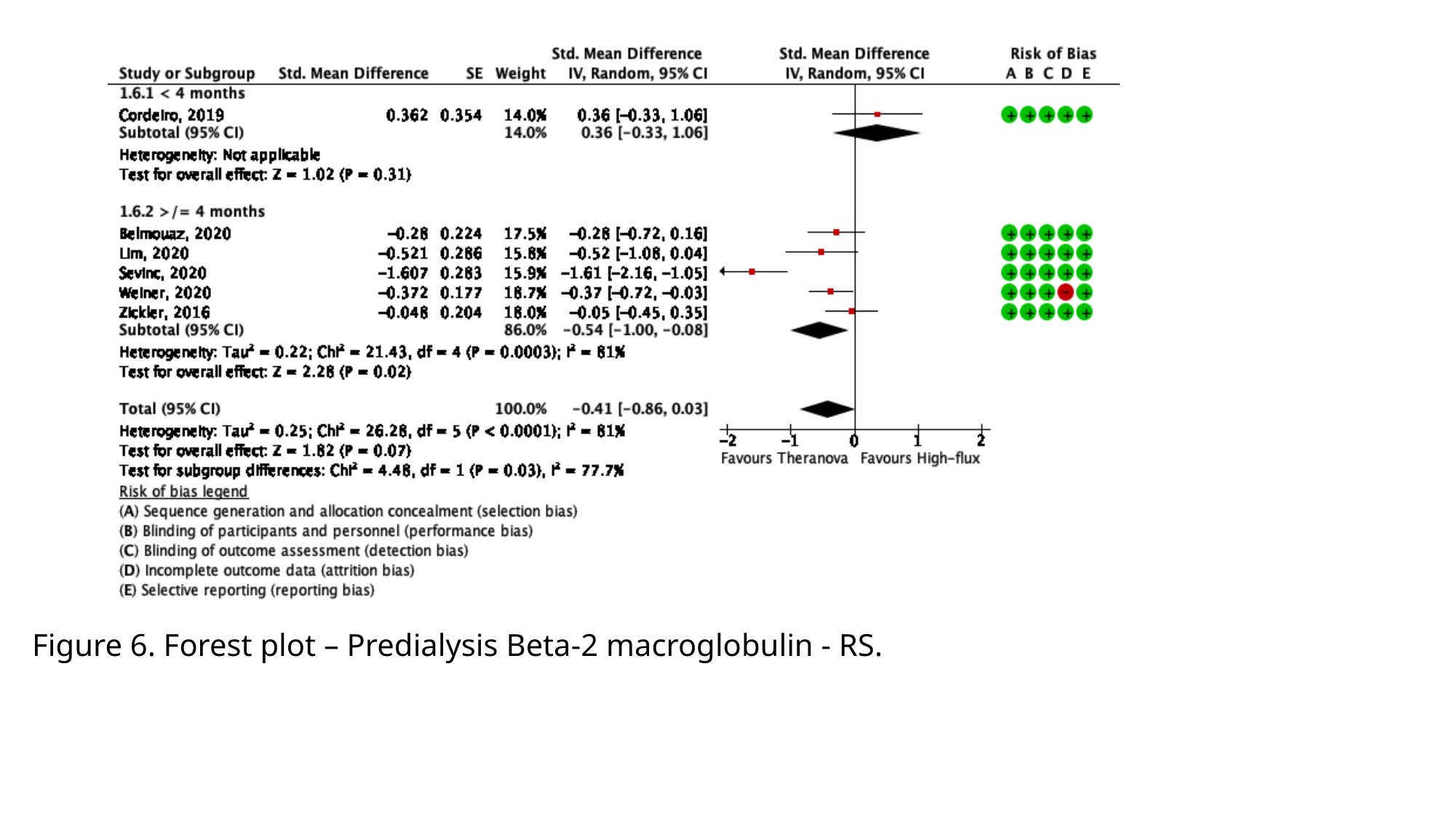

# Figure 6. Forest plot – Predialysis Beta-2 macroglobulin - RS.

## Slide 10
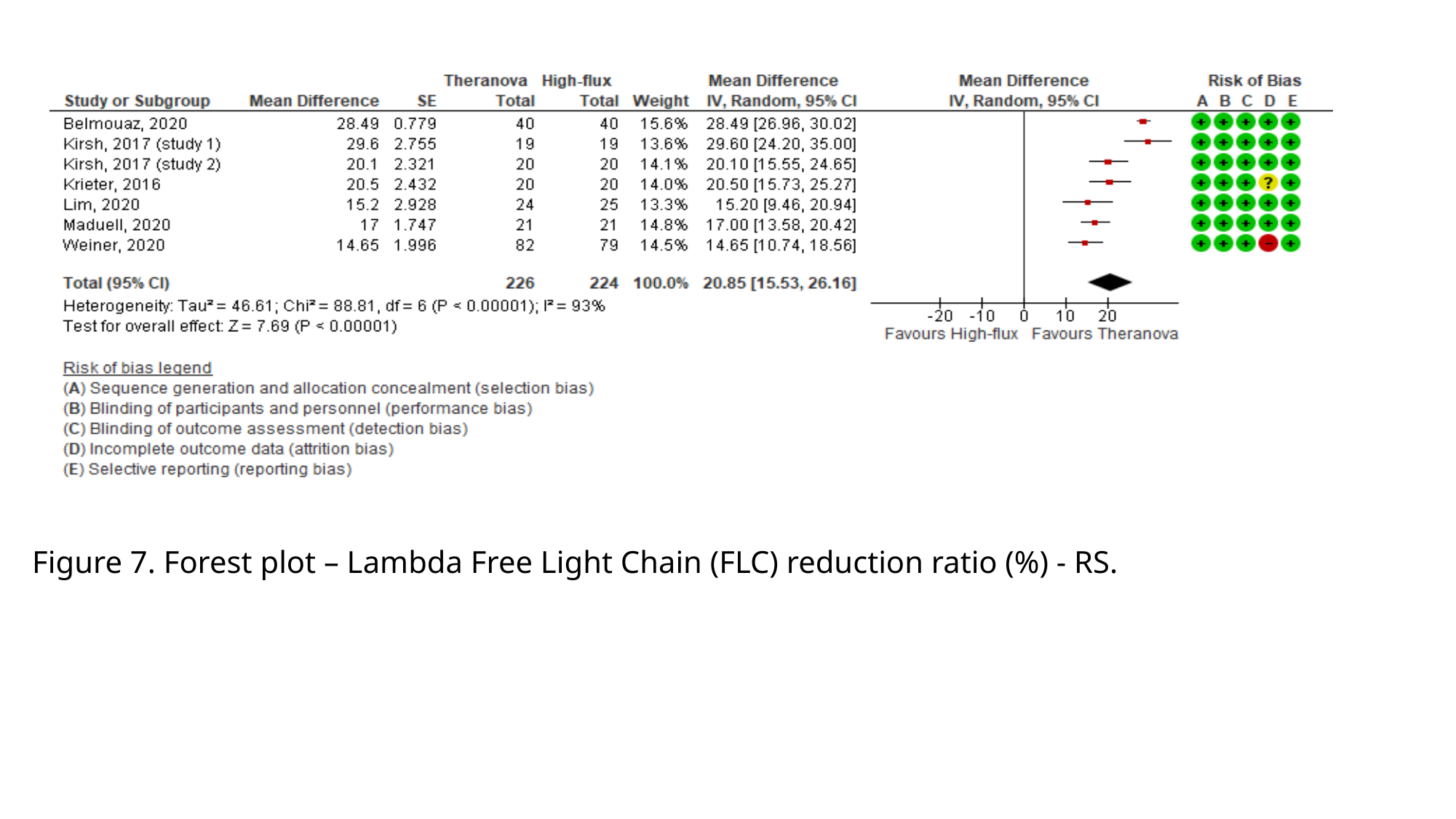

Figure 7. Forest plot – Lambda Free Light Chain (FLC) reduction ratio (%) - RS.

## Slide 11
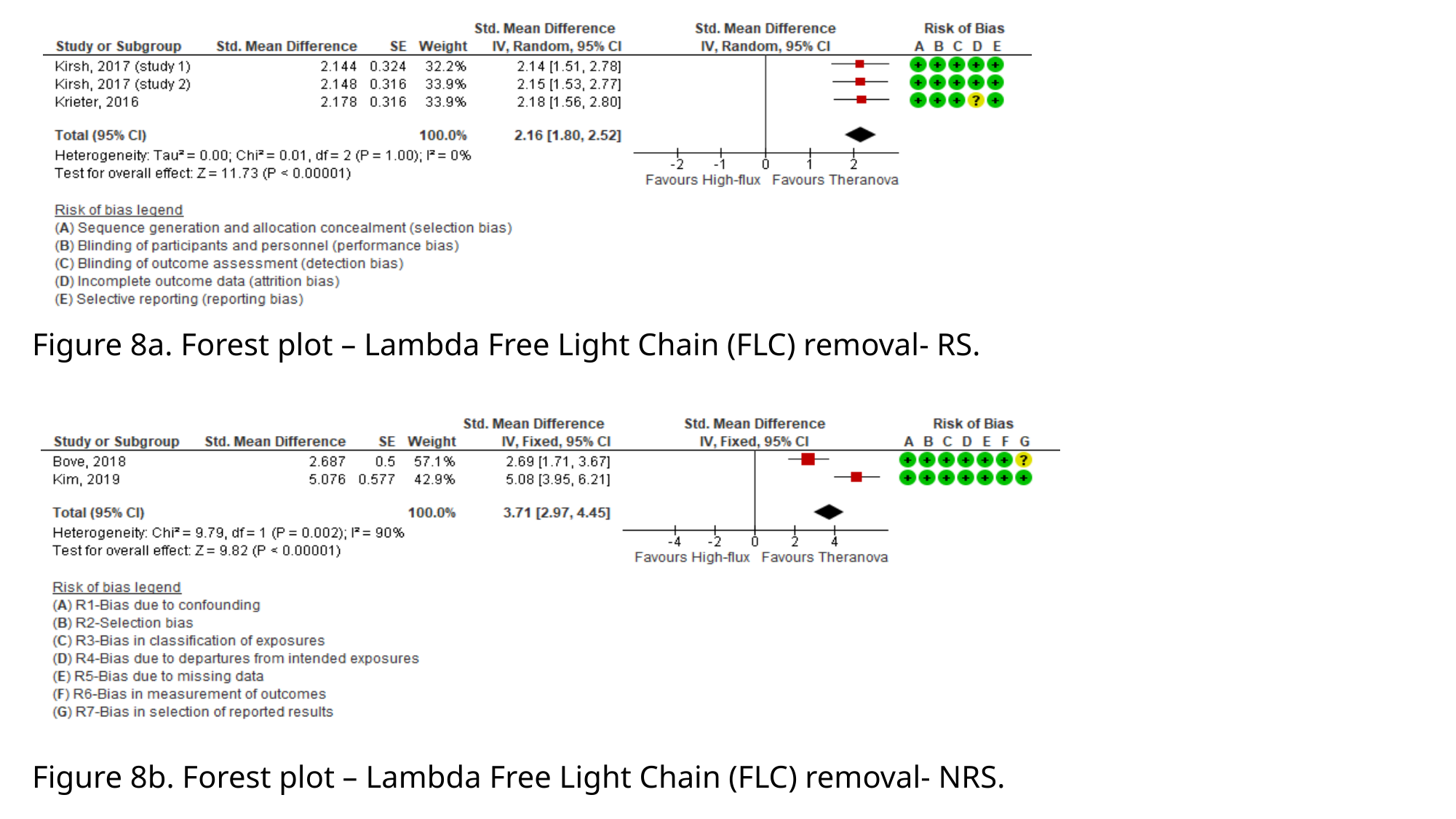

Figure 8a. Forest plot – Lambda Free Light Chain (FLC) removal- RS.
Figure 8b. Forest plot – Lambda Free Light Chain (FLC) removal- NRS.

## Slide 12
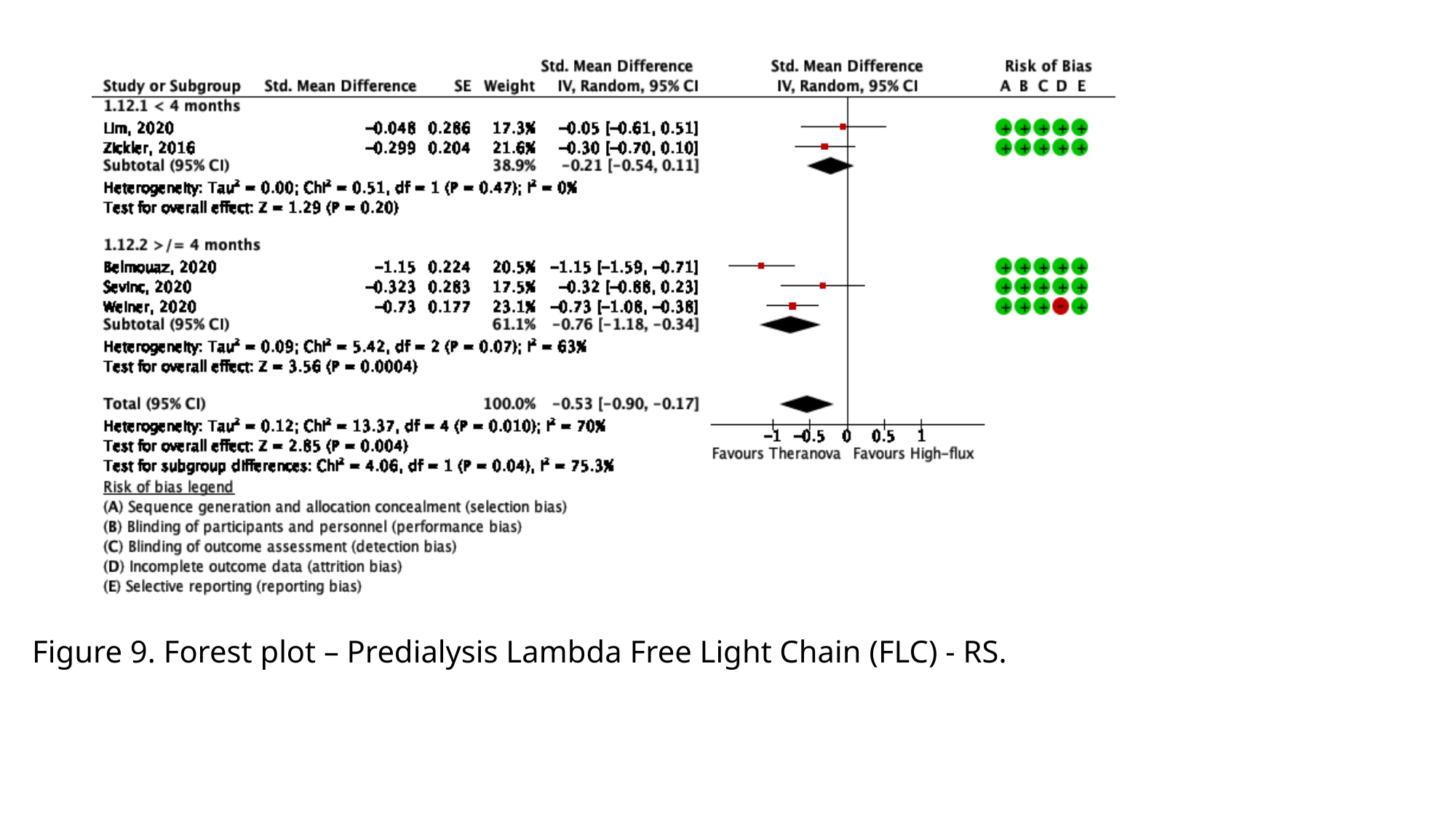

Figure 9. Forest plot – Predialysis Lambda Free Light Chain (FLC) - RS.

## Slide 13
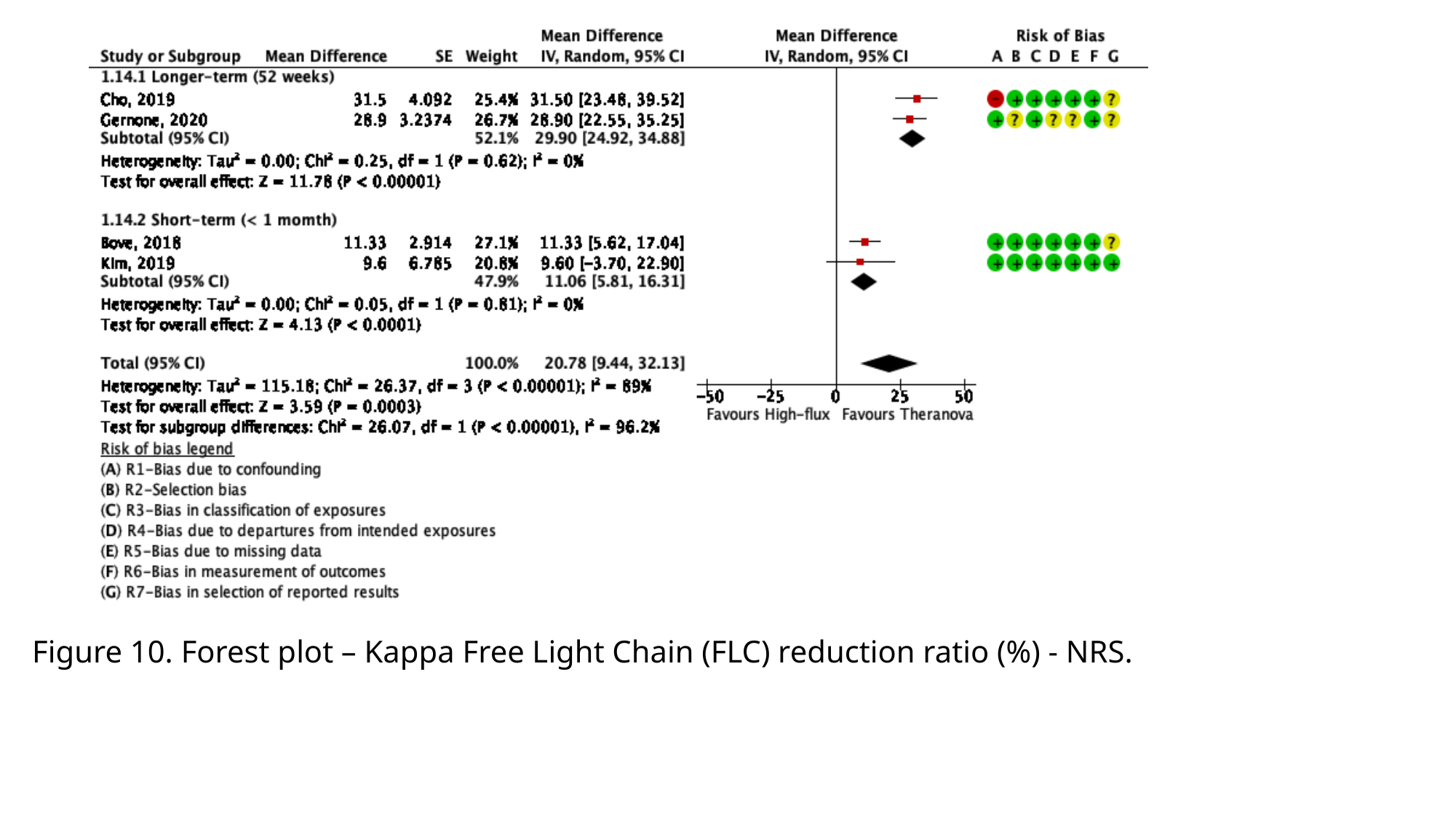

Figure 10. Forest plot – Kappa Free Light Chain (FLC) reduction ratio (%) - NRS.

## Slide 14
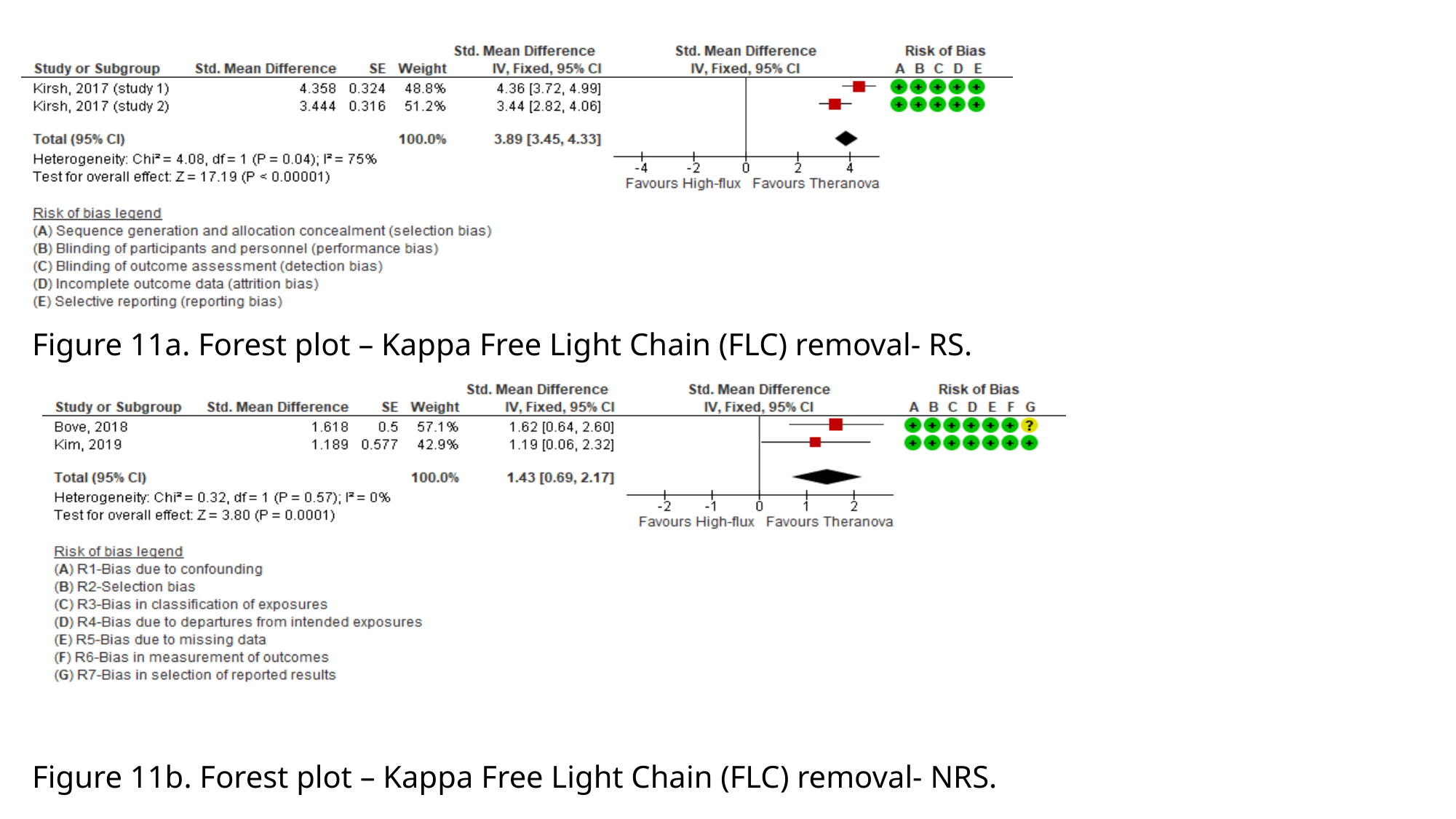

Figure 11a. Forest plot – Kappa Free Light Chain (FLC) removal- RS.
Figure 11b. Forest plot – Kappa Free Light Chain (FLC) removal- NRS.

## Slide 15
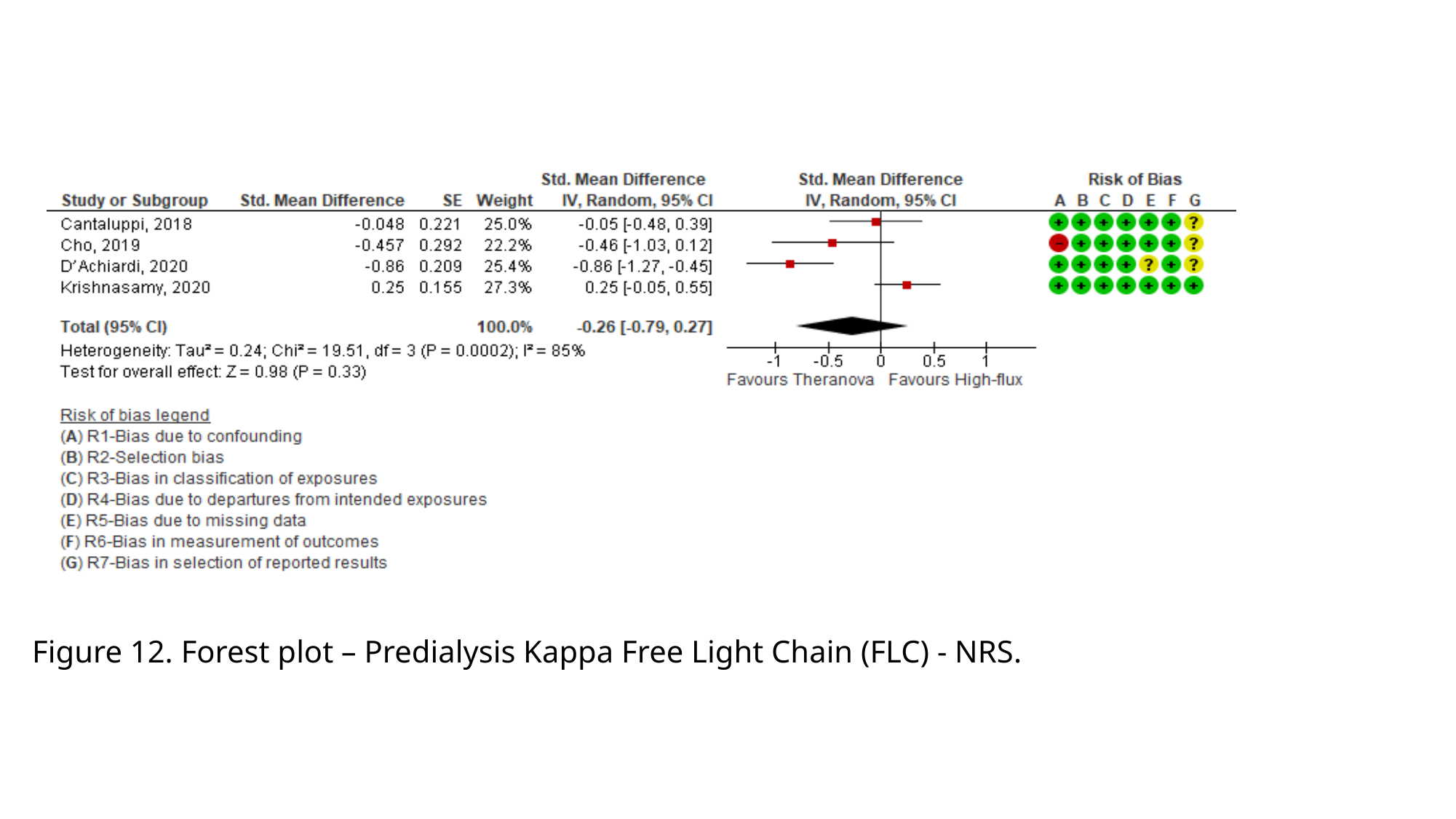

Figure 12. Forest plot – Predialysis Kappa Free Light Chain (FLC) - NRS.

## Slide 16
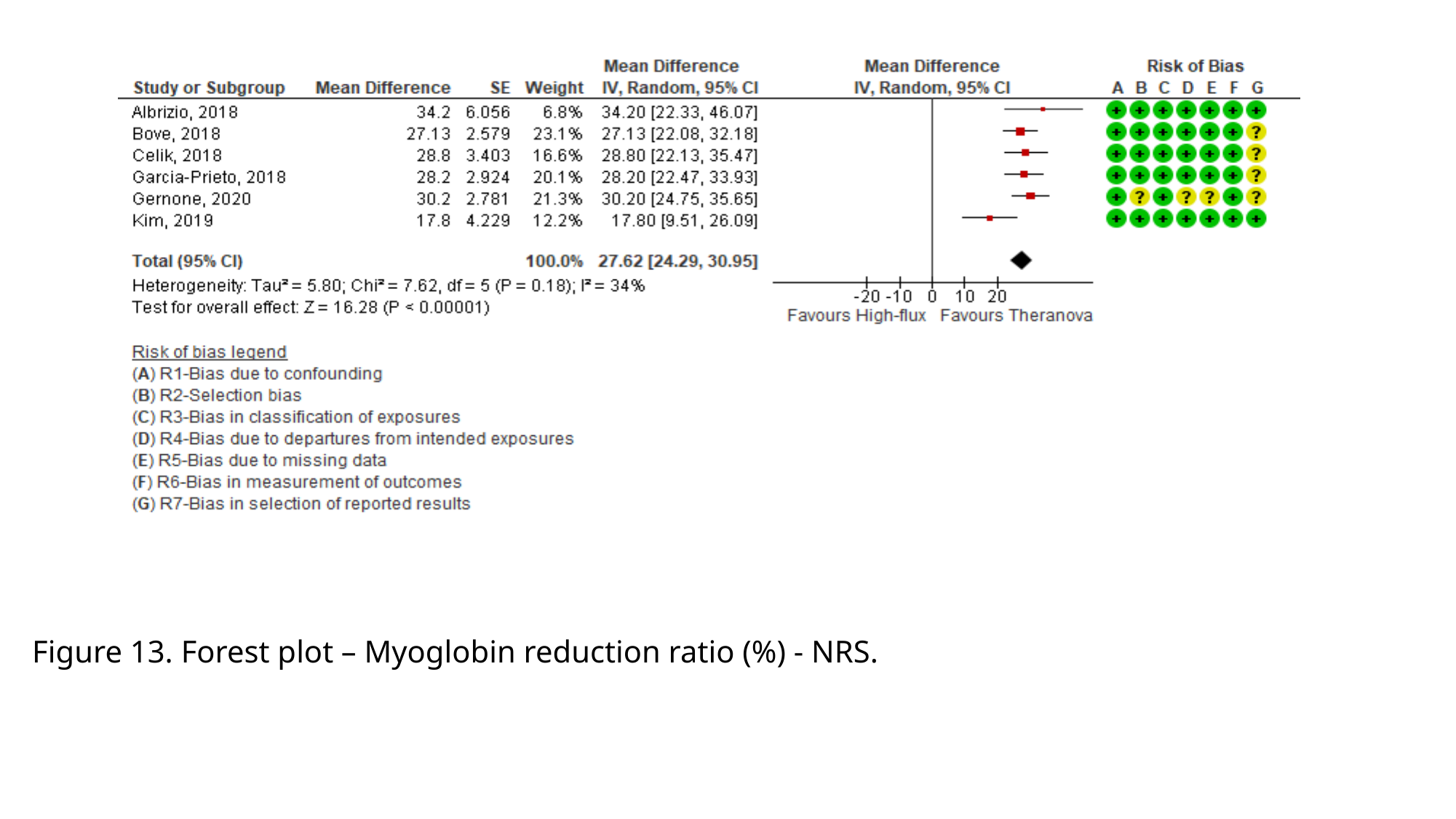

Figure 13. Forest plot – Myoglobin reduction ratio (%) - NRS.

## Slide 17
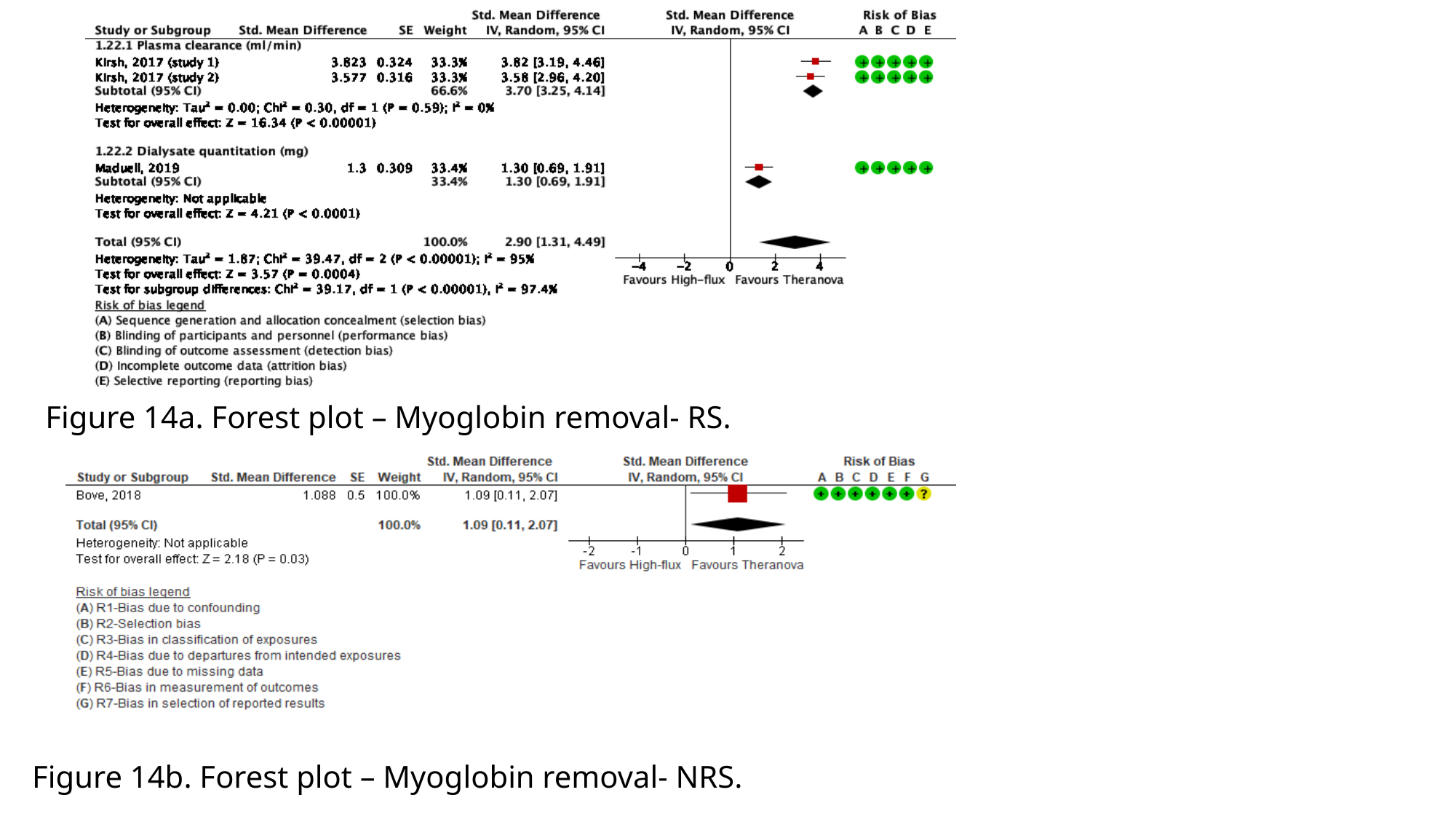

Figure 14a. Forest plot – Myoglobin removal- RS.
Figure 14b. Forest plot – Myoglobin removal- NRS.

## Slide 18
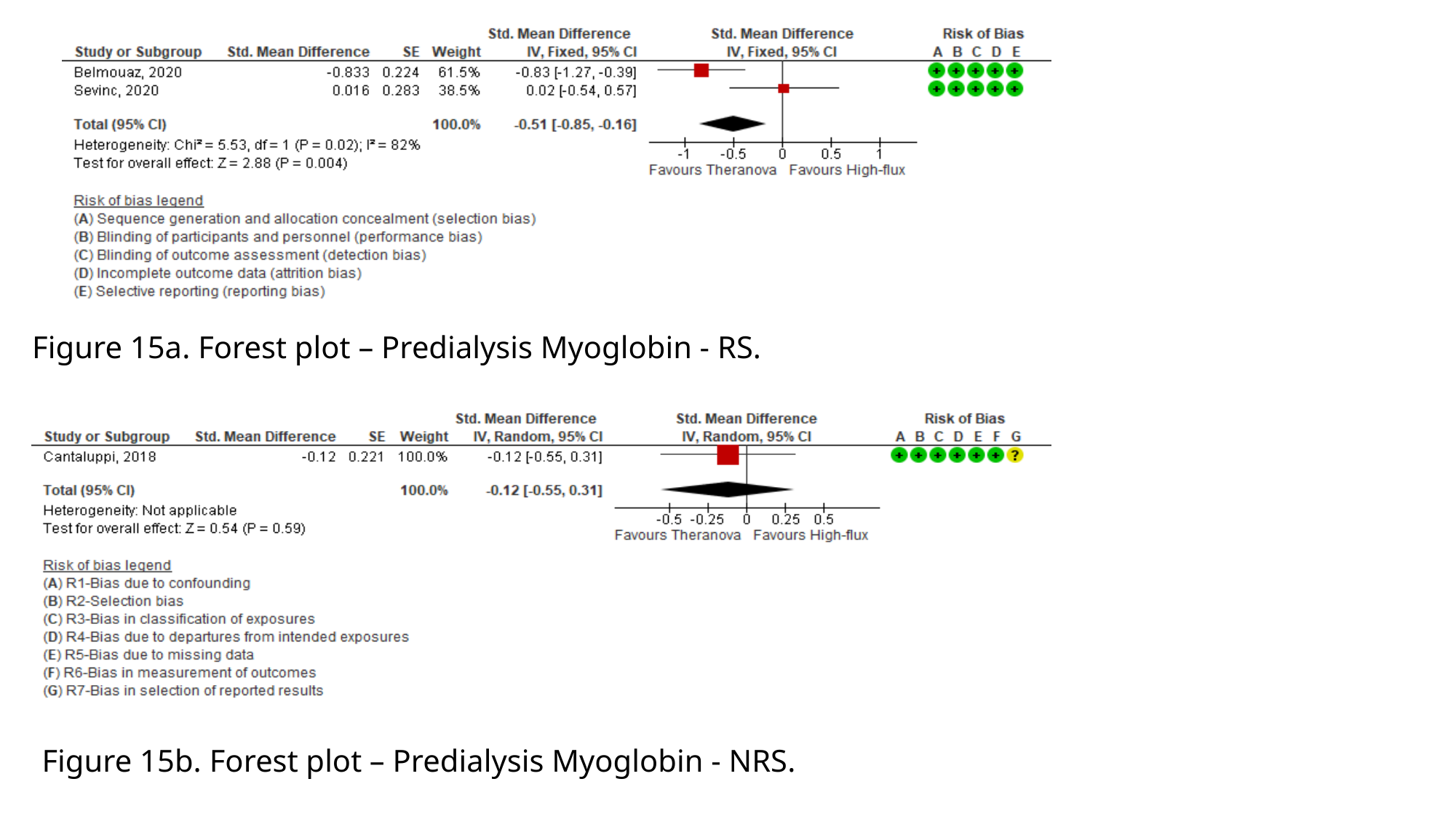

Figure 15a. Forest plot – Predialysis Myoglobin - RS.
Figure 15b. Forest plot – Predialysis Myoglobin - NRS.

## Slide 19
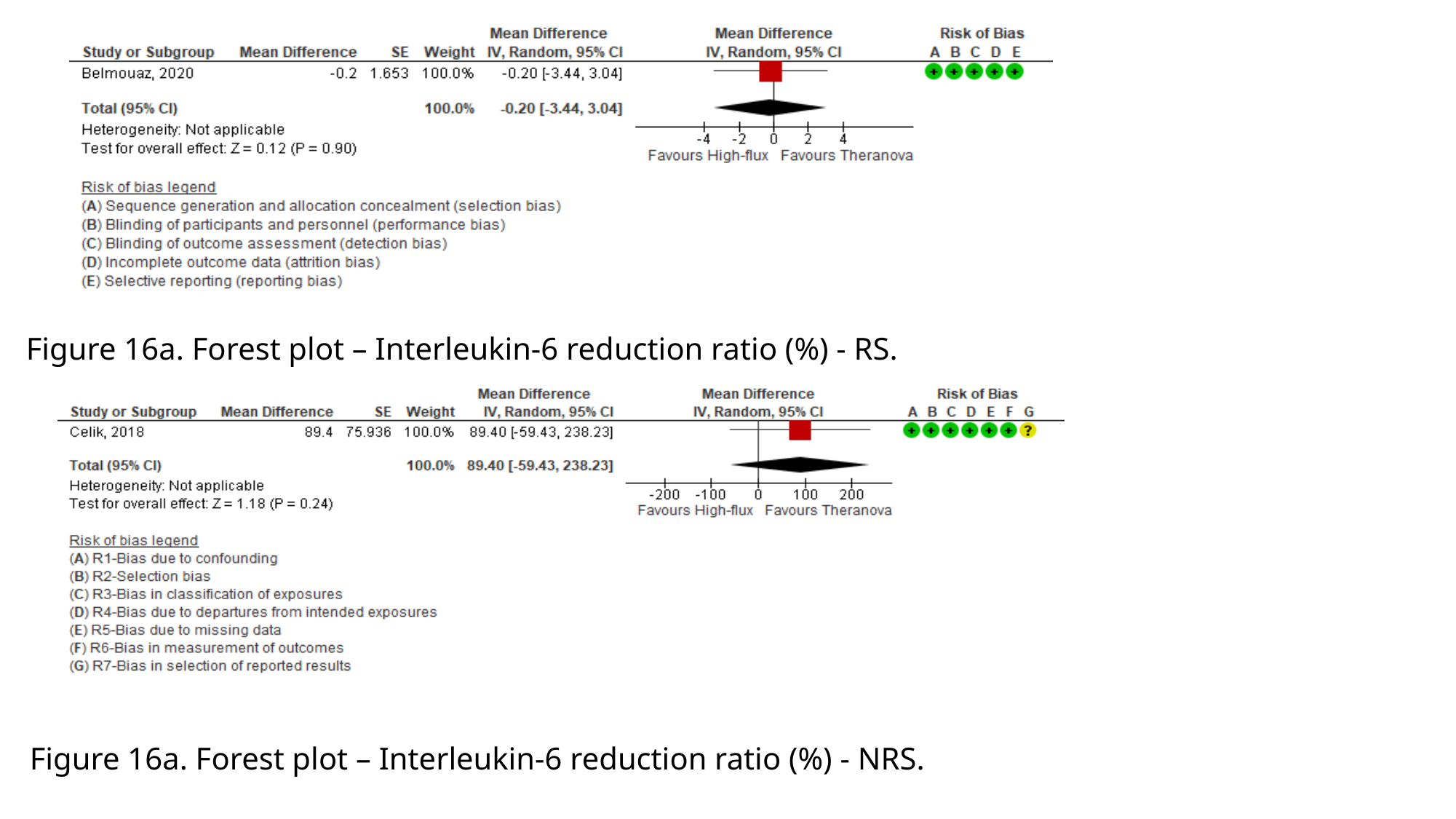

Figure 16a. Forest plot – Interleukin-6 reduction ratio (%) - RS.
Figure 16a. Forest plot – Interleukin-6 reduction ratio (%) - NRS.

## Slide 20
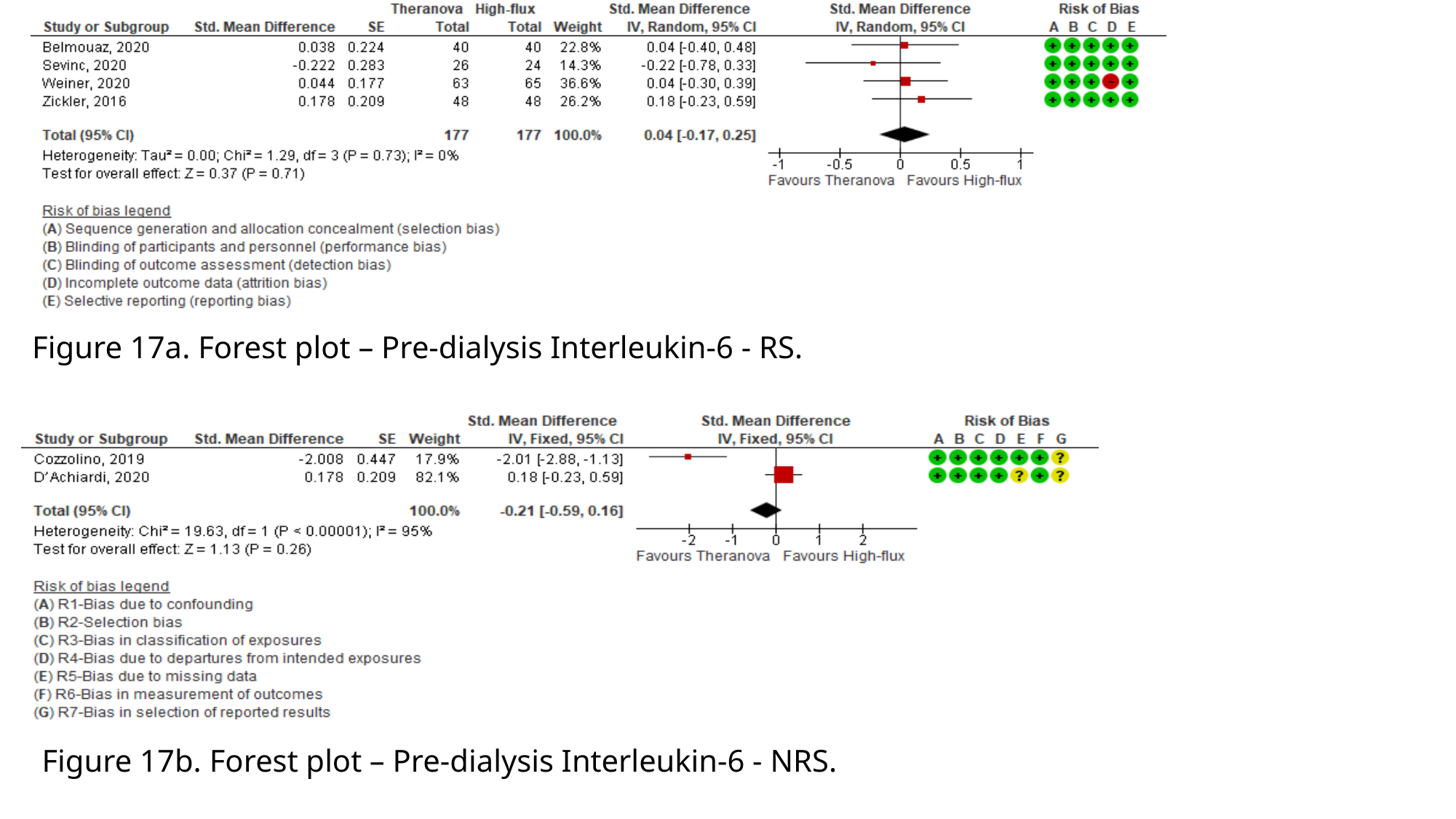

Figure 17a. Forest plot – Pre-dialysis Interleukin-6 - RS.
Figure 17b. Forest plot – Pre-dialysis Interleukin-6 - NRS.

## Slide 21
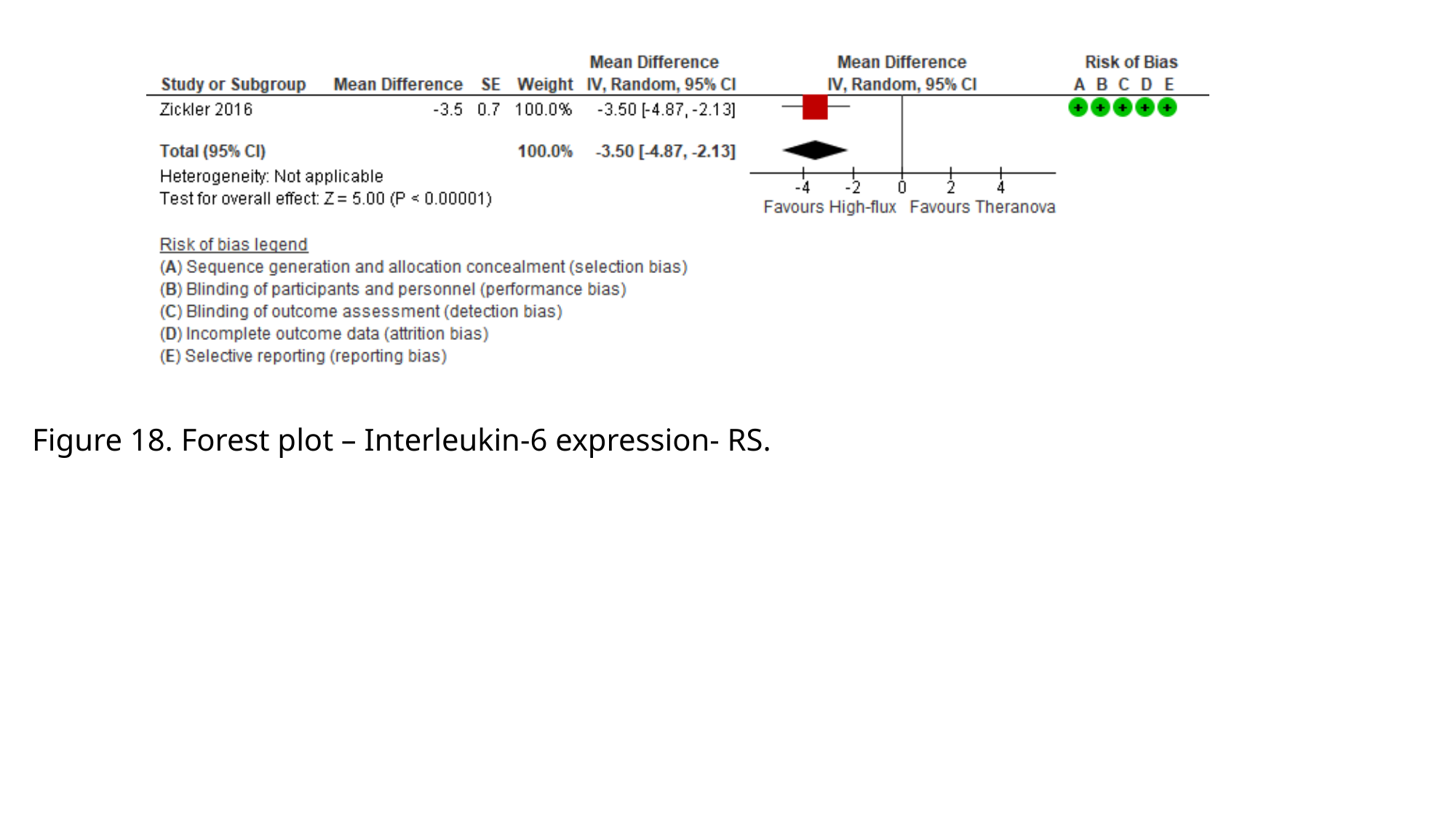

Figure 18. Forest plot – Interleukin-6 expression- RS.

## Slide 22
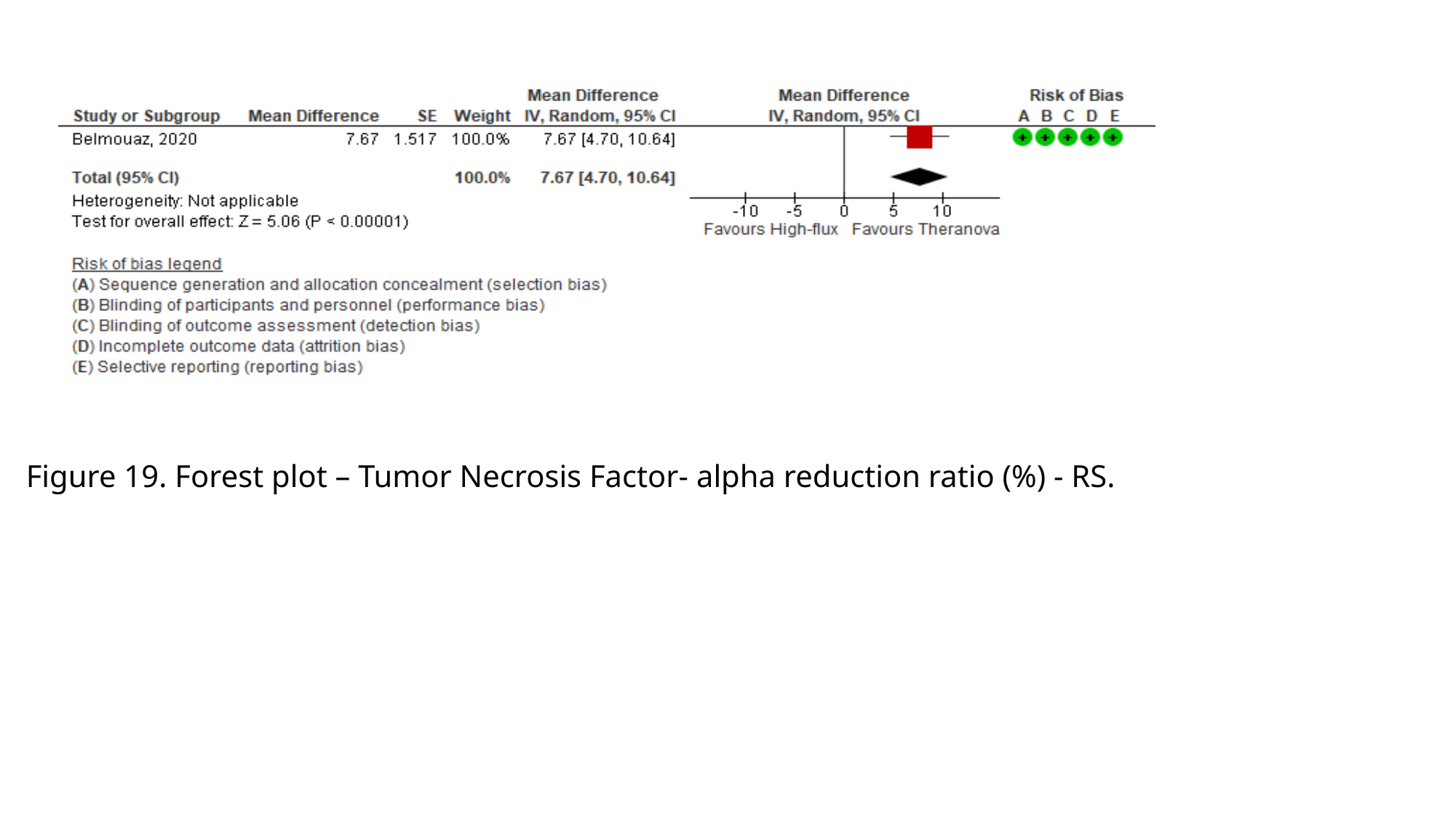

Figure 19. Forest plot – Tumor Necrosis Factor- alpha reduction ratio (%) - RS.

## Slide 23
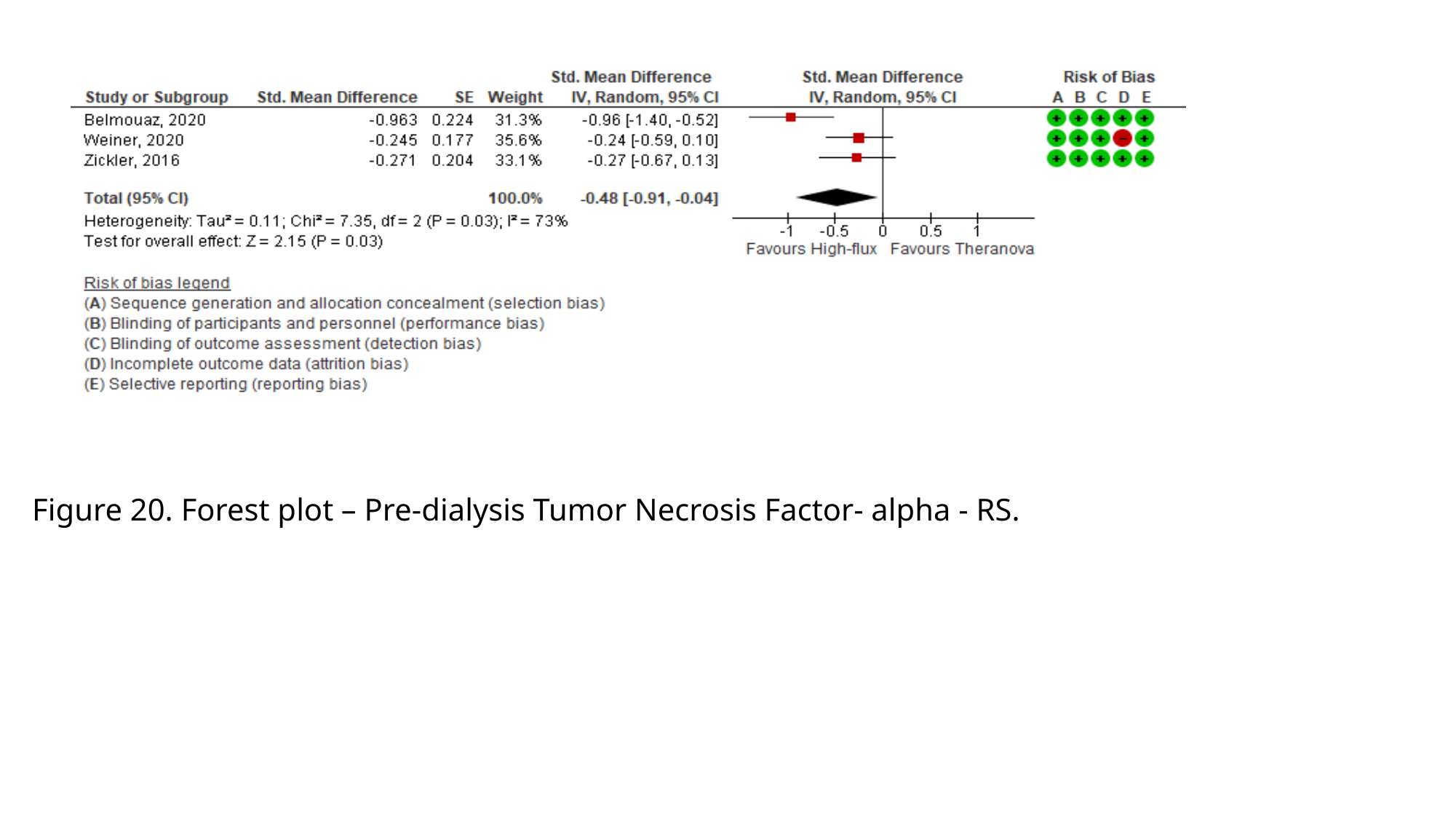

Figure 20. Forest plot – Pre-dialysis Tumor Necrosis Factor- alpha - RS.

## Slide 24
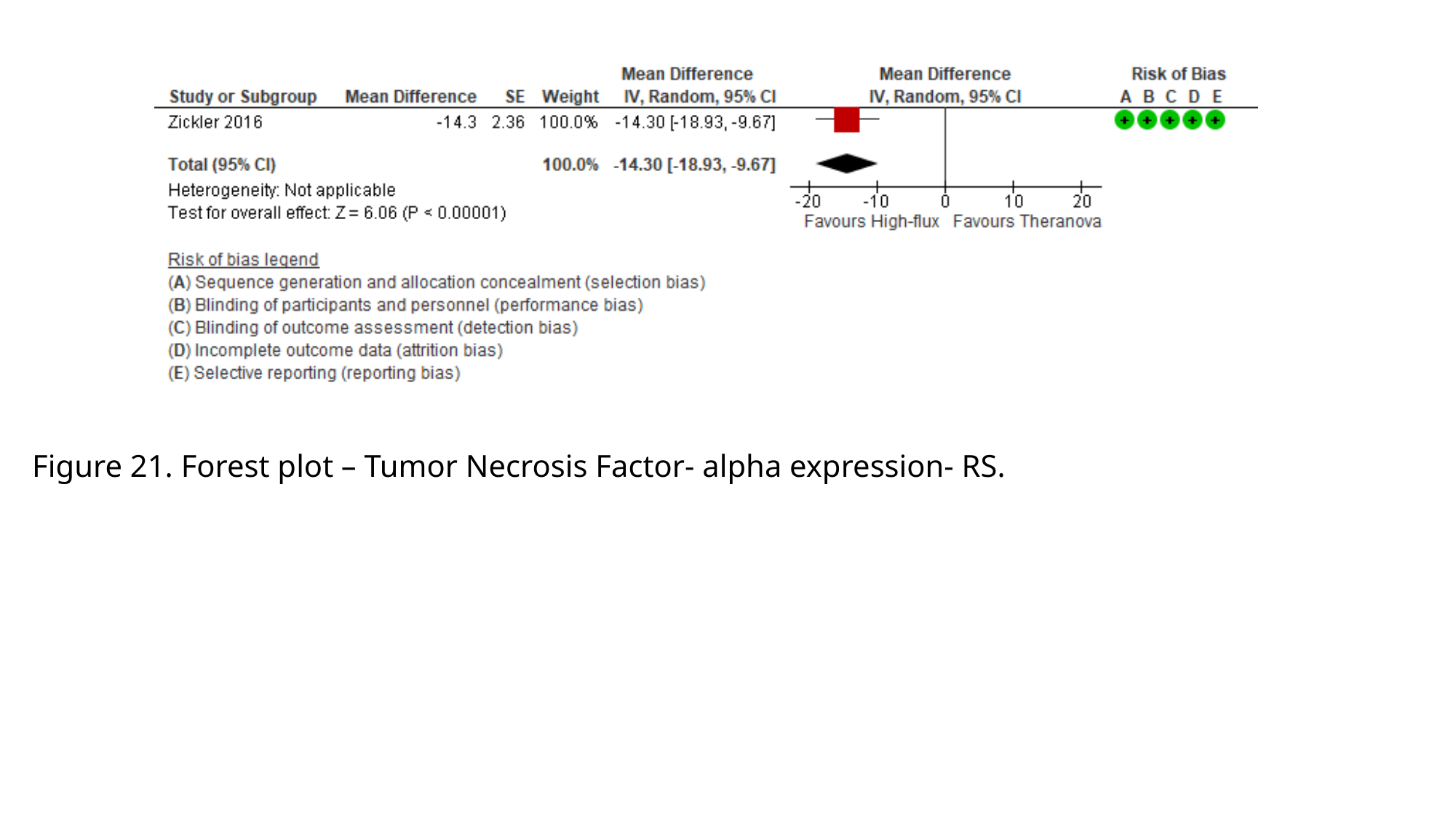

Figure 21. Forest plot – Tumor Necrosis Factor- alpha expression- RS.

## Slide 25
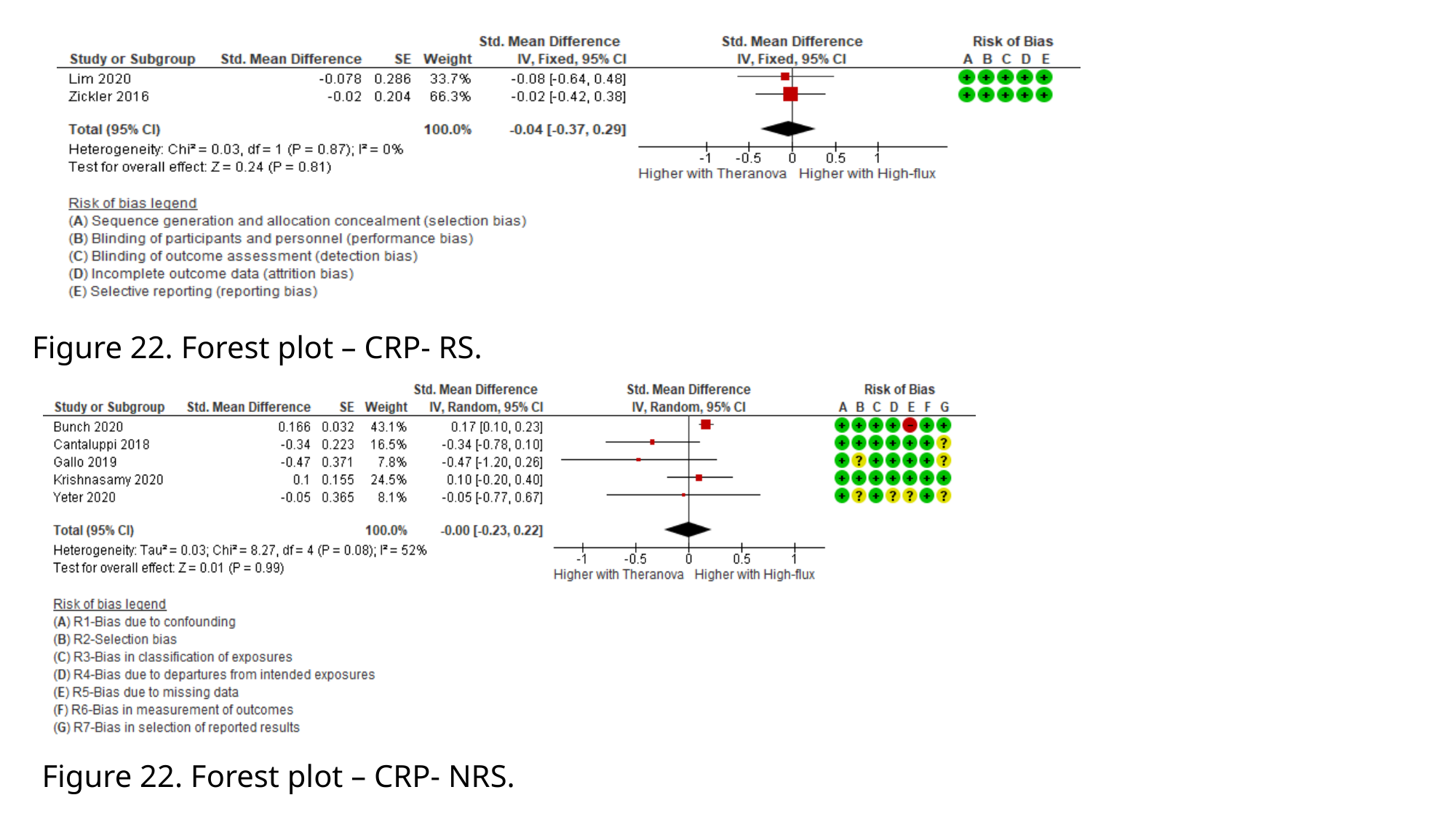

Figure 22. Forest plot – CRP- RS.
Figure 22. Forest plot – CRP- NRS.
