# Supplementary material for: Effects of Medium Cut-Off Versus High-Flux Hemodialysis Membranes on Biomarkers: A Systematic Review and Meta-Analysis
Source: Can J Kidney Health Dis. 2022 Jan 18;9:20543581211067090. doi: 10.1177/20543581211067090 (PMC8777328; doi:10.1177/20543581211067090)
Supplement: sj-docx-7-cjk-10.1177_20543581211067090 – Supplemental material for Effects of Medium Cut-Off Versus High-Flux Hemodialysis Membranes on Biomarkers: A Systematic Review and Meta-Analysis [file sj-docx-7-cjk-10.1177_20543581211067090.docx]

# Appendix G – Summary of findings tables (comprehensive)

### Table 2 – Summary of findings – albumin-related measures.

| Outcome № of participants  (studies) | **Anticipated absolute effects (95% ci)** | | Certainty | What happens |
| --- | --- | --- | --- | --- |
|  | **Without Theranova** | **Difference** |  |  |
| **Albumin loss (g)** follow up: 2 weeks № of participants: 230 (5 RS) | The mean albumin loss ranged from **0.2-0.56** g | MD **2.31 g higher** (2.79 higher to 1.83 higher) | ⨁⨁⨁⨁ high | Theranova increases albumin loss slightly. |
| **Albumin loss (g)** follow up: range 2 weeks to 3 weeks № of participants: 28 (2 NRS) | The mean albumin loss ranged from **0.07-0.26** g | MD **2.41 g higher** (2.67 higher to 2.15 higher) | ⨁⨁⨁◯ moderate ^a^ | Theranova likely increases albumin loss slightly. |
| **Albumin reduction ratio (%)** follow up: range 2 weeks to 26 weeks № of participants: 162 (3 RS) | The mean albumin reduction ratio ranged from **7-11** % | MD **2.39 % higher** (3.68 higher to 1.11 higher) | ⨁⨁⨁⨁ high | Theranova increases albumin reduction ratio slightly. |
| **Albumin reduction ratio (%)** follow up: range 2 weeks to 3 weeks № of participants: 28 (2 NRS) | The mean albumin reduction ratio ranged from **2-7** % | MD **3.24 % higher** (6.54 higher to 0.06 lower) | ⨁⨁⨁◯ moderate ^b^ | Theranova likely results in little to no difference in albumin reduction ratio. |
| **Pre-dialysis serum albumin (g/dl)** subgroup with < 24 weeks follow-up follow up: range 8 weeks to 13 weeks № of participants: 305 (5 RS) | The mean pre-dialysis serum albumin ranged from **3.79-3.94** g/dl | MD **0.12 g/dl lower** (0.17 lower to 0.07 lower) | ⨁⨁⨁⨁ high | Theranova reduces pre-dialysis serum albumin slightly over the short term (< 24 weeks). |
| **Pre-dialysis serum albumin (g/dl)**  subgroup with >/=24 weeks follow-up follow up: 24 weeks № of participants: 129 (1 RS) | The mean pre-dialysis serum albumin was **4.1** g/dl | MD **0 g/dl**  (0.1 lower to 0.1 higher) | ⨁⨁⨁◯ moderate ^c^ | Theranova likely results in little to no difference in pre-dialysis serum albumin after 24 weeks of treatment. |
| **Pre-dialysis serum albumin (g/dl)** subgroup with < 24 weeks follow-up follow up: 13 weeks № of participants: 20 (1 NRS) | The mean pre-dialysis serum albumin (g/dl) - subgroup with < 4 months follow-up ranged from **3.65** g/dl | MD **0.05 g/dl lower** (0.29 lower to 0.19 higher) | ⨁⨁◯◯ low ^d^ | Theranova may result in little to no difference in pre-dialysis serum albumin over the short term (< 24 weeks). |
| **Pre-dialysis serum albumin (g/dl)** - subgroup with >/= 24 weeks follow-up follow up: range 24 weeks to 52 weeks № of participants: 2010 (7 NRS) | The mean pre-dialysis serum albumin (g/dl) - subgroup with >/= 4 months follow-up ranged from **3.1-4.05** g/dl | MD **0.02 g/dl lower** (0.08 lower to 0.04 higher) | ⨁⨁⨁◯ moderate ^c^ | Theranova likely results in little to no difference in pre-dialysis serum albumin after 24 weeks of treatment. |
| **Pre-dialysis serum albumin (g/dl))**  Subgroup analysis - RS and NRS with < 24 weeks follow-up follow up: range 8 weeks to 13 weeks № of participants: 325 (5 RS, 1 NRS) | The mean pre-dialysis serum albumin ranged from **3.76-3.94** g/dl | MD **0.12 g/dl lower** (0.16 lower to 0.07 lower) | ⨁⨁⨁⨁ high | Theranova reduces pre-dialysis serum albumin slightly within the first 24 weeks of follow-up. |
| **Pre-dialysis serum albumin (g/dl)**  Subgroup analysis - RS and NRS with >/= 24 weeks follow-up follow up: range 24 weeks to 52 weeks № of participants: 2139 (1 RS, 7 NRS) | The mean pre-dialysis serum albumin ranged from **3.10-4.05** g/dl | MD **0.02 g/dl lower** (0.07 lower to 0.03 higher) | ⨁⨁⨁⨁ high | Theranova results in little to no difference in pre-dialysis serum albumin after 24 weeks follow-up. |

#### Explanations

A. Small overall sample size; optimal information size criterion not met.

B. Small overall sample size and the confidence interval includes no effect.

C. Estimate prone to risk of bias due to patient attrition.

D. Downgraded 2 levels for imprecision with only very small sample size and confidence interval crossing no effect.

### Table 3 – Summary of findings – middle molecules

| Outcome № of participants  (studies) | **Anticipated absolute effects (95% ci)** | | Certainty | What happens |
| --- | --- | --- | --- | --- |
|  | **Without Theranova** | **Difference** |  |  |
| **B2M removal (mg)** follow up: range 2 weeks to 8 weeks № of participants: 152 (4 RS) | - | SMD **1.83 SD higher** (0.02 higher to 3.64 higher) | ⨁⨁⨁⨁ high ^b^ | Theranova results in a large increase in B2M removal. |
| **B2M removal (mg)** № of participants: 16 (1 NRS) | - | SMD **1.4 SD higher** (0.42 higher to 2.38 higher) | ⨁⨁⨁⨁ high ^a,c^ | Theranova results in a large increase in B2M removal. |
| **B2M reduction ratio (%)** follow up: range 2 weeks to 26 weeks № of participants: 323 (7 RS) | The mean B2M reduction ratio (%) ranged from **46-77** % | MD **8.0 % higher** (2.8 higher to 13.2 higher) | ⨁⨁⨁⨁ high ^d^ | Theranova increases B2M reduction ratio. |
| **B2M reduction ratio (%)** follow up: range 2 weeks to 52 weeks № of participants: 174 (7 NRS) | The mean B2M reduction ratio (%) ranged from **48-75** % | MD **11.0 % higher** (9.5 higher to 12.5 higher) | ⨁⨁◯◯ low ^e,f^ | Theranova may increase B2M reduction ratio. |
| **Pre-dialysis B2M**  Subgroup with < 12 weeks follow-up follow up: 8 weeks № of participants: 32 (1 RS) | - | SMD **0.36 SD higher** (0.33 lower to 1.06 higher) | ⨁⨁◯◯ low | Theranova may result in little to no difference in pre-dialysis B2M over the short-term (< 3 months). |
| **Pre-dialysis B2M**  Subgroup with ≥12 weeks follow-up follow up: range 12 weeks to 26 weeks № of participants: 403 (5 RS) | - | SMD **0.54 SD lower** (1 lower to 0.08 lower) | ⨁⨁⨁◯ moderate ^g^ | Theranova likely reduces pre-dialysis B2M after 3 months of treatment. |
| **Pre-dialysis B2M** follow up: range 24 weeks to 52 weeks № of participants: 438 (6 NRS) | - | SMD **0.43 SD lower** (0.84 lower to 0.002 lower) | ⨁⨁⨁⨁ high | Theranova reduces pre-dialysis B2M slightly. |
| **Myoglobin removal** follow up: 2 weeks № of participants: 120 (3 RS) | - | SMD **2.9 SD higher** (1.31 higher to 4.49 higher) | ⨁⨁⨁⨁ high | Theranova likely results in a large increase in myoglobin removal. |
| **Myoglobin removal** follow up: 2 weeks № of participants: 16 (1 NRS) | - | SMD **1.09 SD higher** (0.11 higher to 2.07 higher) | ⨁⨁◯◯ low ^a^ | Theranova likely results in a large increase in myoglobin removal. |
| **Myoglobin reduction ratio (%)** follow up: range 2 weeks to 26 weeks № of participants: 242 (5 RS) | The mean myoglobin reduction ratio (%) ranged from **8-45** % | MD **30.26 % higher** (15.5 higher to 45.03 higher) | ⨁⨁⨁⨁ high ^h^ | Theranova results in large increase in myoglobin reduction ratio. |
| **Myoglobin reduction ratio (%)** follow up: range 2 weeks to 52 weeks № of participants: 118 (6 NRS) | The mean myoglobin reduction ratio (%) ranged from **12-44** % | MD **27.62 % higher** (24.29 higher to 30.95 higher) | ⨁⨁⨁⨁ high | Theranova results in large increase in myoglobin reduction ratio. |
| **Pre-dialysis myoglobin** follow up: 26 weeks № of participants: 130 (2 RS) | - | SMD **0.51 SD lower** (0.85 lower to 0.16 lower) | ⨁⨁⨁◯ moderate ^i^ | Theranova likely reduces pre-dialysis myoglobin. |
| **Pre-dialysis myoglobin** follow up: 26 weeks № of participants: 82 (1 NRS) | - | SMD **0.12 SD lower** (0.55 lower to 0.31 lower) | ⨁⨁⨁◯ moderate ^a^ | Theranova likely reduces myoglobin pre-hemodialysis slightly. |
| **Kappa FLC removal** follow up: 2 weeks № of participants: 78 (2 RS) | - | SMD **3.89 SD higher** (3.45 higher to 4.33 higher) | ⨁⨁⨁⨁ high | Theranova results in large increase in kappa FLC removal. |
| **Kappa FLC removal** follow up: range 2 weeks to 3 weeks № of participants: 28 (2 NRS) | - | SMD **1.43 SD higher** (0.69 higher to 2.17 higher) | ⨁⨁⨁◯ moderate ^a^ | Theranova likely results in large increase in kappa FLC reduction ratio. |
| **Kappa FLC reduction ratio (%)** follow up: range 2 weeks to 26 weeks № of participants: 249 (5 RS) | The mean kappa FLC reduction ratio (%) ranged from **53-72** % | MD **14.85 % higher** (8.27 higher to 21.43 higher) | ⨁⨁⨁⨁ high ^h^ | Theranova results in large increase in kappa FLC reduction ratio. |
| **Kappa FLC reduction ratio (%)** follow up: range 2 weeks to 52 weeks № of participants: 106 (4 NRS) | The mean kappa FLC reduction ratio (%) ranged from **35-54** % | MD **20.78 % higher** (9.44 higher to 32.13 higher) | ⨁⨁⨁◯ moderate ^j,k^ | Theranova likely results in large increase in kappa FLC reduction ratio. |
| **Pre-dialysis kappa-FLC** follow up: range 12 weeks to 26 weeks № of participants: 403 (5 RS) | - | SMD **0.39 SD lower** (0.61 lower to 0.16 lower) | ⨁⨁⨁⨁ high | Theranova reduces pre-dialysis kappa FLC slightly. |
| **Pre-dialysis kappa-FLC** follow up: range 24 weeks to 52 weeks № of participants: 398 (4 NRS) | - | SMD **0.26 SD lower** (0.79 lower to 0.27 higher) | ⨁⨁◯◯ low ^l,m^ | Theranova may result in little to no difference in pre-dialysis kappa FLC. |
| **Lambda FLC removal** follow up: 2 weeks № of participants: 118 (3 RS) | - | SMD **2.16 SD higher** (1.8 higher to 2.52 higher) | ⨁⨁⨁⨁ high | Theranova results in large increase in lambda FLC removal. |
| **Lambda FLC removal** follow up: range 2 weeks to 3 weeks № of participants: 130 (2 NRS) | - | SMD **3.71 SD higher** (2.97 higher to 4.45 higher) | ⨁⨁⨁⨁ high | Theranova results in large increase in lambda free light chain removal . |
| **Lambda FLC reduction ratio (%)** follow up: range 2 weeks to 26 weeks № of participants: 450 (7 RS) | The mean lambda FLC reduction ratio (%) ranged from **13-41** % | MD **20.85 % higher** (15.53 higher to 26.16 higher) | ⨁⨁⨁⨁ high | Theranova increases lambda-FLC reduction ratio. |
| **Lambda FLC reduction ratio (%)** follow up: range 2 weeks to 52 weeks № of participants: 106 (4 NRS) | The mean lambda FLC reduction ratio (%) ranged from **13-51** % | MD **23.47 % higher** (10.01 higher to 36.92 higher) | ⨁⨁⨁◯ moderate ^j^ | Theranova likely results in a large increase in lambda FLC reduction ratio. |
| **Pre-dialysis lambda-FLC** follow up: range 12 weeks to 26 weeks № of participants: 402 (5 RS) | - | SMD **0.53 SD lower** (0.9 lower to 0.17 lower) | ⨁⨁⨁⨁ high ^k^ | Theranova reduces pre-dialysis lambda-FLC. |
| **Pre-dialysis lambda-FLC** follow up: range 24 weeks to 52 weeks № of participants: 398 (4 NRS) | - | SMD **0.34 SD lower** (0.54 lower to 0.14 lower) | ⨁⨁⨁⨁ high | Theranova reduces lambda free light chain pre-hemodialysis slightly. |

#### Explanations

A. Small overall sample size; optimal information size criterion not met.

B. I2=97%, but fully explained by measurement method - removal was higher when measured by plasma clearance vs. Dialysate quantitation.

C. SMD > 0.8 considered a large treatment effect. Rated up one level.

D. Although i2 was 99%, heterogeneity was explained by baseline removal ratio (larger effect if removal ratio was <70%), and was further explained by study duration (effect was attenuated with longer-term treatment).

E. Of 7 included studies, bias due to confounding was present in 2 studies, selection bias in one study, attrition bias in one study, missing data in one study,.

F. I2=95% and confidence intervals do not overlap.

G. I2> 50% and confidence intervals do not overlap.

H. Inconsistency explained by baseline removal ratio such that studies with lower baseline rr had larger effects with Theranova.

I. I2=82% with opposite directions of effect.

J. Estimate might have been prone to rob due to attrition and possible selection bias.

K. Inconsistency explained by duration of follow-up with a larger treatment effect with longer-term treatment.

L. I2=85%, not explained by study duration.

M. The effect estimate includes no effect.

### Table 4 – Summary of findings – inflammatory markers and cytokines

| Outcome № of participants  (studies) | **Anticipated absolute effects (95% ci)** | | Certainty | What happens |
| --- | --- | --- | --- | --- |
|  | **Without Theranova** | **Difference** |  |  |
| **Il-6 reduction ratio (%)** follow up: 26 weeks № of participants: 80 (1 RS) | The mean IL-6 reduction ratio (%) was **9.5** % | MD **0.2 % lower** (3.44 lower to 3.04 higher) | ⨁⨁⨁◯ moderate ^a^ | Theranova likely results in little to no difference in interleukin-6 reduction ratio (%). |
| **Il-6 reduction ratio (%)** follow up: 2 weeks № of participants: 16 (1 NRS) | The mean IL-6 reduction ratio (%) was **-65** % | MD **89.04 % higher** (59.43 lower to 238.23 higher) | ⨁⨁◯◯ low ^c^ | Theranova may result in little to no difference in IL-6 reduction ratio. |
| **Pre-dialysis IL-6** follow up: range 12 weeks to 26 weeks № of participants: 354 (4 RS) | - | SMD **0.04 SD higher** (0.17 lower to 0.25 higher) | ⨁⨁⨁◯ moderate ^b^ | Theranova likely results in little to no difference in pre-dialysis IL-6. |
| **Pre-dialysis IL-6** follow up: range 24 weeks to 26 weeks № of participants: 113 (2 NRS) | - | SMD **0.21 SD lower** (0.59 lower to 0.1 higher) | ⨁⨁◯◯ low ^a,d^ | Theranova may result in little to no difference in pre-dialysis IL-6. |
| **Il-6 mRNA expression** follow up: 12 weeks № of participants: 46 (1 RS) | The mean IL-6 expression was **100** % | MD **8.8 % lower** (10.2 lower to 7.4 lower) | ⨁⨁⨁◯ moderate ^a^ | Theranova likely reduces IL-6 expression. |
| **TNF-**α **reduction ratio (%)** follow up: 26 weeks № of participants: 80 (1 RS) | The mean TNF-α reduction ratio (%) was **26** % | MD **7.67 % higher** (4.7 higher to 10.64 higher) | ⨁⨁⨁◯ moderate ^a^ | Theranova likely increases TNF-α reduction ratio. |
| **TNF-**α **pre-dialysis**  follow up: range 12 weeks to 26 weeks № of participants: 304 (3 RS) | - | SMD **0.48 SD lower** (0.91 lower to 0.04 lower) | ⨁⨁⨁◯ moderate ^a^ | Theranova likely reduces pre-dialysis TNF-α. |
| **TNF-**α **mRNA expression** follow up: 12 weeks № of participants: 46 (1 RS) | The mean TNF-α expression was **100** % | MD **15 % lower** (19.6 lower to 10.4 lower) | ⨁⨁⨁◯ moderate ^a^ | Theranova likely reduces TNF-α expression. |
| **C-reactive protein**  follow up: 12 weeks № of participants: 145 (2 RS) | - | SMD **0.04 SD higher** (0.37 lower to 0.29 higher) | ⨁⨁⨁◯ moderate ^a^ | Theranova likely results in little to no difference in C-reactive protein. |
| **C-reactive protein** follow up: range 26 weeks to 52 weeks № of participants: 1940 (5 NRS) | - | SMD **0 SD**  (0.23 lower to 0.22 higher) | ⨁⨁⨁⨁ high | Theranova results in little to no difference in C-reactive protein. |

#### Explanations

A. Small overall sample size; optimal information size criterion not met.

B. Small overall sample size and the confidence interval includes no effect.

C. Downgraded 2 levels for imprecision with only very small sample size and confidence interval crossing no effect.

D. I2=95% with qualitatively different estimates of effect.
